# Two Squares in a Barrel: An Axially Disubstituted Conformationally Rigid Aliphatic Binding Motif for Cucurbit[6]uril

**DOI:** 10.1021/acs.joc.3c01556

**Published:** 2023-10-26

**Authors:** Kristýna Jelínková, Aneta Závodná, Jiří Kaleta, Petr Janovský, Filip Zatloukal, Marek Nečas, Zdeňka Prucková, Lenka Dastychová, Michal Rouchal, Robert Vícha

**Affiliations:** † Department of Chemistry, Faculty of Technology, Tomas Bata University in Zlín, Vavrečkova 5669, Zlín 760 01, Czech Republic; ‡ Institute of Organic Chemistry and Biochemistry of the Czech Academy of Sciences, Flemingovo náměstí 2, Praha 16000, Czech Republic; § Department of Chemistry, Faculty of Science, Masaryk University, Kotlářská 2, Brno 602 00, Czech Republic

## Abstract

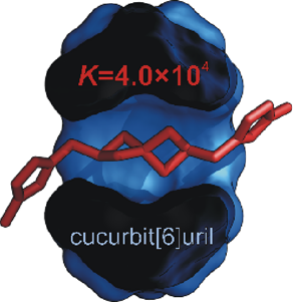

Novel binding motifs suitable for the construction of multitopic guest-based molecular devices (e.g., switches, sensors, data storage, and catalysts) are needed in supramolecular chemistry. No rigid, aliphatic binding motif that allows for axial disubstitution has been described for cucurbit[6]uril (CB6) so far. We prepared three model guests combining spiro[3.3]heptane and bicyclo[1.1.1]pentane centerpieces with imidazolium and ammonium termini. We described their binding properties toward CB6/7 and α-/β-CD using NMR, titration calorimetry, mass spectrometry, and single-crystal X-ray diffraction. We found that a bisimidazolio spiro[3.3]heptane guest forms inclusion complexes with CB6, CB7, and β-CD with respective association constants of 4.0 × 10^4^, 1.2 × 10^12^, and 1.4 × 10^2^. Due to less hindering terminal groups, the diammonio analogue forms more stable complexes with CB6 (*K* = 1.4 × 10^6^) and CB7 (*K* = 3.8 × 10^12^). The bisimidazolio bicyclo[1.1.1]pentane guest forms a highly stable complex only with CB7 with a *K* value of 1.1 × 10^11^. The high selectivity of the new binding motifs implies promising potential in the construction of multitopic supramolecular components.

## Introduction

**Figure 1:**
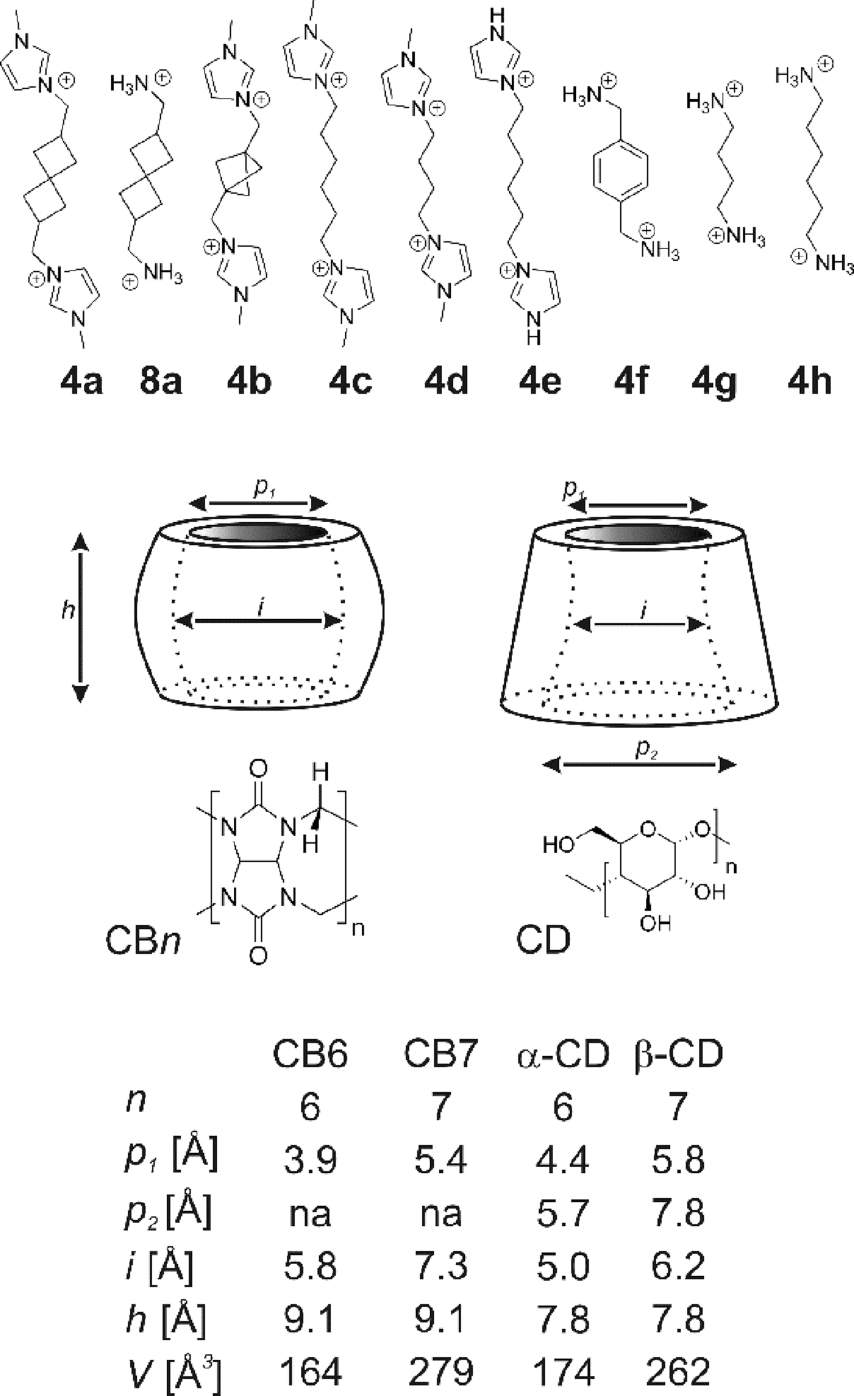
Guests and hosts under consideration in this study.

Within recent decades, pseudorotaxane and rotaxane structures have been extensively studied as molecular catalysts,^1^ switches,^2^ or sensors.^3^ Additional functions of such systems, e.g., responsivity to different stimuli or allosteric regulation of catalytic activity, need more complex components. Considering the host–guest concept in organocatalysts, nature has put a higher complexity on the host molecules, e.g., proteins, whereas artificial systems are more achievable by employing composite guest molecules that consist of multiple binding sites.^4^ Thus, different macrocyclic hosts can be used for active site construction and activity modification and/or regulation.^5^ Indeed, such multitopic guests must be combined with host molecules with a high range of binding affinities and high selectivity toward different binding motifs to allow the existence of several distinct arrangements. The optimal hosts for regulation purposes are cucurbit[*n*]urils (CB*n*s, Figure 1), since the binding constants of these highly symmetrical and rigid artificial molecular containers reach the highest ever reported values up to 10^17^ M^–1^.^6^ Several rigid binding motifs derived from adamantane,^7^ bicyclo[2.2.2]octane,^7a^ cubane,^8^ diamantane,^6,9^ or ferrocene^10^ were reported for CB7 and CB8 to demonstrate extraordinarily high affinity and/or excellent selectivity. In contrast, the binding motifs for the smaller member of the family, CB6, are limited to the linear aliphatic chains and derivatives of benzene. In addition to CB*n*s, cyclodextrins (CDs, Figure 1) are favored macrocycles for the construction of catalytic devices due to their natural origin, inherent chirality, and well-established methods for selective modification.^11^

In the design of binding motifs for macrocyclic hosts, several important geometrical parameters should be considered. First, the length of the binding motif determines which part of the ligand is located inside the macrocycle cavity. This parameter is highly important for the guests decorated with functional groups that can interact with the portals of the macrocycle. For instance, Mock and Shih^12^ demonstrated that the six-membered alkyl chain in alkyl diammonium salts has the optimal length to display the highest affinity toward CB6 in comparison with other shorter or longer linear alkyl diammonium salts. Second, the bulkiness of the ligand must reflect a compromise between steric hindrance and voids inside the cavity. The importance of the appropriate volume of the guest binding site can be demonstrated by a comparison of linear hexane-1,6-diammonium (**4h**) and 4,9-diammoniodiamantane. Despite the similar distance between cationic groups (8.8 and 7.6 Å), the former guest displays an affinity toward CB7 in the order of 10^6^ M^–1^ (ref (13)),whereas the latter forms a 10^5^ times tighter complex.^6^ An effective diameter of the binding site, i.e., the highest van der Waals diameter orthogonal to the long axis of the binding motif, is a geometric parameter closely related to the bulkiness of the binding site. Despite the strong correlation of this effective diameter with the ability of the guest to pass through the portal of the rigid guest, e.g., cucurbit[*n*]urils, this parameter is rarely discussed in the literature as threading through the portal is related to the kinetics while geometric complementarity with the interior cavity is related to the thermodynamics of the complexation.^14^

We considered the above-mentioned geometric parameters and designed novel rigid, aliphatic, and axially disubstituted binding motifs for CB6, which are needed for our recent work on molecular devices based on multitopic guests. In this paper, we report the synthesis and supramolecular behavior of three model dicationic guests derived from bicyclo[1.1.1]pentane and spiro[3.3]heptane (Figure 1). The latter forms a remarkably strong complex with CB6 to provide a reasonable aliphatic rigid scaffold for the design and construction of supramolecular multitopic guests.

## Results and Discussion

### Chemistry

**Scheme 1:**
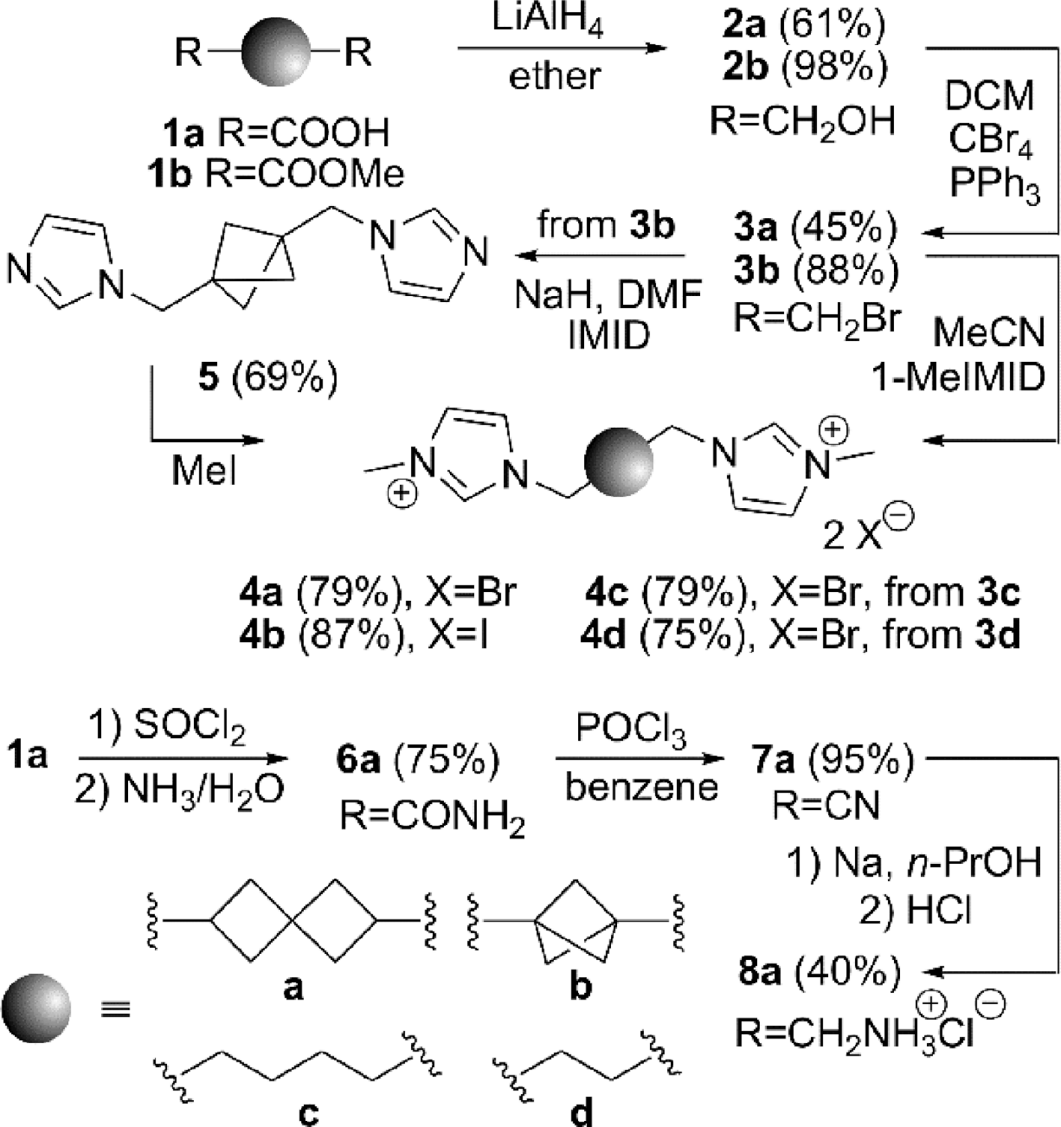
Synthesis of Guests **4a**–**4d** and **8a** (IMID Refers to Imidazole)

The chemical transformations that lead to the required guests **4a**–4**d** are shown in Scheme 1. Dicarboxylic acid **1a** and dimethyl ester **1b**, which were prepared according to previously published procedures,^15^ were reduced using LiAlH_4_ and treated under Appel conditions^16^ to obtain dibromo compounds **3a** and **3b**, respectively. The bromide **3a** and commercial **3c** and **3d,** were reacted with 1-methylimidazole in MeCN to provide the corresponding bisimidazolium salts in yields of 75–79%. A 1,3-disubstitution of imidazole can be easily achieved via a sequence of nucleophilic substitution and quaternization. Since the quaternization step usually provides lower yields with weak and moderate alkylating agents, the S_N_ with sodium imidazolide proceeds very well. Therefore, we prepared bicyclo[1.1.1]pentane ligand **4b** in two steps from **3b**. The two imidazole rings were introduced to the structure first, and the final **4b** was subsequently obtained by a reaction of the intermediate **5** with MeI. The structure of guest **4b** was verified using single-crystal X-ray diffraction analysis (Table S2, Figure S104). The diamine **8a** was prepared from dicarboxylic acid **1a** via acyl chloride, dicarboxamide **6a**, and dinitrile **7a** in an overall yield almost of 30%. The last step of the previously reported procedure^15a^ had to be significantly modified. As the reduction of dinitrile **7a** with BH_3_ in THF (no reaction) or with LiAlH_4_ in Et_2_O (poor solubility of **7a**) or in THF (isolation issues) failed, sodium chips in *n*-propanol were successfully employed.

**Figure 2:**
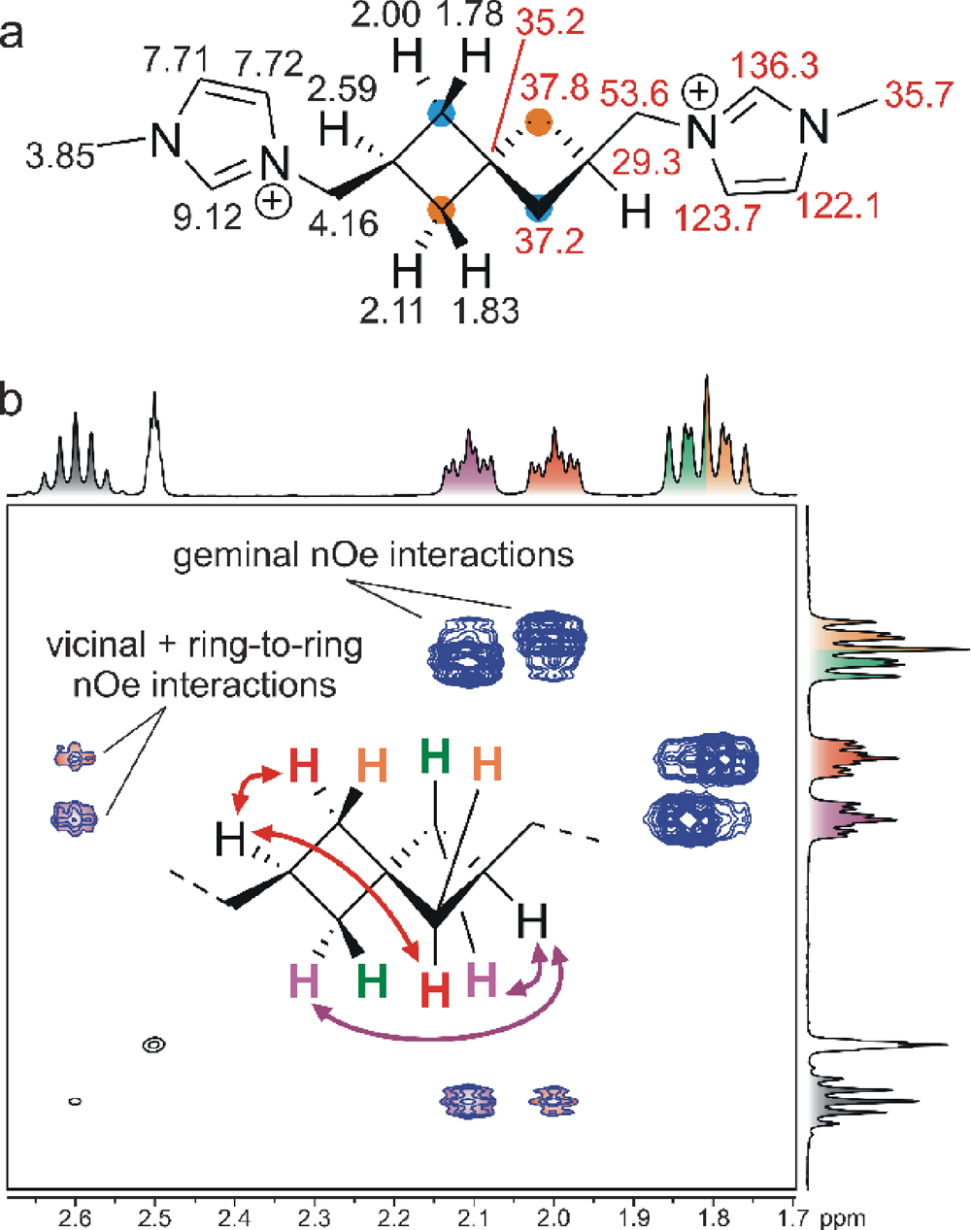
(a) Full assignment of signals in the ^1^H NMR (black) and ^13^C NMR (red) spectrum of guest **4a**. Symmetrically equivalent CH_2_ groups of spiroheptane are marked with blue and orange spots. The chemical shift δ is given in ppm. (b) A portion of the ROESY spectrum (400 MHz, 303 K, DMSO-*d*_6_) of guest **4a**. The inset drawing shows the signal assignment along with the key NOE contacts.

Spiro[3.3]heptane with two identical substituents in positions 2 and 6 (e.g., the guests **4a** and **8a** and their precursors) has *C*_2_ symmetry and displays an axial chirality. Therefore, all H atoms at one cyclobutane ring are chemically nonequivalent, and full assignment of signals in NMR spectra is somewhat challenging^17^ and rarely reported. Figure 2a shows the complete assignment of the ^1^H and ^13^C signals of guest **4a** based on 2D NMR experiments (^1^H–^13^C-HSQC and ^1^H–^1^H-ROESY). A portion of the key ROESY spectrum is shown in Figure 2b. As the figure illustrates, all five H atoms of the cyclobutane ring are nonequivalent and provide well-separated signals. The signal of the single C**H** was observed as an apparent septet at 2.59 ppm, and the signals of CH_2_ H atoms appear as four multiplets in an area of 1.7–2.2 ppm. Strong NOE cross-peaks related to the short distance between geminal H atoms allowed us to identify the pairs of H atoms for each CH_2_ spectra in concert with the analysis of the HSQC spectrum. Subsequently, the NOE cross-peaks related to the interaction between CH(2.59 ppm) and H atoms of CH_2_ indicated the *cis*-vicinal orientation. Finally, we attributed the different intensities of these cross-peaks to the contribution of the symmetrically related H atoms from the adjacent cyclobutane ring. Whereas the *cis*-vicinal distances of H(2.59 ppm)···H(2.00 ppm) and H(2.59 ppm)···H(2.11 ppm) are essentially equal, the distances from H(2.59 ppm) to the corresponding H atoms from the second ring differ by 1.5 Å. The same trend was observed for the *trans-*vicinal H atoms (not shown in Figure 2 due to the low magnitude of the cross-peaks). It should be noted that the H atoms of the exocyclic methylene bridge display a sharp doublet in the ^1^H NMR spectrum (4.16 ppm, not shown in Figure 2) despite their diastereotopic nature.

### Studies on Supramolecular Properties

Although we originally wanted an aliphatic rigid central (axially disubstituted) binding motif for CB6, we also involved CB7, α-CD, and β-CD in our binding studies to determine the selectivity of our model guests within a broader spectrum of common hosts. In addition to the spiroheptane and bicyclopentane guests, we examined bisimidazolium salts with butane-1,4-diyl and hexane-1,6-diyl centerpieces, i.e., guests **4d** and **4c**, respectively. The relevant guest pairs have very similar distances between cationic parts (*d*_**4a**_ = 1.01 × *d*_**4c**_, *d*_**4b**_ = 1.08 × *d*_**4d**_, according to MM2-optimized molecular models); however, linear aliphatic chains have minimal bulkiness and high flexibility. Therefore, we used guests **4c** and **4d** as models to estimate the influence of the increased volume and rigidity on binding properties. We employed NMR spectroscopy and mass spectrometry to describe the binding behavior of our guests. Simultaneously, the association constants were quantified using isothermal titration calorimetry (ITC). All mixtures were tested to be in thermodynamic equilibrium. If no binding was observed immediately after mixing, the solutions were warmed (60–80 °C) for several days. The mixtures after titration experiments were stored at ambient temperature for several months. Unchanged ^1^H NMR spectra indicated no additional slow processes.

We observed no complexation-induced shifts (CIS) in the ^1^H NMR spectra during titration of the guests **4a**–**4d** and **8a** with α-CD and β-CD, with the exception of the **4a**/β-CD system. In this case, the separation of aromatic signals was observed to indicate a weak interaction in fast mode on the NMR time scale (Figure S51). Using ITC, we determined the binding constant *K* = 135 M^–1^ for **4a**@β-CD, whereas other guests with both CDs displayed no binding. Although the NMR and ITC data indicated no binding, we detected unambiguous signals related to the [G^2+^@α-/β-CD]^2+^ complexes in the first-order electrospray mass spectra (for mass spectra, see the Supporting Information). These results indicate that weak complexes (log *K* < 2) with cyclodextrins can be present in water solutions.

In contrast, all examined guests formed moderate to highly stable complexes with cucurbit[*n*]urils. Initially, we tested the influence of imidazolium cations on the complex stability. We proved that bisimidazolium salts with linear alkane spacers match the trend of the *K* values described by Mock for diammonium salts **4g** and **4h**,^12^ i.e., the guest **4c** with a hexane-1,6-diyl centerpiece displays a higher affinity toward CB6 than the shorter **4d**. Indeed, the bulkier imidazolium moiety lowered the affinity toward CB6 with a narrow interior cavity. This effect is much more conspicuous in the case of the shorter guest **4d** (see Table 1). Concerning CB7, the *K* values are very similar for guests with a hexamethylene centerpiece (**4c** and **4h**) and even slightly higher for bisimidazolium salt **4d** in comparison with diammonium salt **4g**, both having tetramethylene centerpieces. We infer that a more delocalized imidazolium cation provides more efficient ion-dipole interactions in the wider portal of CB7 and thus compensates for a steric hindrance.

**Table 1:** Association Constants (Log *K*)

	log *K*^a^			
guest	CB6^b^	CB7	α-CD	β-CD
**4a**	4.6	12.1	no binding	2.13
**8a**	6.1^c^	12.6	no binding	no binding
**4b**	no binding	11.0	no binding	no binding
**4c**	7.7	9.4	no binding	no binding
**4d**	4.6	7.4	no binding	no binding
**4g**	9.2; 6.58^d^	6.3; 5.48^e^; 6.27^f^	1.18^g^	not known
**4h**	9.9; 6.91^d^; 6.59^j^; 8.65^h^	9.3; 9.32^g^; 7.95^h^;6.17^i^; 5.67^d^; 9.11^f^	1.81^g^	not known
**4f**	2.74^h^	7.23^i^; 9.26^h^; 10.34^f^	not known	not known

a Determined using ITC, in water at 303 K if not stated otherwise.

b All measurements with CB6 were done in 50 mM NaCl if not stated otherwise.

c NMR, 50 mM NaCl in D_2_O, 303 K.

d ITC, 50% HCOOH, 298 K, ref (18).

e ITC, 0.01 M NH_4_OAc buffer, pH = 6, 303 K, ref (19).

f ITC, water, 298 K, ref (20).

g ITC, water, 298 K, ref (10).

h NMR, 0.05 M CD_3_COONa buffer, pH = 4.7, 298 K, ref (21)**.**

i NMR, 0.1 M Na_3_PO_4_ buffer, pH = 7.4, ref (13).

j ITC, 0.01 M NH_4_OAc buffer, pH = 7, 298 K, ref (22).

Unfortunately, bicyclo[1.1.1]pentane-based guest **4b** showed no binding toward CB6, according to NMR and ITC data. Even MS did not show any signal related to the expected complex, although this method usually allows for the detection of very weak complexes. We attribute this behavior to the combination of disadvantageous geometrical features, i.e., a short N^+^···N^+^ distance comparable to **4g**, bulky imidazolium cations, and a rigid centerpiece. However, guest **4b** forms a highly stable inclusion complex with CB7 (*K* = 1.1 × 10^11^ M^–1^).

Also, the spiro[3.3]heptane-based guest **4a** constitutes a very stable complex with CB7. According to ITC data, the stability of the complex **4a**@CB7 (*K*_303 K_ = 1.2 × 10^12^ M^–1^) is comparable with the well-known, single cationic, adamantane-1-amine hydrochloride^7a^ (*K*_298 K_ = 1.70 × 10^14^ M^–1^). The ^1^H NMR spectra clearly show a new set of signals with the titration experiment to indicate a slow exchange mode on the NMR time scale. The shielding of the H atoms at the spiroheptane skeleton and methylene bridges and simultaneous deshielding of the terminal CH_3_ groups point to the manner of inclusion of the complex with spiroheptane inside the CB7 cavity. Note that the signal of H atoms at the methylene bridge, which appears in the spectrum of the free **4a** as one sharp doublet, was split into two doublets of doublets, i.e., a pattern expected for methylene C**H**_2_—CH with diastereotopic H atoms. However, we attribute this splitting to the hindered rotation of the substituents at the spiroheptane skeleton within the complex rather than to the inherent diastereotopicity of the methylene H atoms.

The extraordinarily high stability of cucurbit[*n*]urils, particularly CB7, can be rationalized by the synergic effect of the nonclassical hydrophobic effect (releasing of high-energy water molecules from macrocycle cavity), ion–dipole interactions between cationic guests and carbonyl O atoms at CB*n*’s portals and dispersion forces between the geometrically complementary guest and cavity interior. In this sense, the moderate affinity of our new guests can be rationalized by their geometric parameters. Compounds **4h** (*K*_CB7_ = 4.5 × 10^8^ M^–1^, 50 mM NaOAc buffer)^21^ and 4,9-bis(trimethylammonio)diamantane (*K*_CB7_ = 1.9 × 10^15^ M^–1^, 50 mM NaOAc buffer)^6^ represent limit structures having similar N···N distances (8.8 and 7.6 Å) and volumes as different as possible. The affinities of **4a**, **4b**, and **8a** toward CB7 in a range of 1.0–20.0 × 10^11^ M^–1^ can be attributed to the intermediate bulkiness of the central hydrocarbon skeleton, which decreases the contribution of dispersion interactions inside the cavity.

High stability of complexes **4a**, **4b**, and **8a** with CB7 was also demonstrated in MS spectra where signals related to the [G^2+^@CB7]^2+^ cations strongly predominated (Figures S79, S83, and S95).

**Figure 3:**
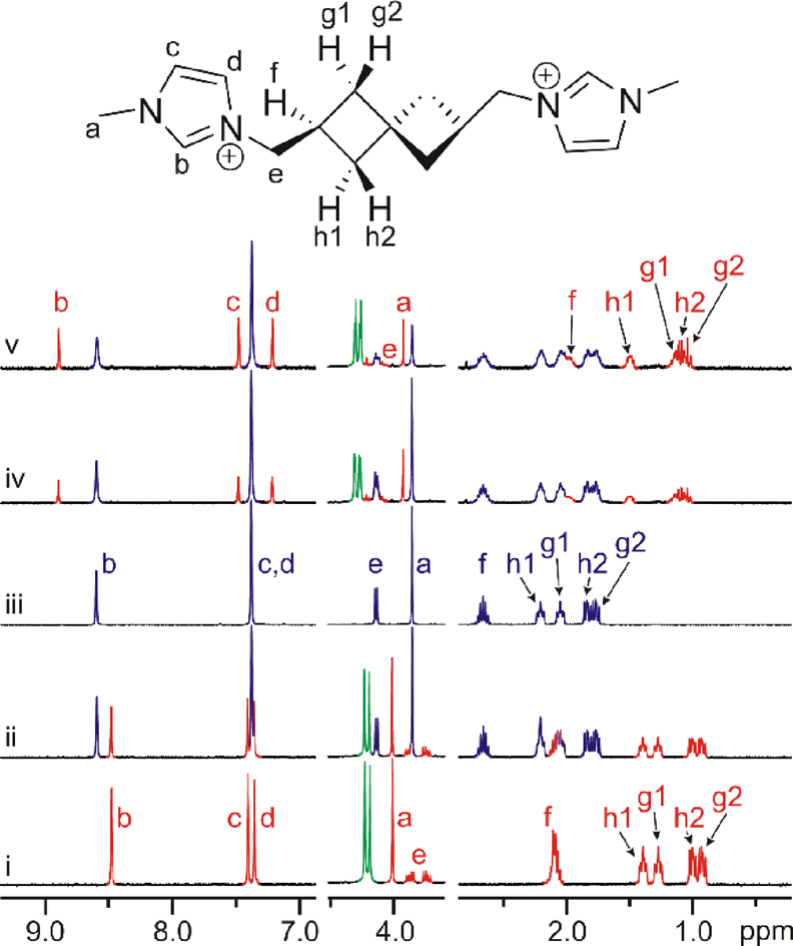
Portions of ^1^H NMR (303 K, 400 MHz) spectra recorded within titrations of guest **4a** with CB6 (50 mM NaCl in D_2_O) and CB7 (D_2_O). (i) 1 equiv of CB7, (ii) 0.5 equiv of CB7, (iii) free **4a**, (iv) 0.5 equiv of CB6, 1 equiv of CB6. The signals of the free guest, complexed guest, and CB*n* are shown in blue, red, and green, respectively. Spectra are not on a scale.

**Figure 4:**
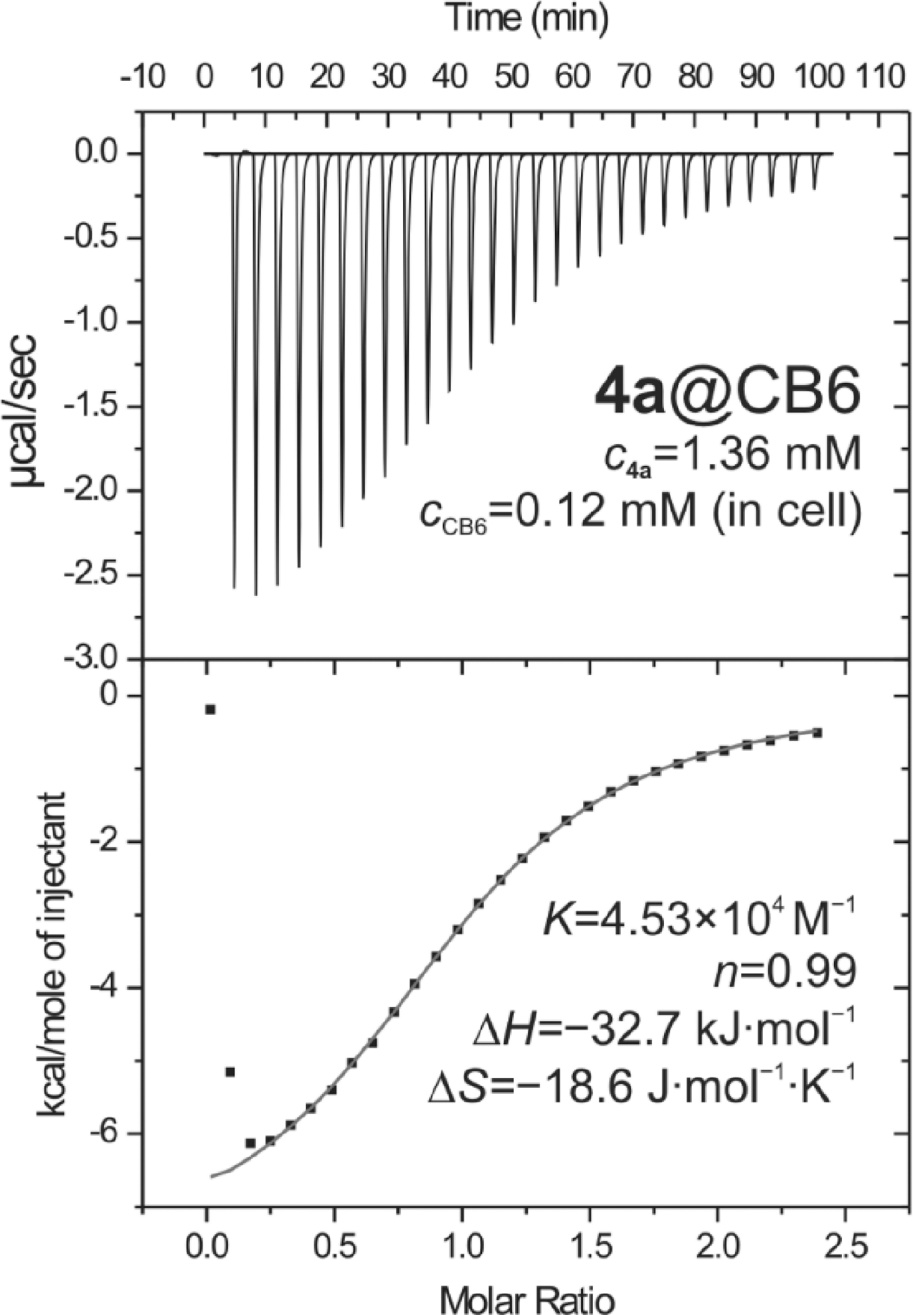
Isothermal titration calorimetry data for guest **4a** and CB6 in 50 mM NaCl in water.

Subsequently, we examined the ability of guest **4a** to form an inclusion complex with CB6. Analogous to the titration with CB7, we observed a new set of signals in the ^1^H NMR spectrum and the complexation-induced shifts indicate the positioning of the spiroheptane inside the CB6 cavity (Figure 3, lines iii to v). The presence of the signals of the free guest when one mol equivalent of CB6 was added demonstrated a lower affinity of **4a** toward CB6. The value of binding constant *K* = 4.0 × 10^4^ M^–1^ was determined using ITC. The raw data and corresponding binding isotherm can be seen in Figure 4. Similar to the case for the **4a**@CB7 complex, a strong signal of the analogous [**4a**^**2+**^@CB6]^2+^ dication was observed in the mass spectrum (Figure S78). To reveal the role of the nature of cationic moieties, diamino spiro[3.3]heptane analogue **8a** was prepared. It was found that the respective binding constants of **8a** with CB6 and CB7 are 34× and 3.2× greater than those for **4a**. These results indicate that the bulkiness of the cationic moieties plays a more significant role in the case of CB6. In addition, the formation of inclusion complex **8a**@CB6 was found to be significantly slower in comparison with **4a**@CB6. Whereas the system consisting of **4a** and CB6 equilibrated within seconds, the mixture containing **8a** and CB6 needed hours at 303 K to reach an equilibrium. As a kinetic curve (Figure S71) does not match either the monomolecular or bimolecular model, we suppose a two-step process. In the first step, a small portion of an external complex is formed in a fast equilibrium, as indicated by an additional set of signals for CB6 in the ^1^H NMR spectrum (Figure S70). Subsequently, the inclusion complex slowly arises.

According to ITC measurements (complete thermodynamic data are given in Table S1), all examined complexations are enthalpy-driven. In almost all cases, the enthalpic gain is accompanied by a large positive change in entropy. Considering CB6/CB7 complexes, there are only two pairs, i.e., **4a**@CB6 and **4c**@CB6, displaying entropic loss. A clear trend can be observed in the CB6 series where imidazolium cations and rigid centerpiece support entropic loss, whereas ammonium cations and flexible linkers contribute to entropic gain. These data suggest that hindering the free movement of the terminal groups plays a more significant role than rigidifying the central part of the ligand during complexation. Comparing our thermodynamic data with those previously published (Figure S103), it can be seen that our systems match an enthalpy–entropy compensation overcoming phenomenon, which was described previously for highly stable cucurbit[*n*]uril complexes with ferrocene, adamantane, and bicyclo[2.2.2]octane derivatives.^10^

**Figure 5:**
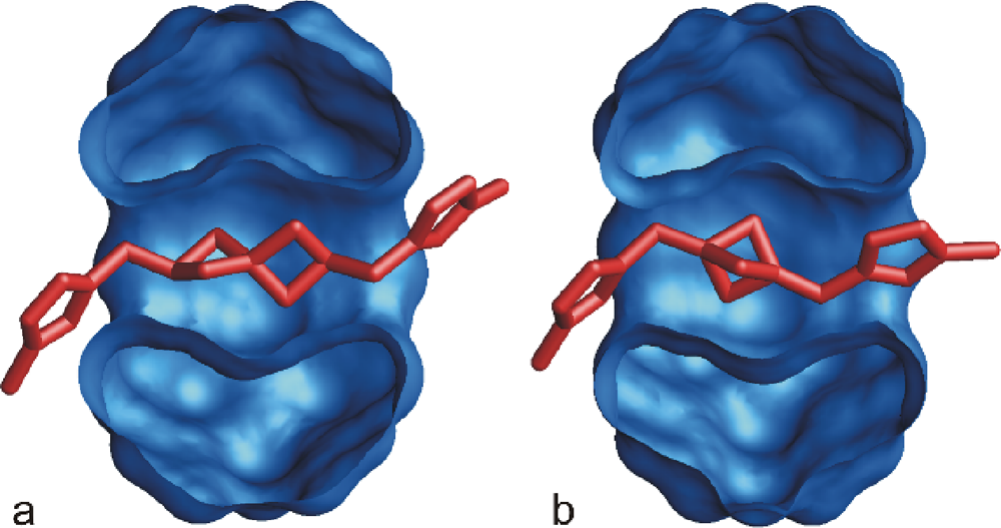
(a) Cross-section of a molecular model of **4a**@CB6 obtained using X-ray diffraction analysis. H atoms, disordered atoms, bromide counterions, and water molecules are omitted for clarity. (b) A cross-section of the molecular model of **4b**@CB6 obtained from molecular modeling (MM2/mmff94s). The guests and CB6 are shown as red sticks and blue surfaces, respectively.

In addition, we succeeded in growing a single crystal of complex **4a**@CB6, which was suitable for X-ray diffraction analysis. Although we observed a considerable positional disorder of the central part of the guest inside the CB6 cavity, the data allowed for picking up the molecular model and its geometrical parameters to demonstrate the manner of inclusion of the **4a**@CB6 complex, as shown in Figure 5a (for ORTEP, see Figure S105). The important geometric parameters of the complex are presented in Table 2.

**Table 2:**
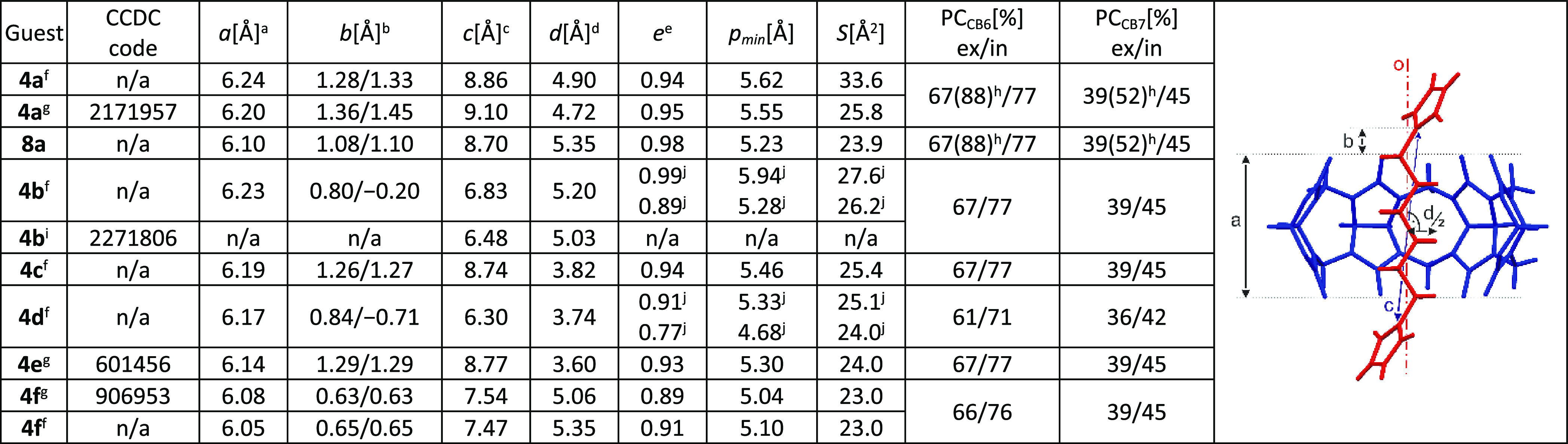
Geometric Parameters of the Studied CB6/7 Complexes

a *a* is the distance between portal O atoms’ mean planes, i.e., the height of CB6.

b *b* is the distance from the adjacent N atom to the portal O atoms’ mean plane (a negative value means that the location of the N atom is inside the cavity).

c *c* is the distance between the adjacent N atoms of the guest.

d *d* is the effective diameter calculated as the mean distance of H atoms from a line through C atoms of the central part of the guest (o).

e Ellipticity of the CB6 portal (for computational details, see the Supporting Information).

f Obtained from MM2-optimized models.

g Obtained from X-ray diffraction of the complex.

h If exocyclic methylenes were taken into account.

i Obtained from X-ray diffraction of the single guest.

j One portal is significantly deformed due to deeply buried imidazolium moiety; n/a means not applicable.

Considering spatial complementarity, a packing coefficient (PC) defined by Rebek can be used.^23^ For this purpose, the volumes of both the guest and the interior cavity of the host must be determined. The guest volume can be calculated according to a formula introduced by Zhao and co-workers.^24^ The interpretation of the interior cavity of CB*n*s and related volumes was discussed thoroughly by Nau et al.,^25^ who defined the “inner cavity” as an interior room covered by mean planes through portal O atoms. The inner cavity is relevant for the hydrophobic effect, whereas the bond dipole region is centered within each portal close to the covering mean planes. The optimal PC value was determined as 55% (voids in water), and a positive contribution of the hydrophobic effect was estimated for PC in the range of 30–75%. However, in the case of the guests that are larger than the host cavity, this approach is somewhat compromised due to the uncertainty regarding the question of what atoms of the guest should be taken into account, i.e., what guest atoms are actually inside the cavity. The PC values presented in Table 2 were calculated while considering the atoms within the inner cavity using X-ray diffraction data or MM2-computed models. Interestingly, very similar values of packing coefficients were obtained for guests **4a**, **8a**, **4b**, **4c**, and **4f**. Only guest **4d** has a lower PC value. This is in strong contrast to the significantly different binding constants of the examined compounds (Table 1). For instance, the respective *PC*_CB6_ values for **4c** and **4f** are 77 and 76%, whereas the *K*_CB6_ values differ by a factor of 10^5^. Therefore, it is clear that other geometrical parameters influence the binding more significantly.

The important geometric parameters, i.e., the distance between adjacent N atoms, the distance of these atoms from portal mean planes, and the effective diameter of the central part of the ligand, are summarized in Table 2 along with the PC values. We enriched the original set of ligands, **4a**–**4d** and **8a**, with two examples of typical guests, **4e** and **4f**. The structure of the inclusion complexes of these ligands with CB6 has been determined using X-ray diffraction by other authors^26^ and is available from The Cambridge Crystallographic Data Centre (CCDC, for reference numbers, see Table 2). It should be noted that the hexamethylene chain adopts various conformations inside the CB6 cavity, as was revealed by an extensive examination of X-ray data in CCDC.^27^ The guests in these structures varied in the *d* parameter (meaning explained below). Therefore, we selected structure **4e** with the most relaxed (i.e., all *anti*) hexamethylene chain as an example to represent the most likely structure of the hexamethylene centerpiece in the solution.

All of the examined guests have a cationic moiety (for imidazolium, the adjacent N atoms were taken into account) within the bond dipole region; however, the distance from the portal O atoms’ mean plane (Table 2, parameter *b*) is the highest for the spiroheptane guest **4a**. In contrast, the short central part of guests **4b** and **4d** forces one N atom to be inside the cavity (the negative *b* value in Table 2) if the second N atom occupies an optimal position within the opposite portal. This arrangement is supported by the MM2 model of complex **4b**@CB6, as shown in Figure 5b. We infer that the inappropriate length of the central part weakens the ion–dipole interaction within the portals, decreasing the *K* value. In addition, the too-short central part brings the relatively bulky imidazolium rings deep into the portals to compromise the complex stability by steric hindrance. Nevertheless, in some cases, the length of the central part and bulkiness of the terminal cations cannot fully explain the binding strength. For instance, the **4f** is long enough to allow relatively small ammonium cations to occupy the optimal positions in the portals (*b* = 0.63 Å, see Table 2), but the log *K*_CB6_ is only 2.74. Thus, the bulkiness of the central parts of the guests should be considered.

**Figure 6:**
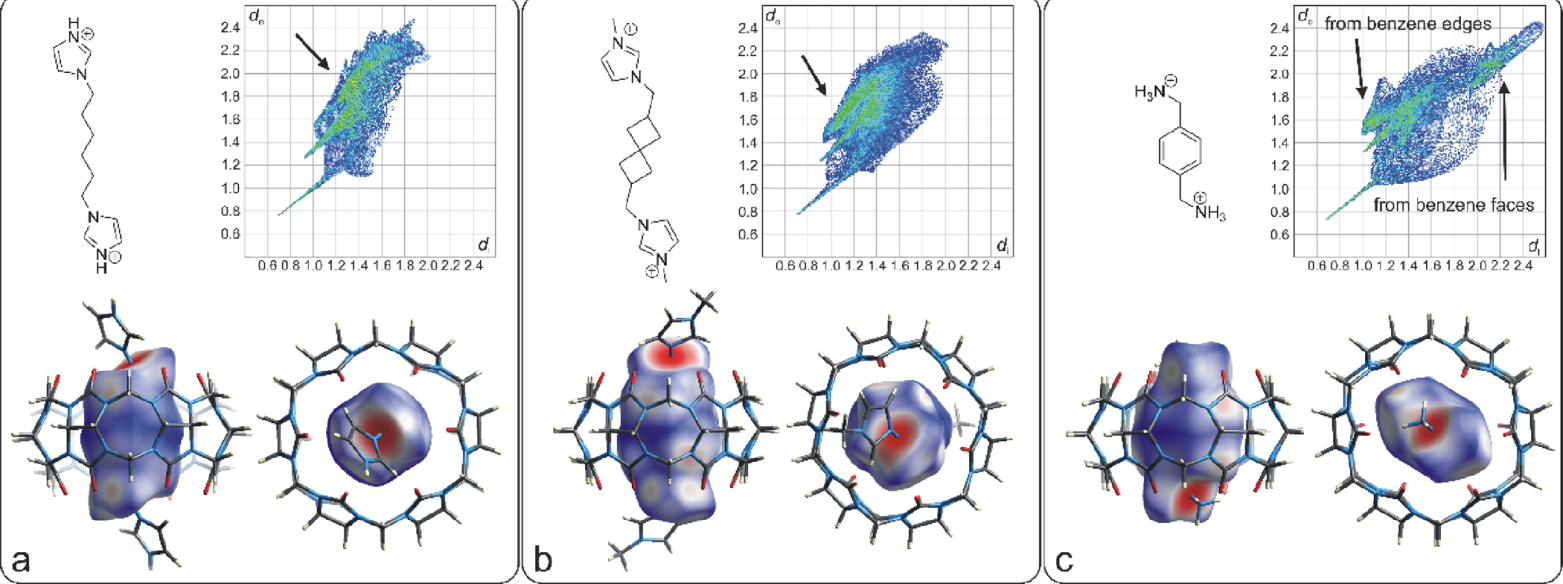
Hirshfeld surfaces for complexes **4e**@CB6 (a), **4a**@CB6 (b), and **4f**@CB6 (c); (the *d*_*i*_ and *d*_*e*_ values are given in Å). The black arrows show the lobes that correspond to the guest contacts with host C atoms and N atoms.

The spatial complementarity of the guest’s central part and cavity interior can be obtained from an analysis of the Hirshfeld surface (HS) inside the cavity.^8,28^ The Hirshfeld surfaces for the complexes of two selected model guests (**4e**, **4f**) and **4a** with CB6 are shown in Figure 6. The Hirshfeld surface is a buildup of points with equal electronic contribution from the inside and outside, where *d*_i_ and *d*_e_ are the distances from the inside and outside contributing atoms to the particular point on the surface. Thus, the *d*_i_/*d*_e_ plot shows the stiffness of the guest inside the cavity. A significant portion of points with high values of *d*_i_ and/or *d*_e_ indicates incompatible shapes, and vice versa. Guest **4f**, with a planar central part of the molecule, is an example of a poorly suited guest for an originally sphere-shaped (actually, somewhat elliptical within the complex; see Table 2) CB6 interior cavity. Figure 6c shows a lobe that reaches *d* values of 2.4 Å, which represent long-distance contacts between C/H atoms of the benzene ring and equatorial C/N atoms of the CB6. Figure 6a,b demonstrates that the spiroheptane skeleton has shorter contacts to the inside cavity walls compared to the hexamethylene linker of guest **4e**. The highest populated *d* values (pointed out by a black arrow in Figure 6b) are related to the contacts of guest **4a** with equatorial CB6 C/N atoms. Note that these values are markedly lower than those of **4e** to indicate closer contacts in the cavity. Consequently, a higher contribution of dispersion forces to the complex stability can be expected for guest **4a**. The qualitative results of the HS analysis and related binding behavior toward CB6 correlate with the simply calculated effective diameters (*d*) of the central parts of the guests, as shown in Table 2. When considering MM2-optimized models, the *p*-xylylene guest, **4f**, with the highest *d* value forms a weak complex with CB6, most likely due to the planar nature of the benzene ring, which allows for CB6 macrocycle shape adaptation. The more symmetric guest, **4b**, with the second-highest *d* value, disables an efficient shape adjustment to hinder the complex formation. Spiroheptane guest **4a** and guests **4c** and **4d** with moderate to small *d* values form relatively stable complexes with CB6.

## Conclusions

As a part of our ongoing interest in novel binding motifs for host–guest systems, we prepared three model guests based on spiro[3.3]heptane (**4a** and **8a**) and bicyclo[1.1.1]pentane (**4b**). Our original motivation was to find an axially disubstituted rigid aliphatic binding motif for CB6 that could be used for the construction of multitopic guests. Therefore, we tested the guests’ binding properties toward other frequently used hosts, i.e., CB7, α-CD, and β-CD. It has been found that **4a**, **8a**, and **4b** form highly stable complexes with CB7 with their respective *K* values of 1.2 × 10^12^, 3.8 × 10^12^, and 1.1 × 10^11^ M^–1^. These values are of the same magnitude as previously published analogous guests derived from 1,4-disubstituted cubane (*K*_CB7_ = 5.96 × 10^11^ M^–1^)^8^ and 1,3-disubstituted adamantane (*K*_CB7_ = 1.64 × 10^11^ M^–1^).^7c^ In the context of a broader set of guests, the stability of the CB7 complexes with our new guests lies between *p*-xylylenediammonium (*K*_CB7_ = 1.80 × 10^9^ M^–1^)^7b^ and 1,4-bis(diammoniomethyl)bicyclo[2.2.2]octane (*K*_CB7_ = 2.00 × 10^14^ M^–1^).^7a^ Indeed, neither of the so far described aliphatic rigid binding motifs forms a stable inclusion complex with CB6. The very first examples are guests **4a** and **8a**, which display a moderate affinity toward CB6 (4.0 × 10^4^ and 1.4 × 10^6^ M^–1^, respectively) in a 50 mM NaCl solution. By comparing the binding behavior of our new guests and the guests with linear aliphatic centerpieces, we demonstrated that bulkier motifs are preferred by cucurbit[*n*]uril macrocycles, as clearly indicated by the *K* values in the case of CB7. However, in the case of a narrow interior cavity (CB6), the binding strength was compromised by the inappropriate length of the centerpiece and the rigidity of the guests, resulting in the steric hindrance of cationic moieties within the macrocycle portals. The respective binding affinities of **4a** (bisimidazolium salt) and **8a** (diammonium salt) toward each of CB6 and CB7 indicate that the nature of the cationic moiety influences the complex stability much more significantly in the case of CB6. Interestingly, the complex formation kinetics is significantly dependent on the nature of the cations in the case of spiro[3.3]heptane guests. Whereas the mixture of CB6 with **4a** needs seconds, the system with **8a** requires hours to reach an equilibrium. Both the examined structural motifs, particularly spiro[3.3]heptane with a thermodynamic selectivity toward CB7/CB6 of 2.5 × 10^7^ for **4a** and 2.8 × 10^6^ for **8a**, represent promising binding sites for the design and construction of multitopic guests for advanced supramolecular devices.

## Experimental Part

All solvents, reagents, and starting compounds were of analytical grade, purchased from commercial sources, and used without further purification if not stated otherwise. Spiro[3.3]heptane dicarboxylic acid **1a** and bicyclo[1.1.1]pentane dicarboxylic acid **1b** were prepared following previously published procedures.^15^ Melting points were measured on a Kofler block. Elemental analyses (C, H, and N) were performed using a Thermo Fisher Scientific Flash EA 1112. NMR spectra were recorded using a Jeol JNM-ECZ400R/S3 spectrometer operating at frequencies of 399.78 MHz (^1^H) and 100.53 MHz (^13^C) and an Avance III Bruker NMR spectrometer operating at frequencies of 401.00 MHz (^1^H) and 100.83 MHz. ^1^H- and ^13^C-NMR chemical shifts were referenced to the signal of the solvent [^1^H: δ(residual DMSO-*d*_5_) = 2.50 ppm, δ(residual HDO) = 4.70 ppm, δ(residual CHCl_3_) = 7.27 ppm; ^13^C: δ(DMSO-*d*_6_) = 39.52 ppm; δ(CDCl_3_) = 77.16 ppm]. The mixing time for ROESY was adjusted to 200 ms for **4a**. Signal multiplicity is indicated by “s” for singlet, “d” for doublet, “m” for multiplet, and “um” for unresolved multiplet. Signal assignment is based on APT, DEPT-135, ^1^H–^1^H-COSY, edited ^1^H–^13^C-HSQC, ^1^H–^13^C-HMBC, and ROESY experiments. The spectra are given in the Supporting Information. IR spectra were collected using an FT-IR spectrometer Alpha (Bruker Optics GmbH Ettlingen, Germany) with a KBr pellets technique. Electrospray mass spectra (ESI-MS) were recorded by using an amaZon X ion-trap mass spectrometer (Bruker Daltonics, Bremen, Germany) equipped with an electrospray ionization source. All of the experiments were conducted in the positive-ion polarity mode. The instrumental conditions used to measure the single imidazolium salts and their mixtures with the host molecules are given in detail in the Supporting Information. Isothermal titration calorimetry measurements were carried out using a VP-ITC MicroCal instrument in H_2_O or 50 mM NaCl at 303 K. The concentrations of the host in the cell and the guest in the microsyringe were approximately 0.15 and 1.50 mM for the determination with CB6 and 2.50 and 25.00 for β-CD, respectively. The raw experimental data were analyzed with the MicroCal ORIGIN software. The heats of dilution were taken into account for each guest. The data were fitted to a theoretical titration curve using the “One Set of Sites” model. If needed, a competitive approach was employed and the concentrations of the host in the cell and the guest in the microsyringe were approximately 0.05 and 0.50 mM for CB7 and CB6, respectively. The *K* values obtained from the competitive titrations were verified using two different concentrations of competitor. All titrations were performed in triplicate. Details for X-ray diffraction measurements can be found in the Supporting Information. CCDC 2171957 (**4a**@CB6) and 2271806 (**4b**) contain the supplementary crystallographic data for this paper. The data can be obtained free of charge from The Cambridge Crystallographic Data Centre via www.ccdc.cam.ac.uk/data_request/cif.

### Bicyclo[1.1.1]pentane-1,3-diyldimethanol (**2b**)

A dry and argon-filled 50 mL three-neck flask was charged with dimethyl bicyclo[1.1.1]pentane-1,3-dicarboxylate^15b−15d^ (**1b**) (500 mg, 2.715 mmol, 1.0 equiv) and THF (10 mL). The clear colorless solution was kept at 0 °C in a water/ice cooling bath, and LiAlH_4_ (309 mg, 8.144 mmol, 3 equiv) was added portion-wise. The cooling was stopped, and the gray suspension was stirred for 3 h at room temperature. Then, it was cooled to 0 °C (water/ice bath) again and a 10% aqueous NaOH (600 μL) followed by water (600 μL) was added dropwise. Cooling was stopped, and the white suspension was stirred for 30 min at room temperature. Then, THF (10 mL) was added, the reaction mixture was filtered, and solids were washed with THF (30 mL). The combined organic phases were dried over MgSO_4_, and the solvent was removed under reduced pressure. Compound **2b** was obtained as a colorless oil (340 mg, 2.653 mmol, 98%) in a purity sufficient for the next reaction.

^1^H NMR (400 MHz, CDCl_3_): δ 3.62 (s, 4H), 1.65 (s, 6H). ^13^C{^1^H} NMR (100 MHz, CDCl_3_): δ 63.4, 47.3, 40.4. IR (KBr): 3338, 2968, 2908, 2869, 1447, 1369, 1316, 1269, 1139, 1098, 1047, 1007, 956, 828, 675, 628 cm^–1^. MS, *m*/*z* (%): 127.1 (30, [M–H^+^]^−^), 124.1 (30), 113.1 (100). HRMS (APCI) *m*/*z*: Calcd for C_7_H_13_O_2_^+^ 129.0910 ([M+H]^+^); found 129.0908. Anal. Calcd for C_7_H_12_O_2_: C 65.60; H 9.44. Found: C 65.82; H 9.51.

### 1,3-Bis(bromomethyl)bicyclo[1.1.1]pentane (**3b**)

A solution of PPh_3_ (1.967 g, 7.500 mmol)) in CH_2_Cl_2_ (5 mL) was added dropwise to a solution of **2b** (340 mg, 2.653 mmol) and CBr_4_ (1.923 g, 5.800 mmol) in CH_2_Cl_2_ (20 mL) at 0 °C. The pale orange reaction mixture was stirred at 0 °C (water/ice cooling bath) for an additional 4 h. Then, it was poured into pentane (60 mL) causing the immediate formation of a white precipitate, and the mixture was stirred for 30 min. Solids were removed by filtration and thoroughly washed with pentane (2 × 30 mL). All organic phases were combined, and solvents were removed under reduced pressure. The yellowish solid residue was thoroughly triturated with an ice-cold pentane (6 × 10 mL), and solids were separated using a centrifuge (3000 rpm, 5 min). The supernatants were combined, and evaporation of the solvent under reduced pressure gave **3b** (590 mg, 2.323 mmol, 88%) as a yellowish oil in a purity sufficient for the next reaction.

^1^H NMR (400 MHz, CDCl_3_): δ 3.47 (s, 4H), 1.73 (s, 6H). ^13^C{^1^H} NMR (100 MHz, CDCl_3_): δ 47.8, 39.0, 33.7. IR (KBr): 2972, 2908, 2873, 1446, 1438, 1429, 1265, 1231, 1201, 1139, 1120, 1091, 1050, 881, 722, 695, 659, 644, 629, 542 cm^–1^. MS, *m*/*z* (%): 94.1 (17), 93.1 (100, [CH_2_Br]^+^), 91.1 (17). HRMS (APCI) *m*/*z*: Calcd for C_6_H_8_Br^+^ 158.9809 ([M–CH_2_Br]^+^); found 158.9810. Anal. Calcd for C_7_H_10_Br_2_: C 33.11; H 3.97. Found: C 33.05; H 3.77.

### 1,3-Bis((1*H*-imidazol-1-yl)methyl)bicyclo[1.1.1]pentane (**5**)

A dry and argon-filled 50 mL three-neck flask was charged with NaH (969 mg, 40.394 mmol, 19 equiv) and DMF (20 mL). Subsequently, imidazole (2.894 g, 45.526 mmol, 20 equiv) was added portion-wise to the reaction mixture at room temperature. (Warning: the NaH/DMF mixture decomposes rapidly under heating.)^29^ The original suspension completely dissolved leaving a clear yellowish solution that was stirred at room temperature for an additional 60 min. Then, it was cooled to 0 °C using the water/ice cooling bath, and the solution of **3b** (540 mg, 2.126 mmol, 1 equiv) in DMF (4 mL) was added dropwise. The clear orange reaction mixture was stirred at a temperature between 0 and 5 °C for an additional 4 h. Subsequently, the mixture was diluted with water (50 mL) and extracted with CH_2_Cl_2_ (4 × 25 mL). The combined organic phases were dried over MgSO_4_, and volatiles were removed under reduced pressure. The residual DMF as well as the excess imidazole was distilled and sublimed using a Kugelrohr distillation apparatus (135 °C, 600 mTorr, 2 h). The column chromatography of the yellow solid residue on the silica gel (CHCl_3_:MeOH, 3:2, v:v) afforded **5** as a white crystalline solid (334 mg, 1.463 mmol, 69%).

Mp 98–101 °C. ^1^H NMR (400 MHz, CDCl_3_): δ 7.47 (s, 2H), 7.05 (s, 2H), 6.82 (s, 2H), 4.01 (s, 4H), 1.60 (s, 6H). ^13^C{^1^H} NMR (100 MHz, CDCl_3_): δ 137.1, 129.5, 119.2, 49.1, 48.6, 39.1. IR (KBr): 3092, 2965, 2930, 2904, 2867, 1510, 1437, 1390, 1358, 1293, 1263, 1237, 1227, 1145, 1111, 1108, 1094, 1074, 1053, 910, 879, 808, 777, 753, 727, 680, 662, 621, 542 cm^–1^. MS, *m*/*z* (%): 229.1 (90, [M+H^+^]^+^), 163.1 (30), 69.0 (100). HRMS (APCI) *m*/*z*: [M+H^+^]^+^ Calcd forC_13_H_17_N_4_^+^ 229.1453; found 229.1454. Anal. Calcd for C_13_H_16_N_4_: C 68.39; H 7.06; N 24.54. Found: C 66.61; H 6.97; N 24.55.

### 1,1′-(Bicyclo[1.1.1]pentane-1,3-diylbis(methylene))bis(3-methyl-1*H*-imidazol-3-ium) Diiodide (**4b**)

CH_3_I (68 μL, 1.095 mmol, 2.5 equiv) was added to the solution of **5** (100 mg, 0.438 mmol, 1.0 equiv) in DMF (5 mL) at room temperature. The flask was wrapped with aluminum foil, and the reaction mixture was stirred at the same temperature for additional 3 days. The progress of the reaction was monitored using ESI–MS. Then, ether was added to the reaction mixture until a white solid precipitated, and the suspension was stirred for an additional 10 min. The solid was collected by filtration, washed with ether (2 × 5 mL), and thoroughly dried under reduced pressure. Compound **4b** was obtained as a white crystalline solid (196 mg, 0.383 mmol, 87%).

Mp 177–180 °C. ^1^H NMR (400 MHz, DMSO-*d*_6_): δ 9.05 (m, 2H), 7.72 (m, 2H), 7.66 (m, 2H), 4.35 (s, 4H), 3.86 (s, 6H), 1.59 (s, 6H). ^13^C{^1^H} NMR (100 MHz, DMSO-*d*_6_): δ 136.3, 123.7, 122.5, 49.4, 45.5, 38.0, 35.9. IR (KBr): 3161, 3135, 3080, 3067, 3030, 2983, 2972, 2959, 2911, 2870, 1575, 1560, 1553, 1466, 1488, 1438, 1426, 1381, 1357, 1337, 1295, 1261, 1225, 1162, 1136, 1093, 1074, 1059, 1016, 952, 857, 780, 762, 734, 688, 641, 622, 549 cm^–1^. ESI–MS (pos) *m*/*z* (%): 83.0 [IM+H^+^]^+^ (3), 128.9 [M^2+^]^2+^ (100), 385.0 [M^2+^+I^–^]^+^ (56). HRMS (ESI) *m*/*z*: [M+H]^+^ Calcd for C_15_H_22_N_4_I^+^385.0884; found 385.0878. Anal. Calcd for C_15_H_22_I_2_N_4_: C 35.18; H, 4.33; N 10.94. Found: C 34.96; H 4.14; N 10.69.

### Spiro[3.3]heptane-2,6-diyldimethanol (**2a**)

The compound **2a** was prepared according to a modified previously published procedure.^15a^ A dry and argon-filled 100 mL round-bottom flask was charged with freshly distilled diethyl ether (60 mL) and LiAlH_4_ (250 mg, 5.590 mmol). The suspension was kept at 0 °C in the water/ice cooling bath, and 500 mg (2.715 mmol) of acid **1a** was added in small portions. Subsequently, the mixture was refluxed for 9 h using an oil bath. The white dispersion was cooled to 0 °C, and the remaining LiAlH_4_ was destroyed by 1 mL of H_2_O. The resulting white solid was filtered off and extracted with DCM using a Soxhlet extractor. The solvent was removed under reduced pressure to yield the diol **2a** (312 mg, 61%) as a colorless oil in a purity sufficient for the next reaction.

^1^H NMR (400 MHz, DMSO-*d*_6_): δ 4.33 (t, 2H, *J* = 5.2 Hz), 3.29 (um, obscured by H_2_O signal), 2.18 (septet, 2H, *J* = 7.2 Hz), 1.99 (um, 2H), 1.86 (um, 2H), 1.69 (um, 2H), 1.64 (um, 2H). ^13^C{^1^H} NMR (100 MHz, DMSO-*d*_6_): δ 65.4, 37.8, 37.5, 36.0, 31.6.

### 2,6-Bis(bromomethyl)spiro[3.3]heptane (**3a**)

Alcohol **2a** (0.312 g; 2.01 mmol) and CBr_4_ (1.868 g, 5.63 mmol) were dissolved in dry DCM (15 mL) under an argon atmosphere, and the solution was cooled to 0 °C with an ice/water bath. Subsequently, Ph_3_P (1.899 g, 7.24 mmol) was added in small portions over 20 min. The reaction mixture was stirred at 0 °C for 3 h and then poured into cold pentane with vigorous stirring. The pentane solution was stirred for 20 min at 0 °C and then filtered. The filtrate was evaporated to dryness. The solid residue was triturated with cold pentane and centrifuged five times. The pentane fractions were collected, and the solvent was removed under vacuum to give compound **3a** as a colorless crystalline solid (0.255 g, 45%).

^1^H NMR (400 MHz, CDCl_3_): δ 3.38 (d, 4H, *J* = 7.6 Hz), 2.59 (um, 2H), 2.24 (um, 2H), 2.10 (um, 2H), 1.82–1.70 (um, 4H). ^13^C{^1^H} NMR (100 MHz, CDCl_3_): δ 40.4, 39.9, 39.2, 33.9, 32.3. IR (KBr): 630, 673, 1047, 1162, 1213, 1243, 1281, 2844, 2918, 2954 cm^–1^. EI-MS *m*/*z* (%): 40 (13), 41 (58), 51 (8), 53 (27), 55 (10), 65 (11), 67 (31), 77 (20), 78 (5), 79 (100), 80 (80), 81 (80), 82 (9), 91 (15), 92 (5), 93 (33), 107 (11), 121 (26), 160 (5).

### 2,6-Bis(3-methylimidazolio)spiro[3.3]heptane Dibromide (**4a**)

Bromide **3a** (0.255 g; 0.91 mmol) and 1-methylimidazole (0.223 g; 2.72 mmol) were dissolved in dry acetonitrile (5 mL) under an argon atmosphere and refluxed for 7 days using an oil bath. Afterward, the reaction mixture was cooled down and freshly distilled diethyl ether was added at room temperature. The precipitate was centrifuged, washed with diethyl ether (3 × 10 mL), and dried in a vacuum to yield a colorless crystalline solid (0.320 g; 79%).

Mp 151–153 °C. ^1^H NMR (400 MHz, DMSO-*d*_*6*_): δ 9.12 (s, 2H), 7.71 (um, 4H), 4.16 (d, 4H, *J* = 7.6 Hz), 3.85 (s, 6H), 2.59 (quintet, 2H), 2.11 (um, 2H), 2.00 (um, 2H), 1.75–1.85 (um, 4H). ^13^C{^1^H} NMR (100 MHz, DMSO-*d*_*6*_): δ 136.2, 123.5, 122.2, 53.4, 37.7, 37.2, 35.7, 35.3, 29.5. IR (KBr): 490, 626, 668, 686, 731, 794, 870, 1174, 1237, 1285, 1329, 1371, 1428, 1456, 1563, 1576, 1626, 2850, 2950, 3065, 3142, 3452 cm^–1^. ESI–MS (pos) *m*/*z* (%): 83.0 [IM+H^+^]^+^ (3), 142.9 [M^2+^]^2+^ (100), 365.1 [M^2+^+^79^Br^–^]^+^ (10), 367.1 [M^2+^+^81^Br^–^]^+^ (11). Anal. Calcd for C_17_H_26_N_4_Br_2_·0.56 H_2_O: C 44.75; H 5.99; N 12.28. Found: C 44.34; H 6.16; N 12.26.

### General Procedure for the Synthesis of Compounds **4c** and **4d**

Compounds **4c** and **4d** were prepared following the previously described procedure.^30^ The commercial α,ω-dibromoalkane and 1-methylimidazole were dissolved in dry acetonitrile, the flask was placed into an oil bath, and the reaction mixture was refluxed for 8–40 h under an argon atmosphere until the starting material was completely consumed. Subsequently, the reaction mixture was cooled to room temperature while a colorless precipitate appeared. The heterogeneous mixture was concentrated under vacuum, anhydrous diethyl ether was added, and the solid precipitated was filtered with suction, washed with diethyl ether, and dried under vacuum.

### 1,6-Bis(3-methylimidazolio)hexane Dibromide (**4c**)

The title compound was prepared from the following materials: 1,6-dibromohexane (200 mg; 0.82 mmol), 1-methylimidazole (270 mg; 3.28 mmol), and dry acetonitrile (1.7 mL). Pure product **4c** was obtained as a colorless solid with a yield of 265 mg (79%).

Mp 153–156 °C. ^1^H NMR (400 MHz, DMSO-*d*_*6*_): δ9.17 (s, 2H), 7.77 (t, *J* = 1.6 Hz, 2H), 7.70 (t, *J* = 1.6 Hz, 2H), 4.15 (t, *J* = 6.8 Hz, 4H), 3.85 (s, 6H), 1.74–1.81 (m, 4H), 1.24–1.28 (m, 4H). ^13^C{^1^H} NMR (100 MHz, DMSO-*d*_*6*_): δ 136.4, 123.5, 122.2, 48.6, 35.7, 29.0, 24.8. IR (KBr): 3436, 3372, 3105, 3077, 2935, 2863, 1576, 1566, 1165, 786, 574 cm^–1^. ESI–MS (pos) *m*/*z* (%): 83.0 [IM+H^+^]^+^ (5), 123.9 [M^2+^]^2+^ (100), 165.0 [M^2+^–83^+^]^+^ (7), 327.0 [M^2+^+^79^Br^–^]^+^ (14), 329.0 [M^2+^+^81^Br^–^]^+^ (14).

### 1,4-Bis(3-methylimidazolio)butane Dibromide (**4d**)

The title compound was prepared from the following materials: 1,4-dibromobutane (250 mg; 1.16 mmol), 1-methylimidazole (381 mg; 4.64 mmol), and dry acetonitrile (2.4 mL). The pure product **4d** was obtained as a colorless solid with a yield of 331 mg (75%).

Mp 148–152 °C. ^1^H NMR (400 MHz,DMSO-*d*_*6*_): δ 9.20 (s, 2H), 7.78 (t, *J* = 1.6 Hz, 2H), 7.72 (t, *J* = 1.6 Hz, 2H), 4.22 (t, *J* = 5.6 Hz, 4H), 3.85 (s, 6H), 1.76–1.80 (m, 4H). ^13^C{^1^H} NMR (100 MHz, DMSO-*d*_*6*_): δ 136.6, 123.6, 122.2, 47.9, 35.8, 26.0. IR (KBr): 3456, 3406, 3074, 1632, 1578, 1562, 1169, 1156, 856, 790, 625 cm^–1^. ESI–MS (pos) *m*/*z* (%): 109.9 [M^2+^]^2+^ (100), 299.0 [M^2+^+^79^Br^–^]^+^ (13), 301.0 [M^2+^+^81^Br^–^]^+^ (13).

### Spiro[3.3]heptane-2,6-dicarboxamide (**6a**)

A dispersion of Fecht acid **1a** (600 mg, 3.258 mmol) in thionyl chloride (40 mL) was heated by using an oil bath until the acid was completely dissolved. The resulting solution was further refluxed under an argon atmosphere for 5 h. Thionyl chloride was distilled off, and the remaining traces of SOCl_2_ were removed by a vigorous stream of dry nitrogen to obtain dichloride as a pale yellow oil, which was used in further steps without purification. The dichloride was cooled below 10 °C using an ice bath and slowly added dropwise into 28% ammonia in water (15 mL) under vigorous stirring at 10 °C. The mixture was stirred for an additional 3 h at room temperature. The solid was collected by filtration, washed with water (2 × 5 mL), and thoroughly dried under reduced pressure. Compound **6a** was obtained as a white solid (444 mg, 75%).

Mp 248–250 °C. ^1^H NMR (400 MHz, DMSO-*d*_6_): δ 7.05 (s, 2H), 6.59 (s, 2H), 2.78 (p, *J* = 8.4 Hz, 2H), 2.09 (m, 4H), 1.94 (m, 4H). ^13^C{^1^H} NMR (100 MHz, DMSO-*d*_6_): δ 176.3, 38.1, 37.3, 36.0, 33.6. IR (KBr): 3384, 3194, 2965, 2946, 2927, 1652, 1453, 1421, 1294, 1241, 1130, 1100, 810, 793, 722, 690, 646 cm^–1^. EI–MS: 112 (5), 111 (81), 110 (65), 96 (33), 95 (8), 94 (26), 93 (21), 92 (12), 91 (12), 83 (24), 82 (9), 81 (8), 79 (17), 78 (9), 77 (16), 72 (90), 69 (5), 68 (31), 67 (50), 66 (57), 65 (33), 63 (5), 59 (18), 57 (7), 55 (65), 54 (40), 53 (34), 52 (21), 51 (21), 50 (8), 44 (100), 43 (14), 42 (13), 41 (72), 40 (49) *m*/*z* (%). HRMS (ESI) *m*/*z*: [M+H^+^]^+^ Calcd for C_9_H_15_O_2_N_2_^+^ 183.1128; found 183.1128.

### Spiro[3.3]heptane-2,6-dicarbonitrile (**7a**)

Diamide **6a** (120 mg, 0.659 mmol) was heated under reflux for 1.5 h in a mixture of benzene (1.5 mL) and phosphoryl chloride (1 mL). During the reflux, the amide completely dissolved, giving a clear solution. The reaction mixture was then slowly poured into crushed ice (10 g) with stirring, and benzene (20 mL) was added. The benzene layer was separated, and the water phase was extracted with benzene (3 × 20 mL). The collected benzene portions were washed with brine (3 × 30 mL), dried over sodium sulfate, and evaporated to dryness under reduced pressure. Compound **7a** was obtained as a brown oil (92 mg, 96%) in purity sufficient for further steps.

^1^H NMR (400 MHz, CDCl_3_): δ 2.99 (p, *J* = 8.0 Hz, 2H), 2.48 (m, 8H). ^13^C{^1^H} NMR (100 MHz, CDCl_3_): δ 121.6, 39.7, 38.7, 38.6, 17.3. IR (KBr): 3442, 2990, 2945, 2858, 2235, 1639, 1428, 1288, 1205, 1153, 1119, 1075, 1030, 555 cm^–1^. EI–MS: 93 (66), 92 (16), 79 (6), 67 (12), 66 (100), 65 (19), 64 (5), 54 (56), 53 (22), 51 (17), 51 (13), 50 (6), 41 (17), 40 (54) *m*/*z* (%).

### Spiro[3.3]heptane-2,6-diyldimethylammonium Dichloride (**8a**)

A solution of **7a** (160 mg, 1.095 mmol) in dry propan-1-ol (5 mL) was warmed to 80 °C in an oil bath under a nitrogen atmosphere. Metallic sodium (638 mg, 27.740 mmol, 25 equiv) was added in 10 portions over 1 h, and the reaction mixture was heated for an additional 30 min. The progress of the reaction was monitored by NMR. The mixture was then cooled to room temperature, diluted with 30 mL of water, and extracted with dichloromethane (3 × 30 mL). The combined organic portions were washed sequentially with water (5 × 30 mL) and brine (3 × 30 mL) and dried over Na_2_SO_4_. The solvent was removed under reduced pressure using a rotary evaporator to obtain a crude diamine as a colorless oil. This oil was dissolved in anhydrous diethyl ether (10 mL) and a saturated solution of HCl in diethyl ether (2 mL) was added. The resulting suspension was vigorously stirred for 30 min at 0 °C. The precipitate was collected via filtration, and the solid was washed with diethyl ether (5 × 30 mL) and dried under vacuum to obtain compound **8a** as a colorless solid (99 mg, 40%).

Mp 249–252 °C (decomp). ^1^H NMR (400 MHz, D_2_O): δ 3.06 (d, *J* = 7.6 Hz, 4H), 2.54 (m, 2H), 2.35 (m, 2H), 2.19 (m, 2H), 1.87 (m, 4H). ^13^C{^1^H} NMR (100 MHz, D_2_O): δ44.7, 38.0, 37.5, 35.7, 27.3. IR (KBr): 3387, 2954, 2906, 2749, 2642, 2546, 2034, 1606, 1509, 1465, 1397, 965, 458 cm^–1^. ESI-MS (pos) *m*/*z* (%): 155.0 [M+H^+^]^+^ (100). HRMS (ESI) *m*/*z*: [M+H^+^]^+^ Calcd for C_9_H_20_N_2_^2+^ 78.0808; found 78.0808.

## Data Availability

The data underlying this study are available in the published article and its Supporting Information.

## Supporting Information Available

Experimental and computational details, compound characterization data, NMR spectra, mass spectra, ITC data, and crystallographic data (PDF)
