# Supplementary material for: Two Squares in a Barrel: An Axially Disubstituted Conformationally Rigid Aliphatic Binding Motif for Cucurbit[6]uril
Source: J Org Chem. 2023 Oct 26;88(22):15615–25. doi: 10.1021/acs.joc.3c01556 (PMC10661032; doi:10.1021/acs.joc.3c01556)
Supplement: Supplementary file 1 — jo3c01556_si_001.pdf [file jo3c01556_si_001.pdf]

Two squares in a barrel: Axially disubstituted conformationally rigid aliphatic binding motif for cucurbit[6]uril

Kristýna Jelínková,[a,b] Aneta Závodná,[a] Jiří Kaleta,[b] Petr Janovský,[a] Filip Zatloukal,[a] Marek Nečas,[c] Zdeňka Prucková,[a] Lenka Dastychová,[a] Michal Rouchal,[a] Robert Vícha[a,*]

[a] Department of Chemistry, Faculty of Technology, Tomas Bata University in Zlín, Vavrečkova 5669, 760 01 Zlín, Czech Republic

[b] Institute of Organic Chemistry and Biochemistry of the Czech Academy of Sciences, Flemingovo náměstí 2, 16000 Praha, Czech Republic

[c] Department of Chemistry, Faculty of Science, Masaryk University, Kotlářská 2, 602 00 Brno, Czech Republic

[*] corresponding author: rvicha@utb.cz

Table of Contents

General information S2

NMR spectra of intermediates and the final guests **4a**–**4d** and **8a** S3

NMR titration data S28

Mass spectra of the guests and complexes S40

Thermodynamic data S51

Single-crystal X-ray diffraction data S58

General information:

Electrospray mass spectra (ESI-MS) were recorded using an amaZon X ion-trap mass spectrometer (Bruker Daltonics, Bremen, Germany) equipped with an electrospray ionisation source. All the experiments were conducted in the positive-ion polarity mode. The instrumental conditions used to measure the single imidazolium salts and their mixtures with the host molecules were different; therefore, they are described separately. Single guests: Individual samples (with concentrations of 0.5 $\mu g \cdot cm^{-3}$) were infused into the ESI source in methanol:water (1:1, *v*:*v*) solutions using a syringe pump with a constant flow rate of 3 $\mu l \cdot min^{-1}$. The other instrumental conditions were as follows: an electrospray voltage of −4.2 kV, a capillary exit voltage of 140 V, a drying gas temperature of 220 °C, a drying gas flow rate of 6.0 $dm^3 \cdot min^{-1}$, and a nebulizer pressure of 55.16 kPa. Host–guest complexes: An aqueous solution of the guest (12.5 µM) and the equimolar amount of the corresponding host was infused into the ESI source at a constant flow rate of 3 $\mu l \cdot min^{-1}$. The other instrumental conditions were as follows: an electrospray voltage of −4.0 kV, a capillary exit voltage of 140 V up to −40 V, a drying gas temperature of 300 °C, a drying gas flow rate of 6.0 $dm^3 \cdot min^{-1}$, and a nebulizer pressure of 206.84 kPa. Nitrogen was used as both the nebulizing and drying gas for all of the experiments. Tandem mass spectra were collected using CID with He as the collision gas after the isolation of the required ions.

To describe the geometrical parameters of the portals of the cucurbiturils, we calculated **ellipticity** *e* according to the formula: $e = \frac{r_{min}}{r_{max}}$ where r_{min} and r_{max} are semi-minor and semi-major axis, respectively. The ellipse was fitted to the portal O-atoms coordinates using MicroCal Origin. Original 3D coordinates (obtained from X-ray diffraction or computational optimisation) were converted to the orthogonal Cartesian coordinates if needed. A mean plane *P* through portal O-atoms was calculated using Diamond software and O-atoms were projected to this plane. Coordinates were recalculated to set the centre of gravity of the O-atoms to [0,0,0] and the plane *P* was turned to become coplanar with the xy plane. Thus, a set of 2D coordinates [x,z,0] representing original O-atoms mutual positions was obtained. Since the MicroCal Origin can fit only an ellipse aligned to x- and y-axis (non-tilted ellipse), we fitted an ellipse to several orientations of the original set of 2D points turned stepwise by 1° to reach the maximal adjusted R-squared parameter. We analysed each symmetrically non-related portal within the structure to report averaged values of geometrical parameters if applicable. In contrast to the above-mentioned computations based on atomic coordinates, calculating final portal parameters (diameter and area), we took into account the Van der Waals radius of the O-atoms (1.52 Å) obtaining reduced effective portal cross-section.

NMR spectra of intermediates and the final guests 4a–4d and 8a

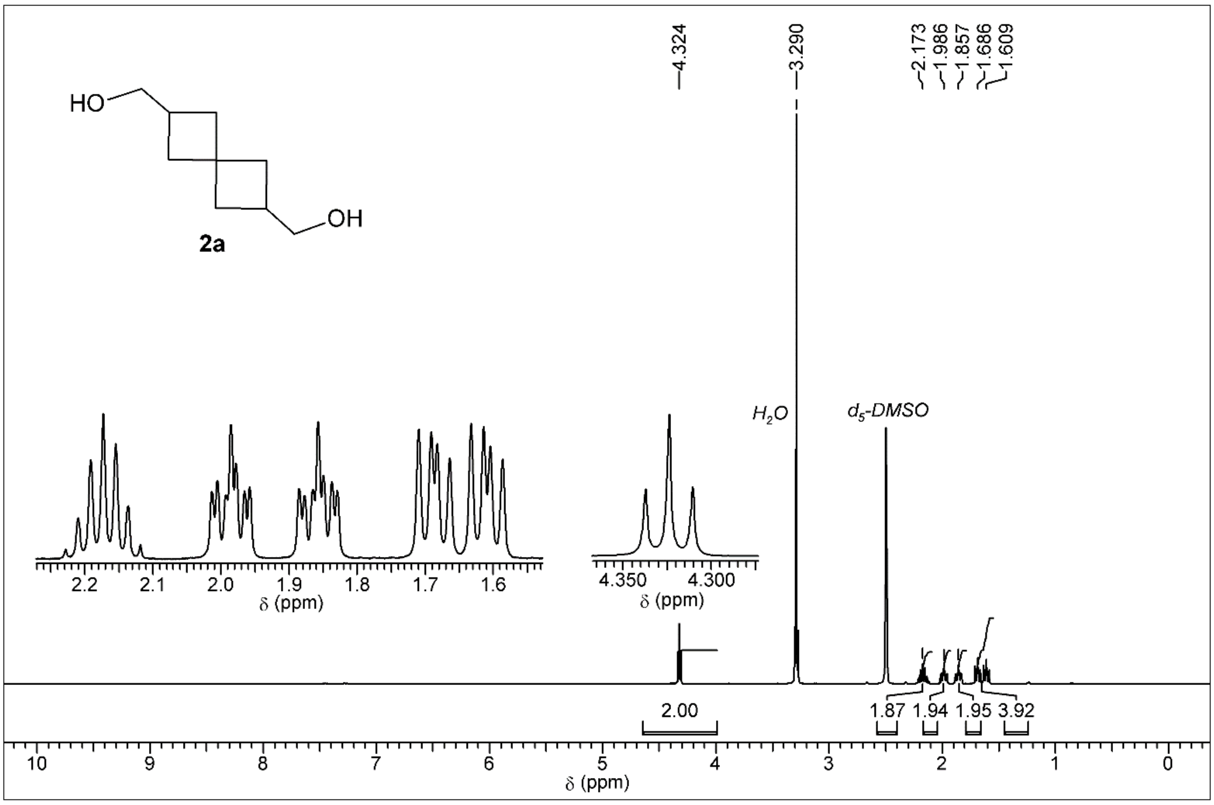

Figure S1 The ^{1}H NMR (d$_6$-DMSO, 400 MHz) spectrum of compound **2a**.

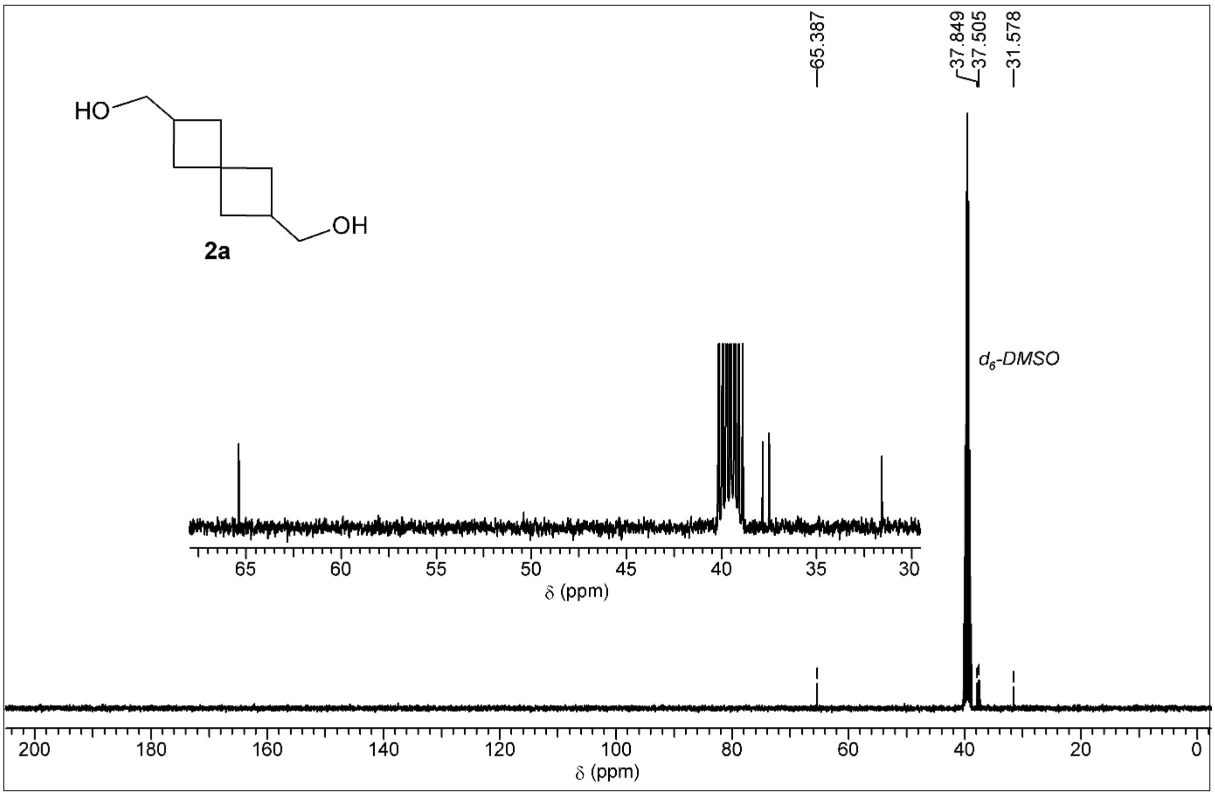

Figure S2 The ^{13}C{^{1}H} NMR (d_6-DMSO, 101 MHz) spectrum of compound **2a**.

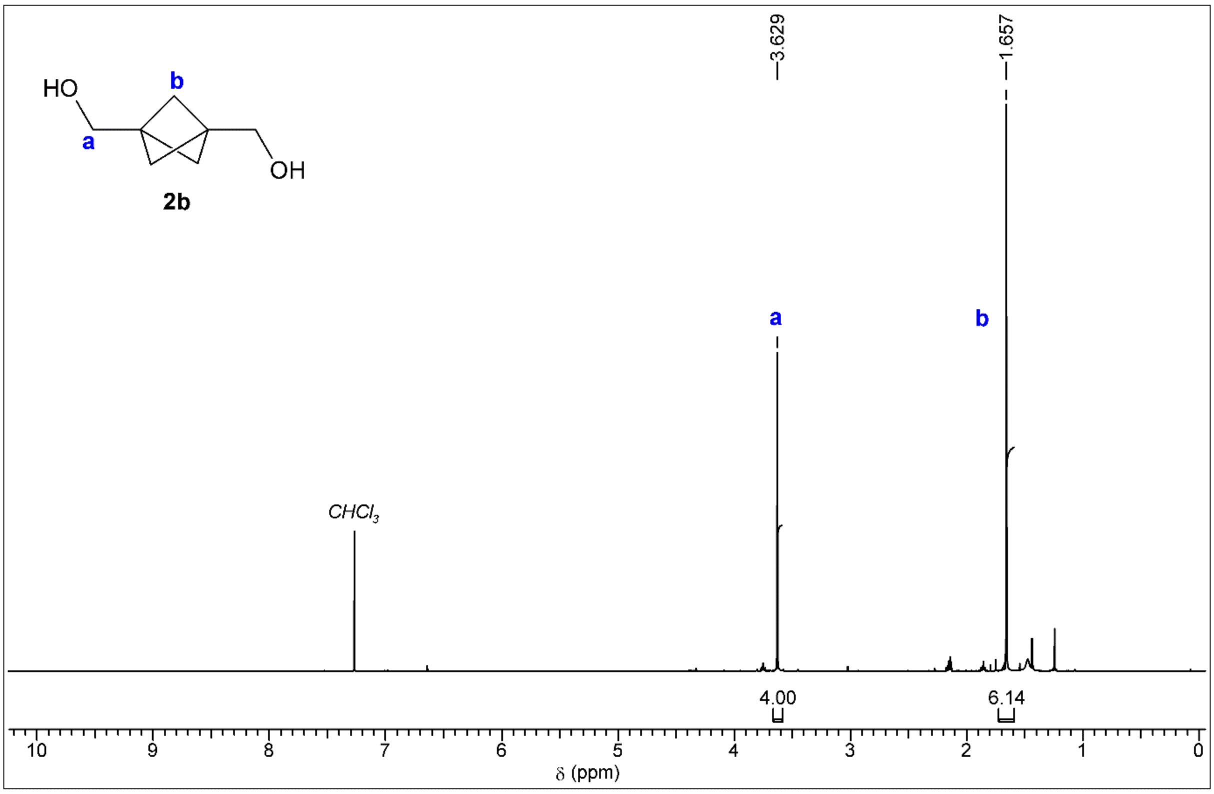

Figure S3 The ¹H NMR ($CDCl_3$, 401 MHz) spectrum of compound **2b**.

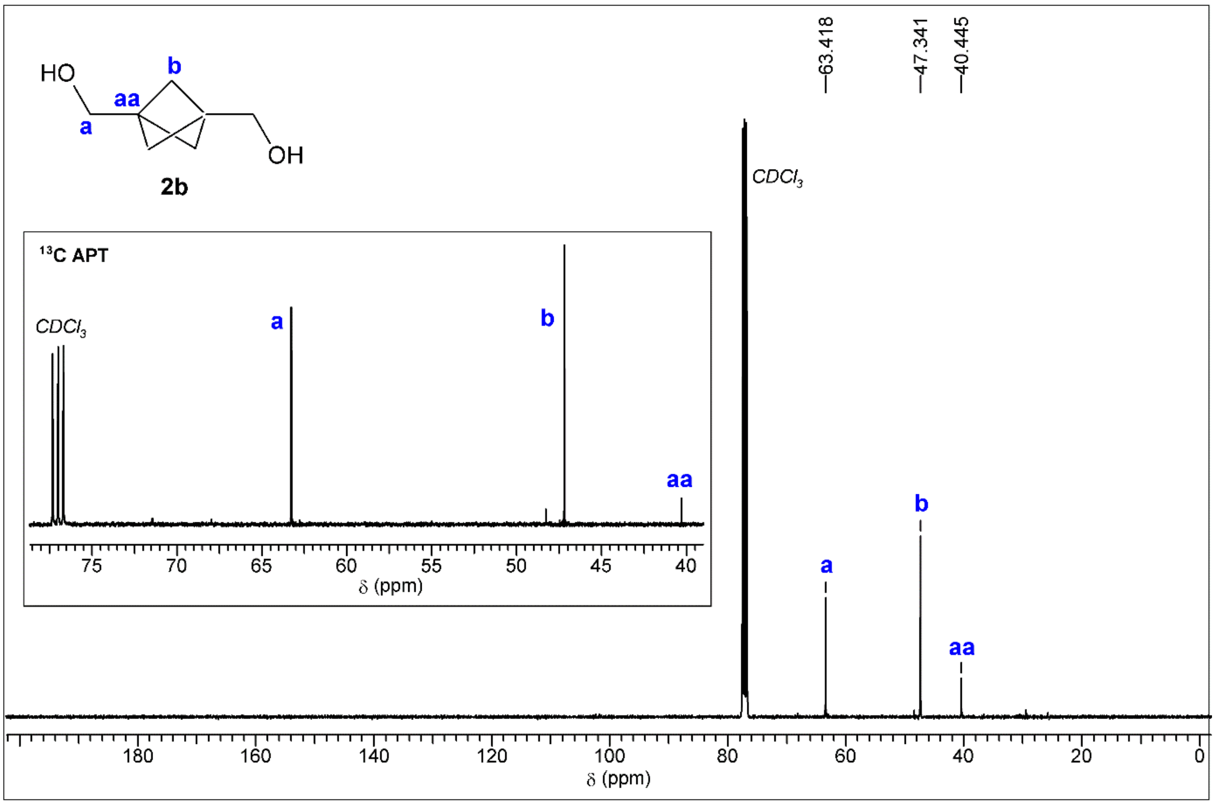

Figure S4 The $^{13}C\{^1H\}$ and ^{13}C APT NMR ($CDCl_3$,101 MHz) spectrum of compound **2b**.

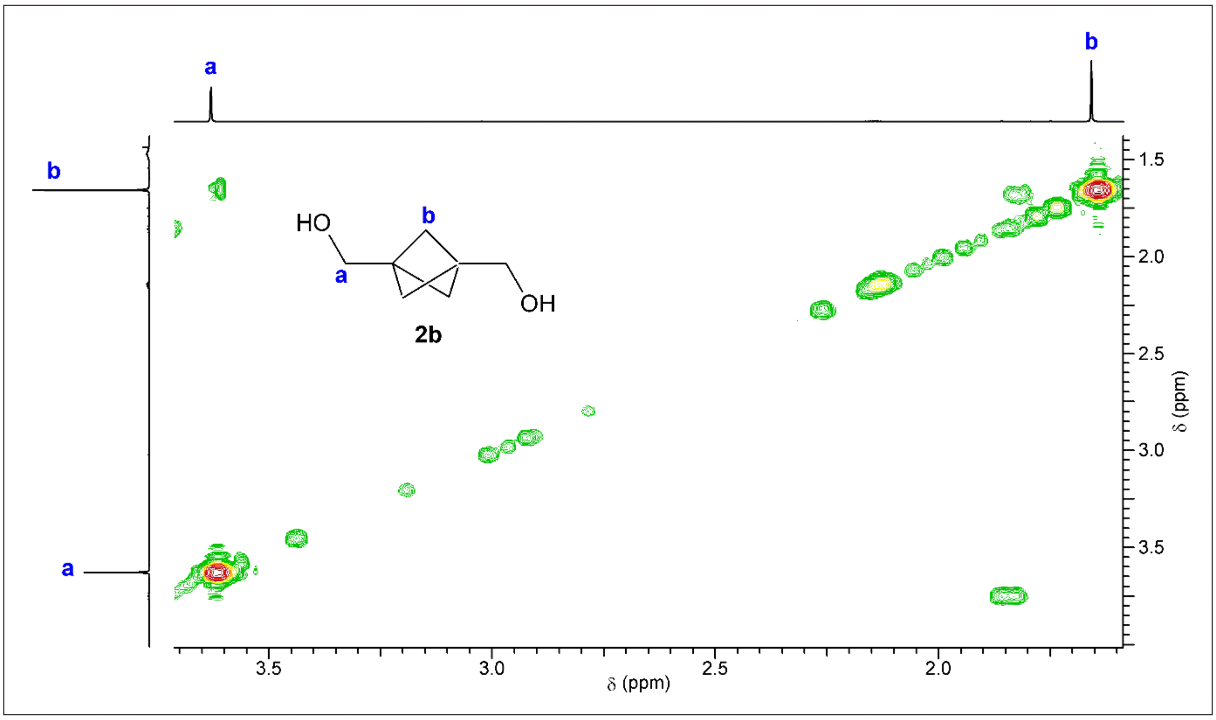

Figure S5 The ^{1}H–^{1}H COSY NMR ($CDCl_3$) spectrum of compound **2b**.

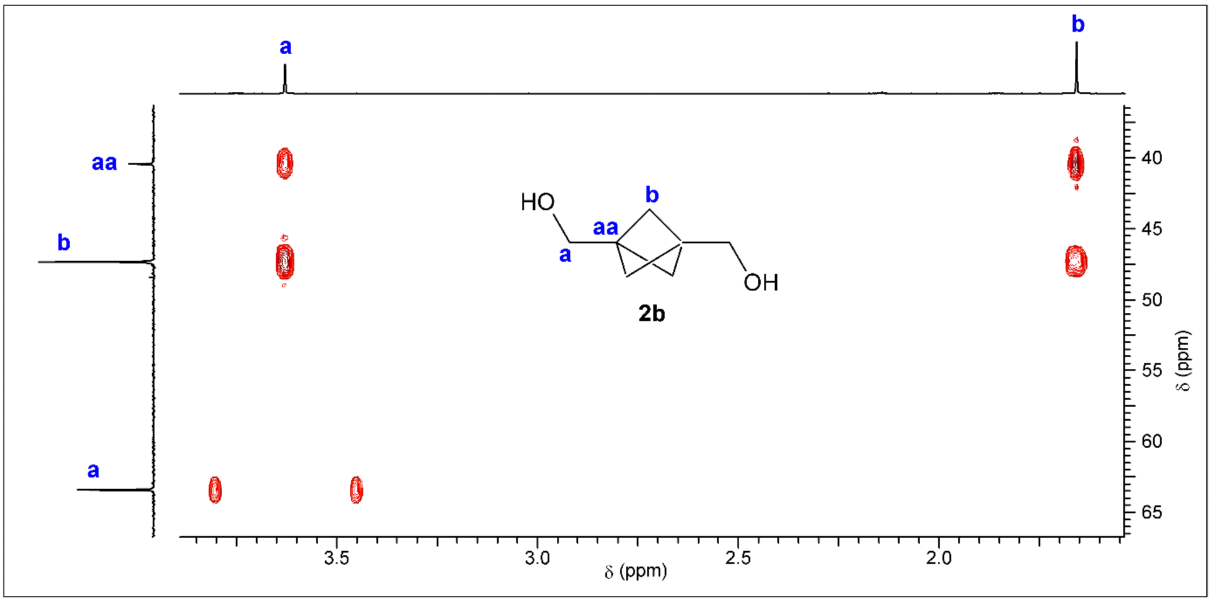

Figure S6 The ^{1}H–^{13}C HMBC NMR ($CDCl_3$) spectrum of compound **2b**.

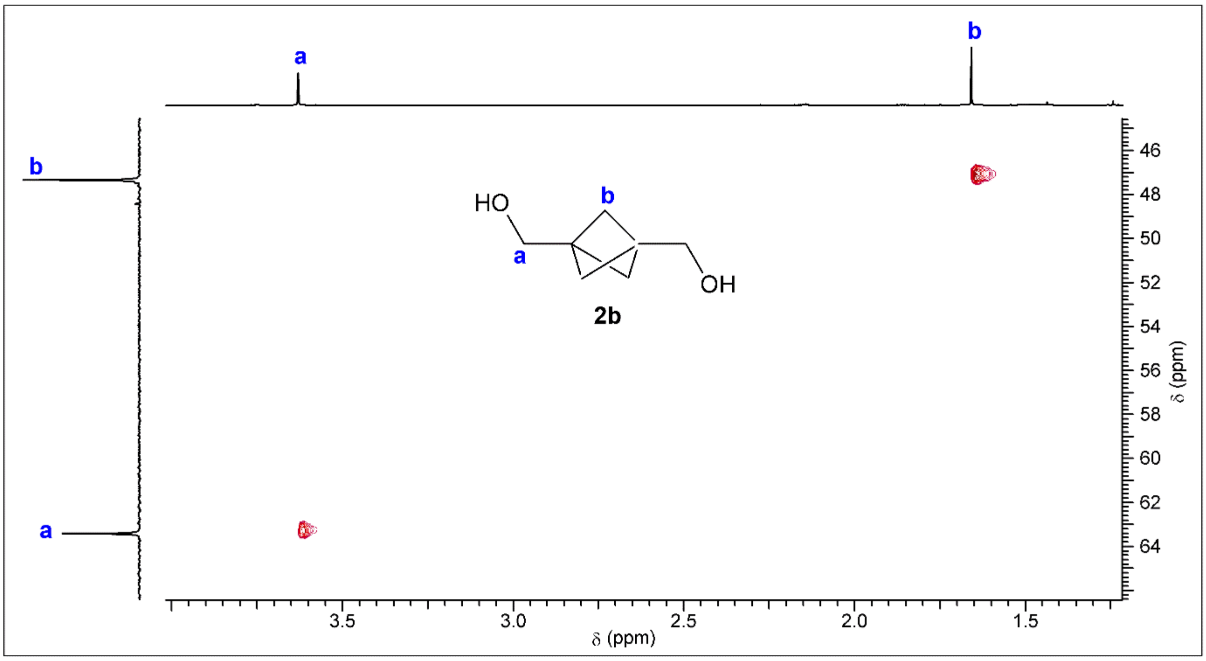

Figure S7 The ^{1}H–^{13}C HSQC NMR ($CDCl_3$) spectrum of compound **2b**.

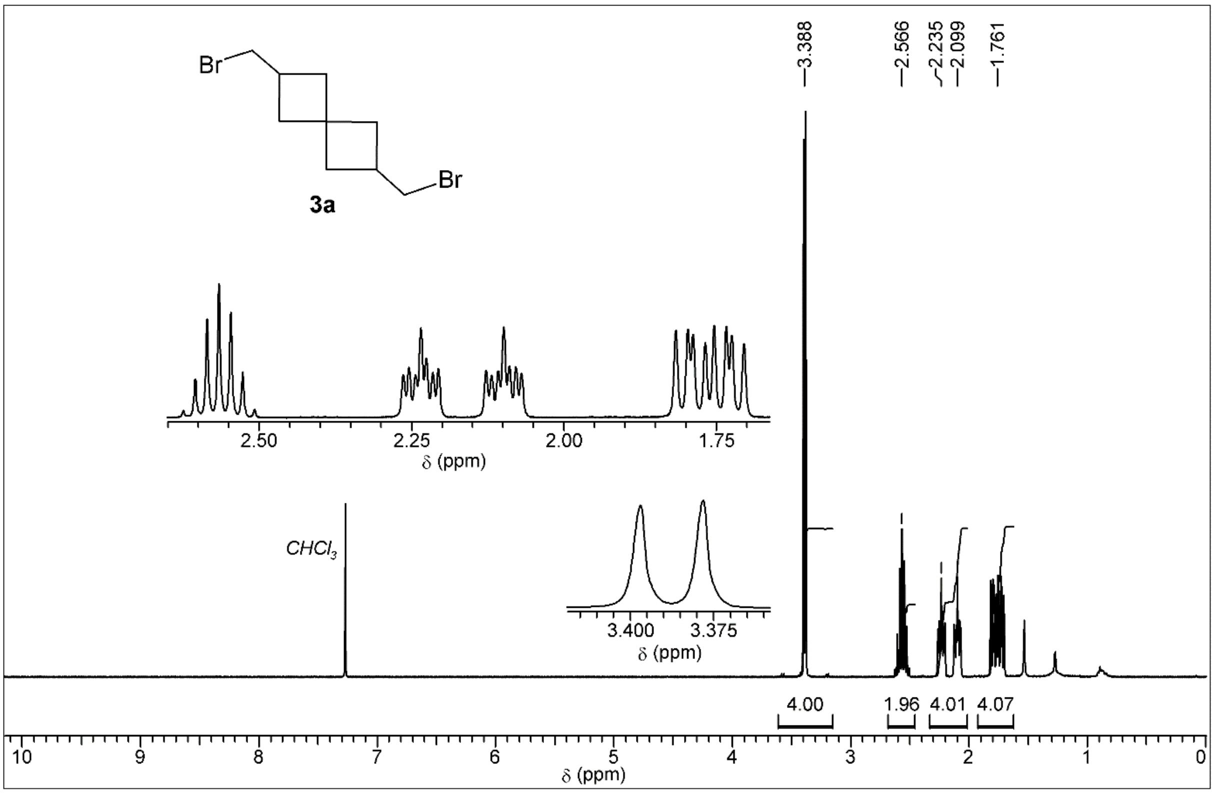

Figure S8 The ^{1}H NMR ($CDCl_3$, 400 MHz) spectrum of compound **3a**.

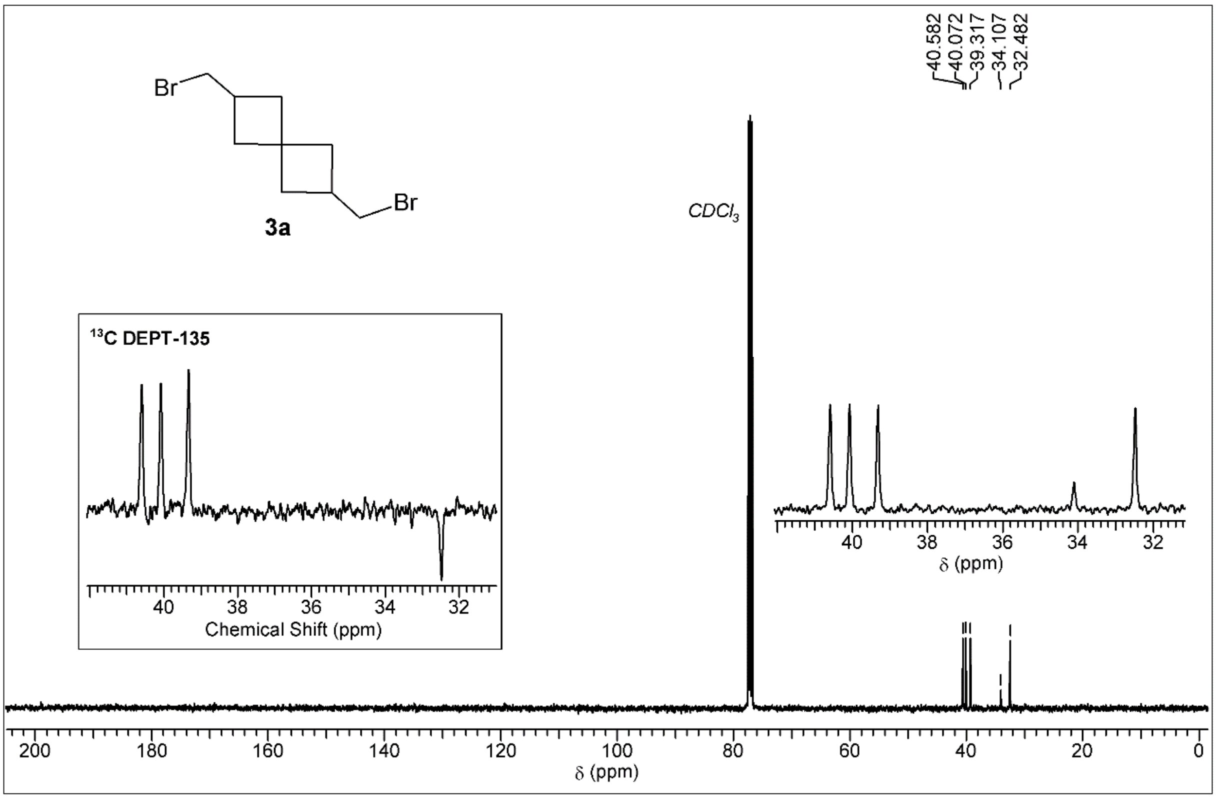

Figure S9 The $^{13}C\{^1H\}$ and ^{13}C DEPT-135 NMR ($CDCl_3$, 101 MHz) spectrum of compound **3a**.

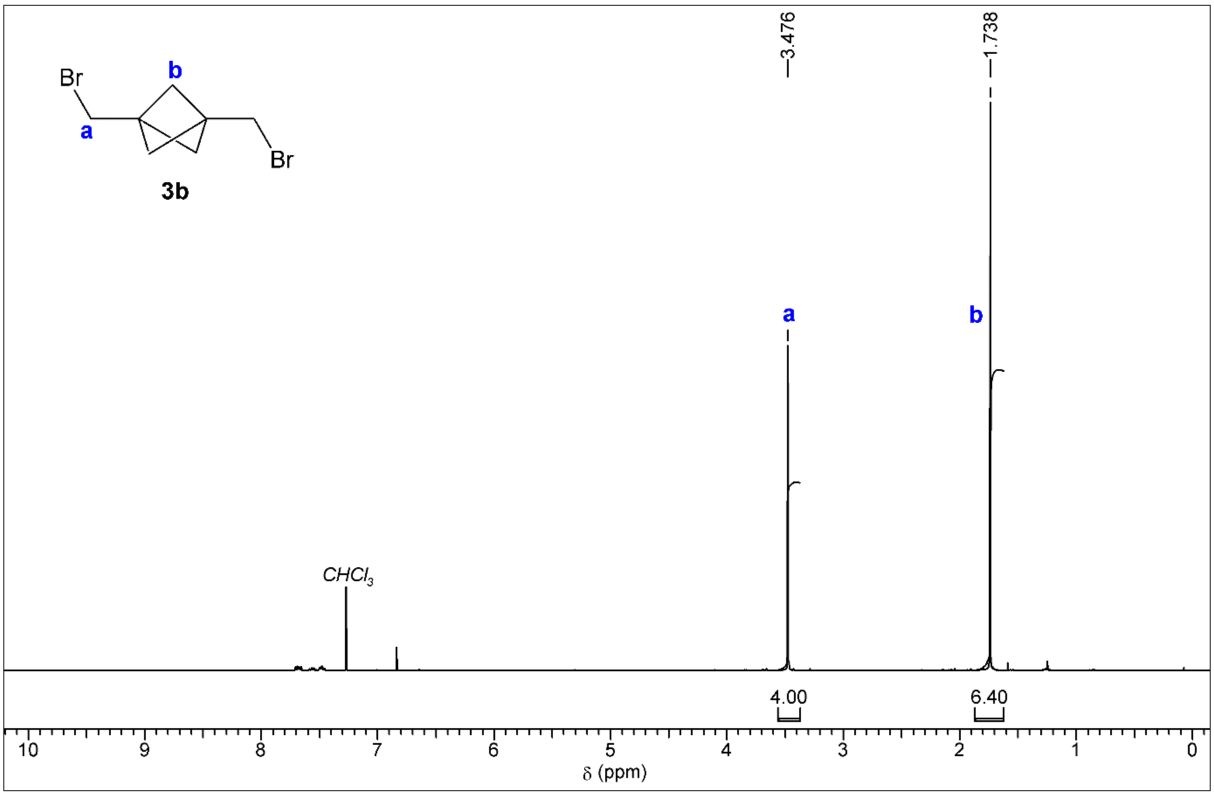

Figure S10 The ^1H NMR ($CDCl_3$, 401 MHz) spectrum of compound **3b**.

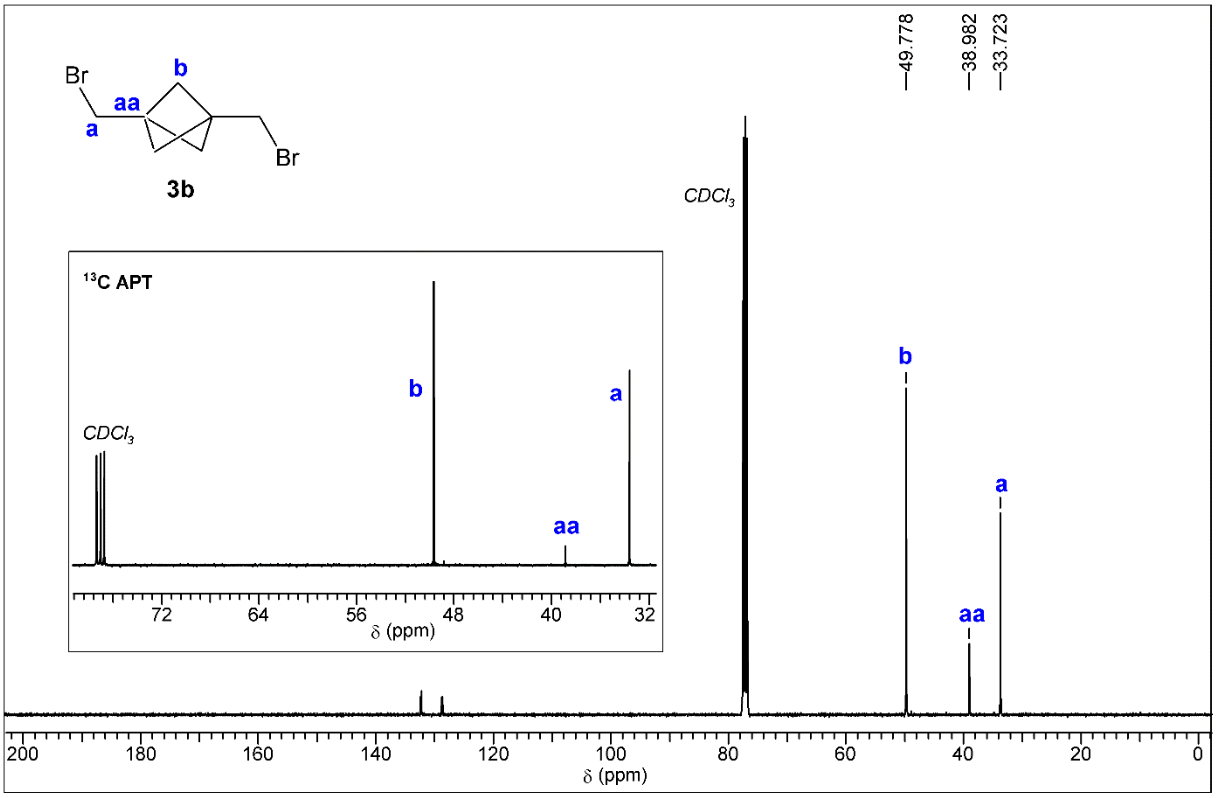

Figure S11 The $^{13}C\{^{1}H\}$ NMR and ^{13}C APT NMR ($CDCl_3$, 101 MHz) spectrum of compound **3b**.

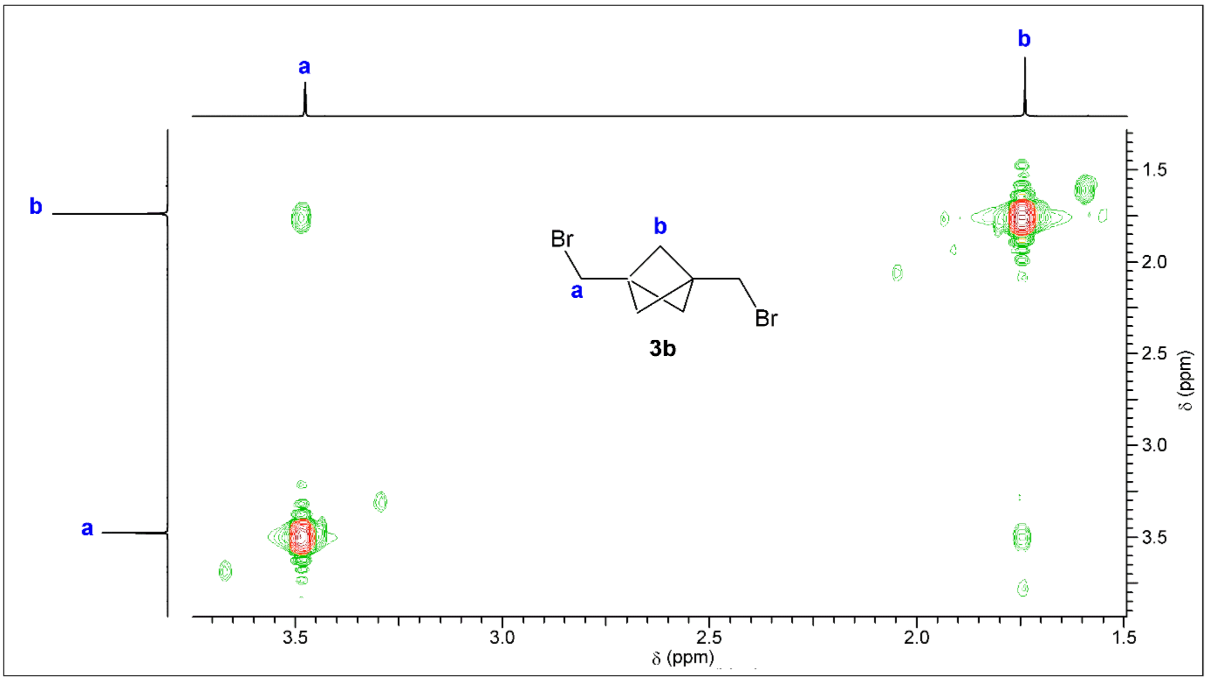

Figure S12 The ^{1}H–^{1}H COSY NMR ($CDCl_3$) spectrum of compound **3b**.

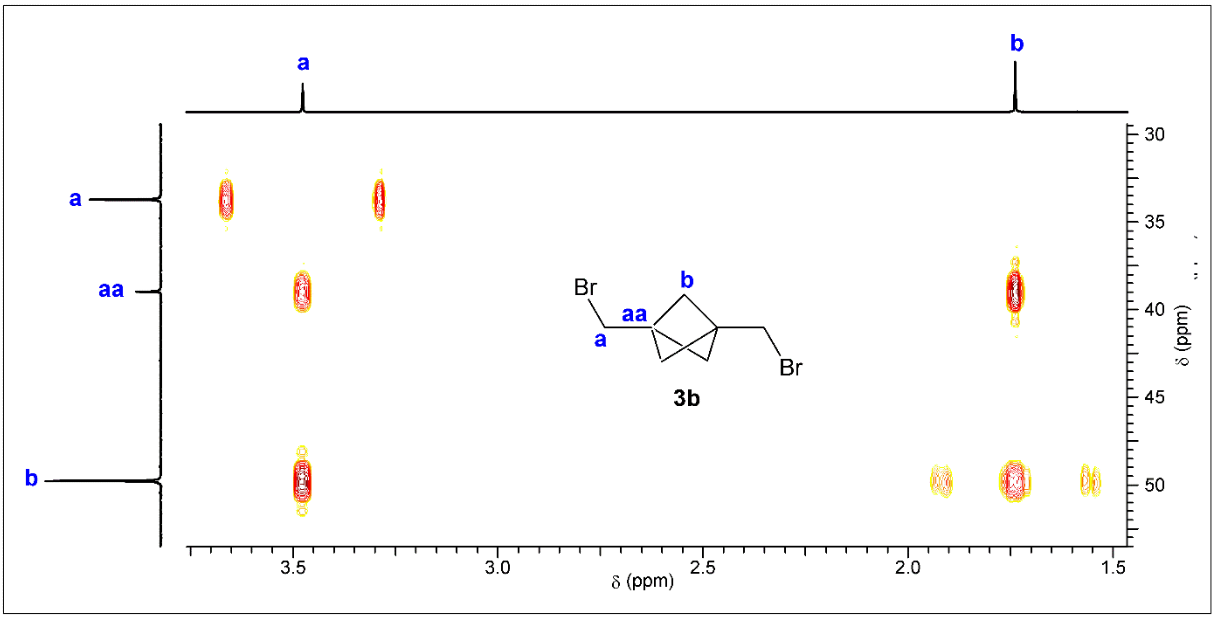

Figure S13 The ^{1}H-^{13}C HMBC NMR ($CDCl_3$) spectrum of compound **3b**.

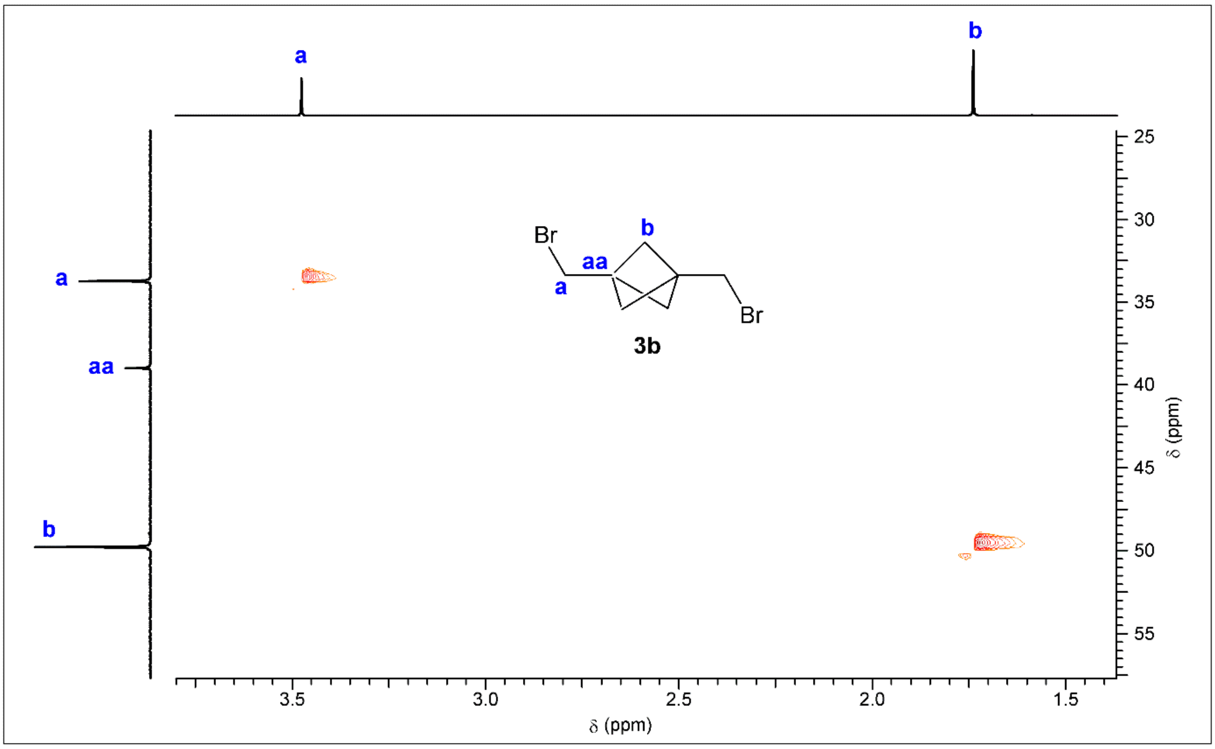

Figure S14 The ^{1}H–^{13}C HSQC NMR ($CDCl_3$) spectrum of compound **3b**.

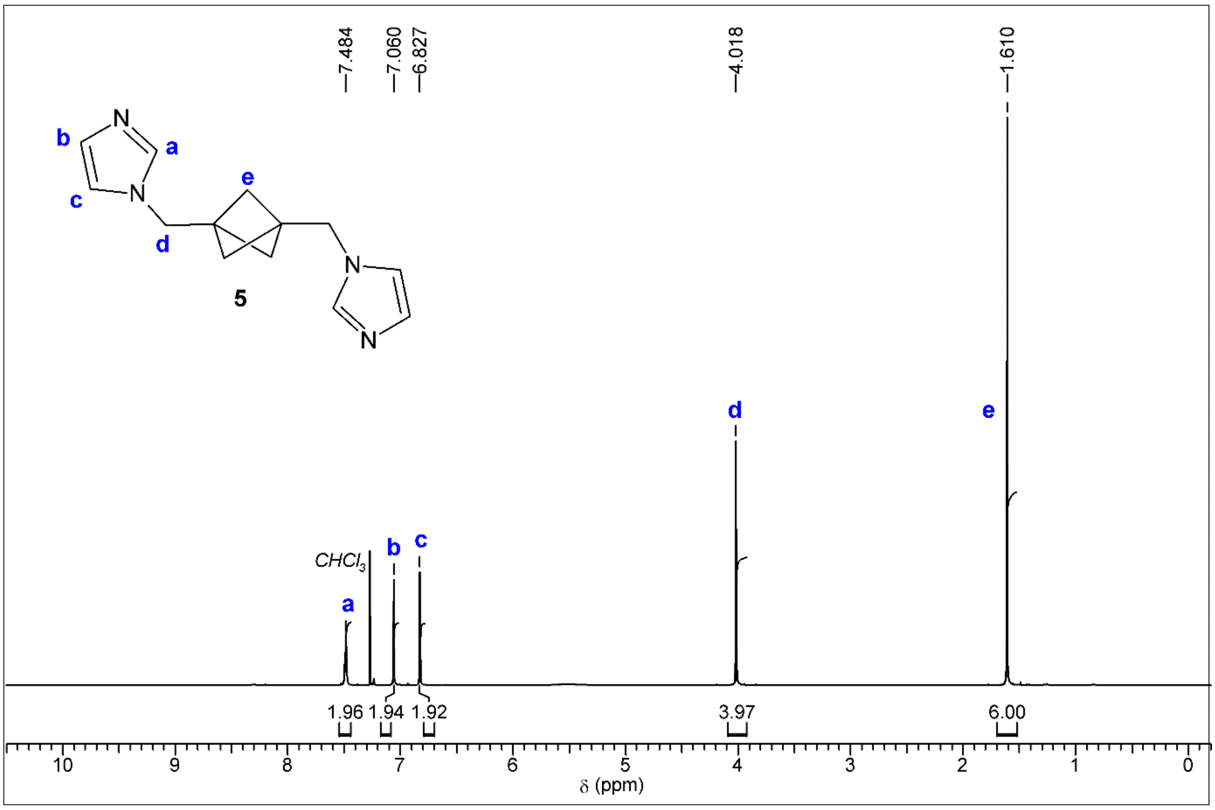

Figure S15 The ^{1}H NMR ($CDCl_3$, 401 MHz) spectrum of compound **5**.

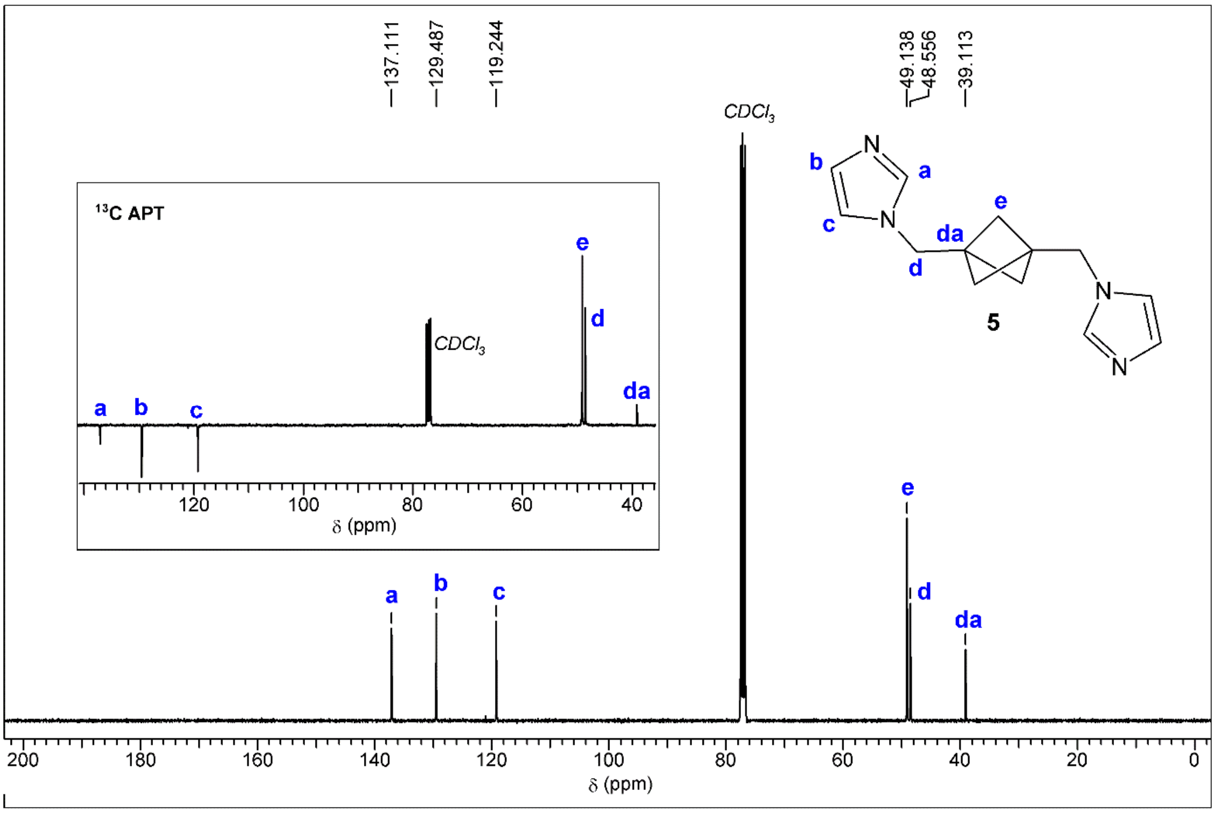

Figure S16 The $^{13}C\{^{1}H\}$ and ^{13}C APT NMR ($CDCl_3$, 101 MHz) spectrum of compound **5**.

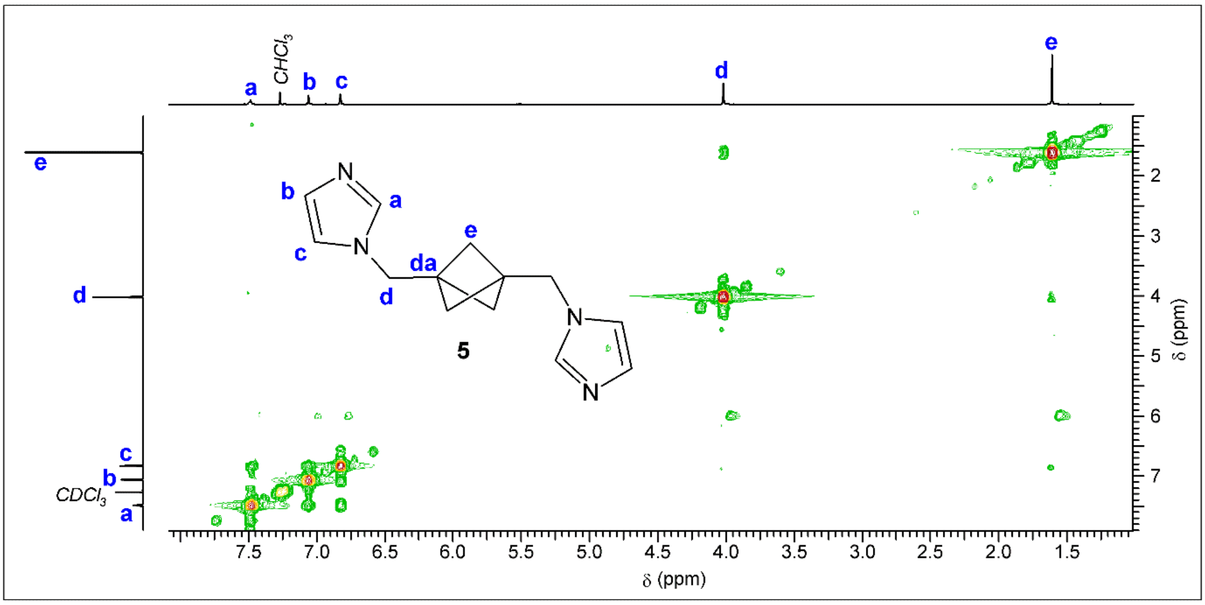

Figure S17 The ^{1}H–^{1}H COSY NMR ($CDCl_3$) spectrum of compound **5**.

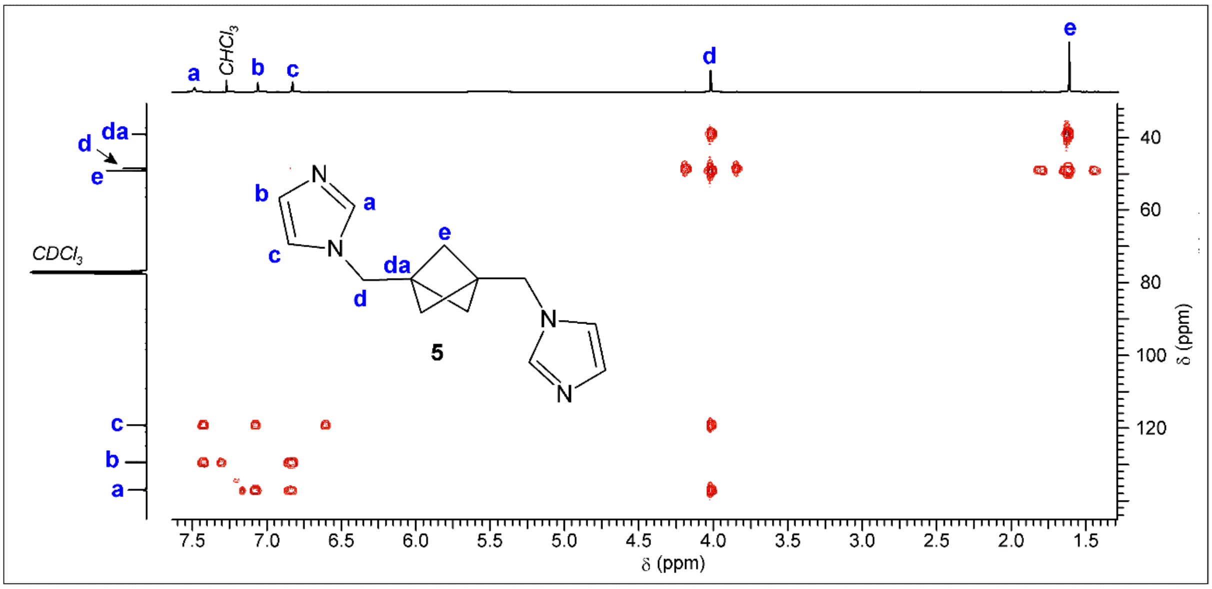

Figure S18 The ^{1}H–^{13}C HMBC NMR ($CDCl_3$) spectrum of compound **5**.

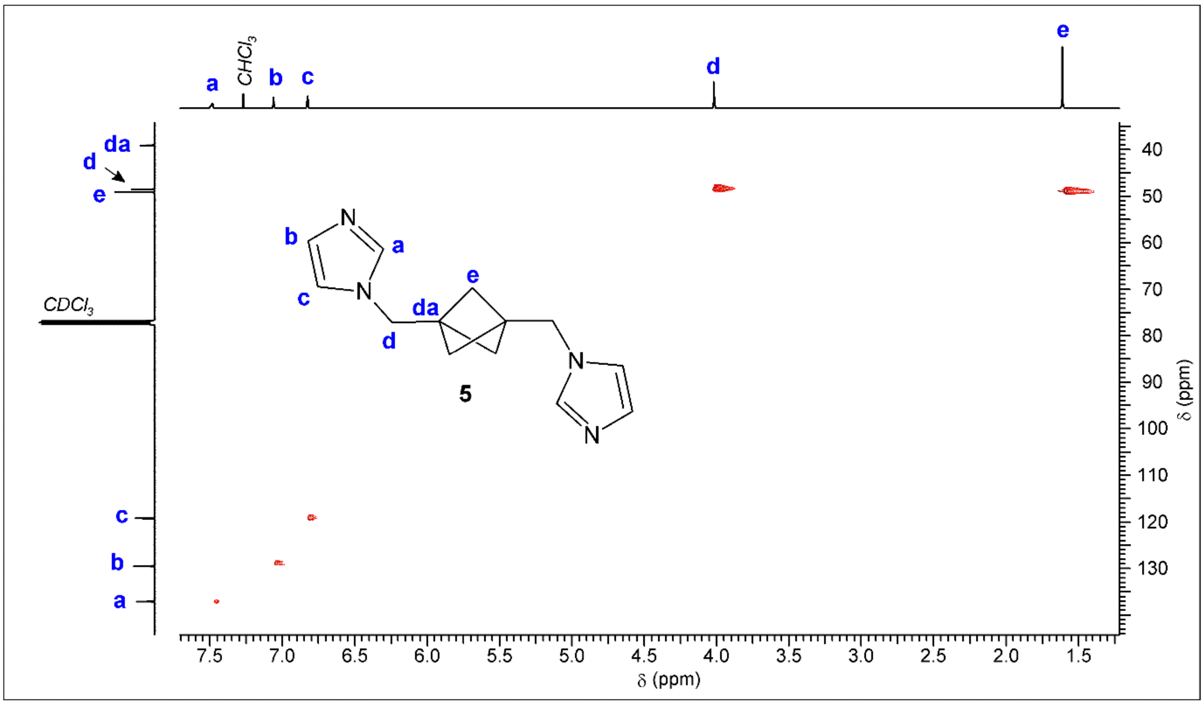

Figure S19 The ^{1}H–^{13}C HSQC NMR ($CDCl_3$) spectrum of compound **5**.

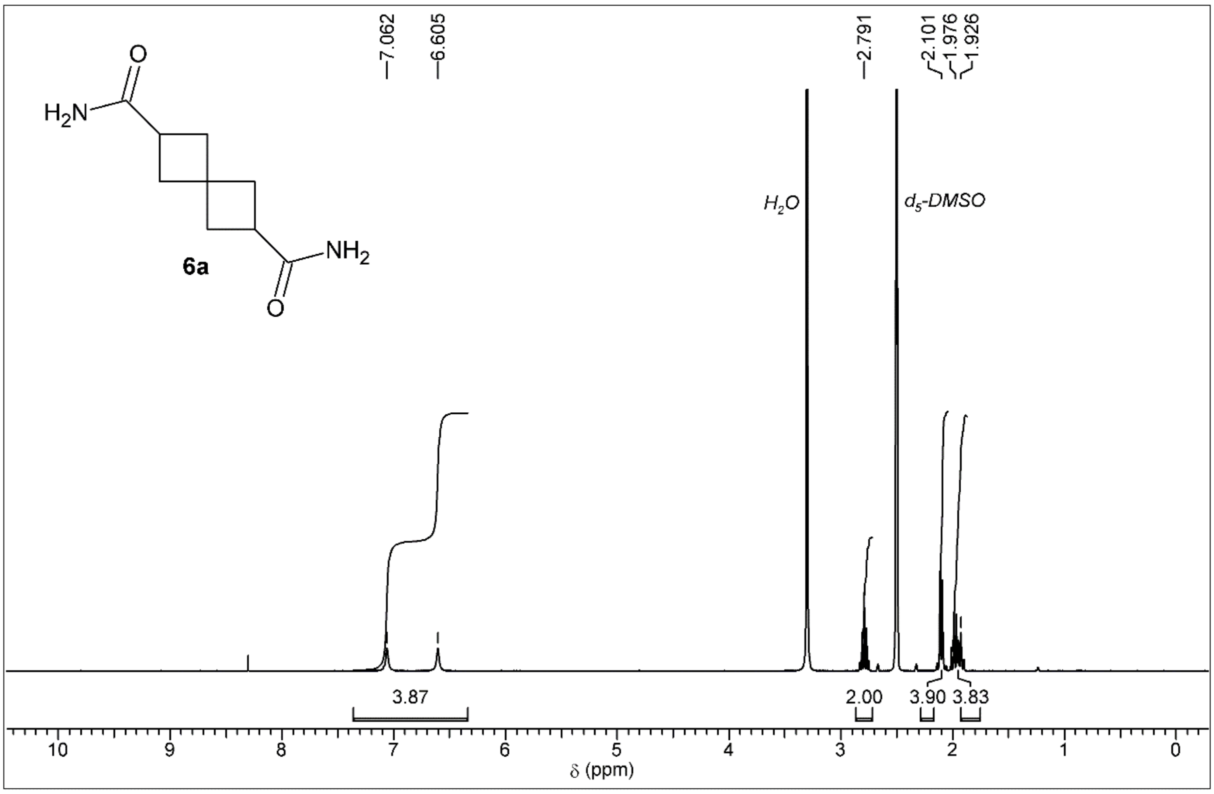

Figure S20 The ^{1}H NMR (d_6-DMSO, 400 MHz) spectrum of compound **6a**.

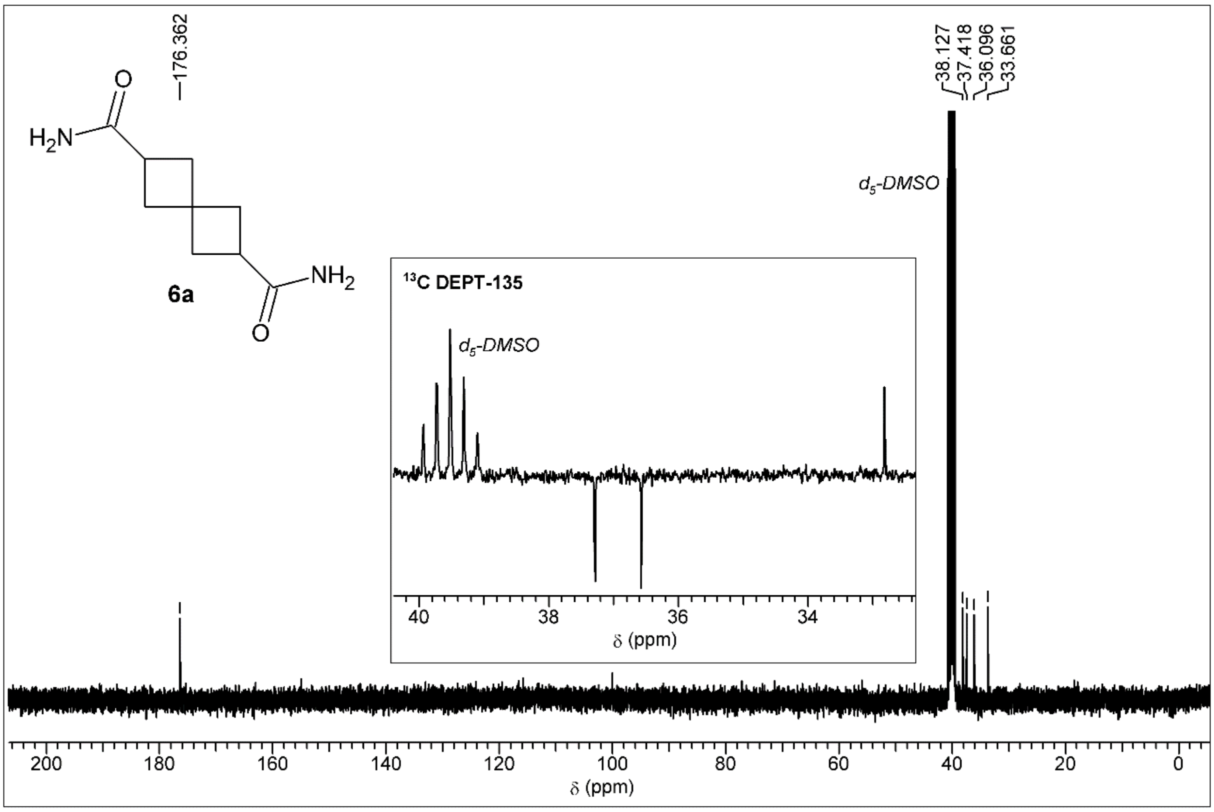

Figure S21 The $^{13}C\{^1H\}$ and ^{13}C DEPT-135 NMR (d_6-DMSO, 101 MHz) spectrum of compound **6a**.

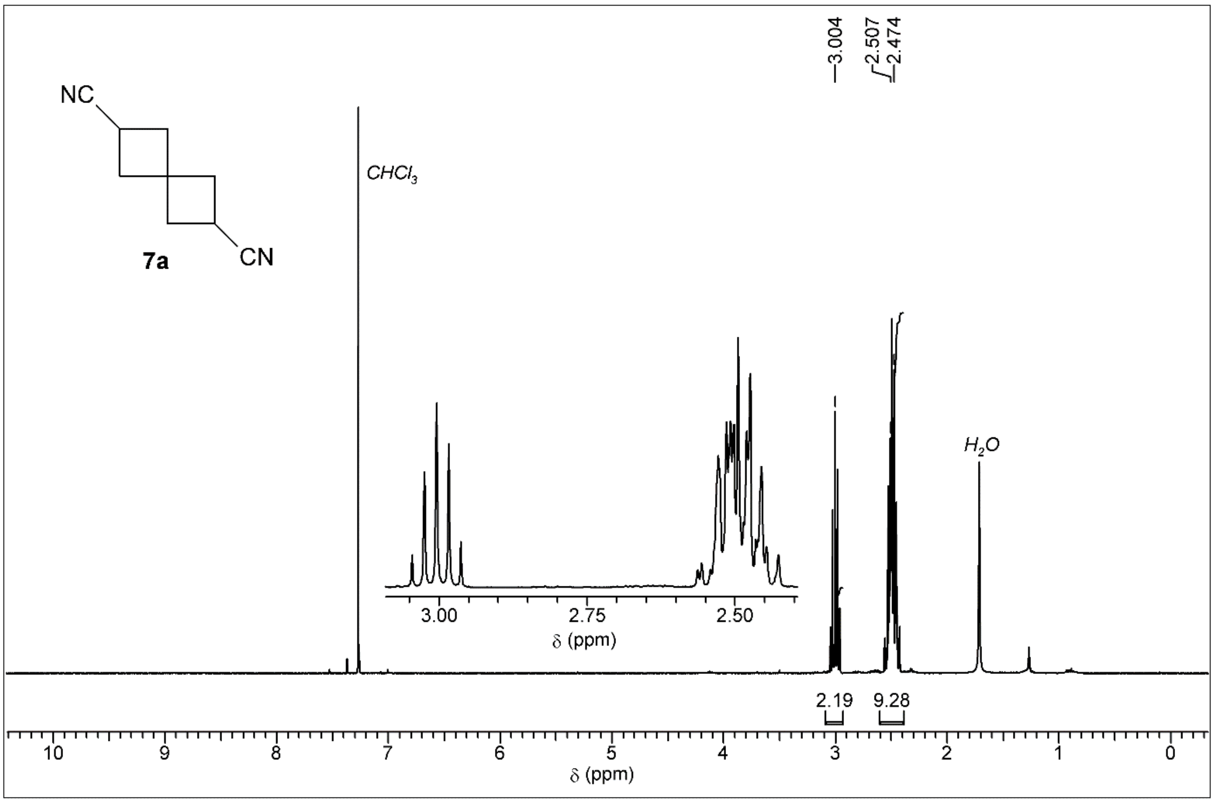

Figure S22 The ^1H NMR ($CDCl_3$, 400 MHz) spectrum of compound **7a**.

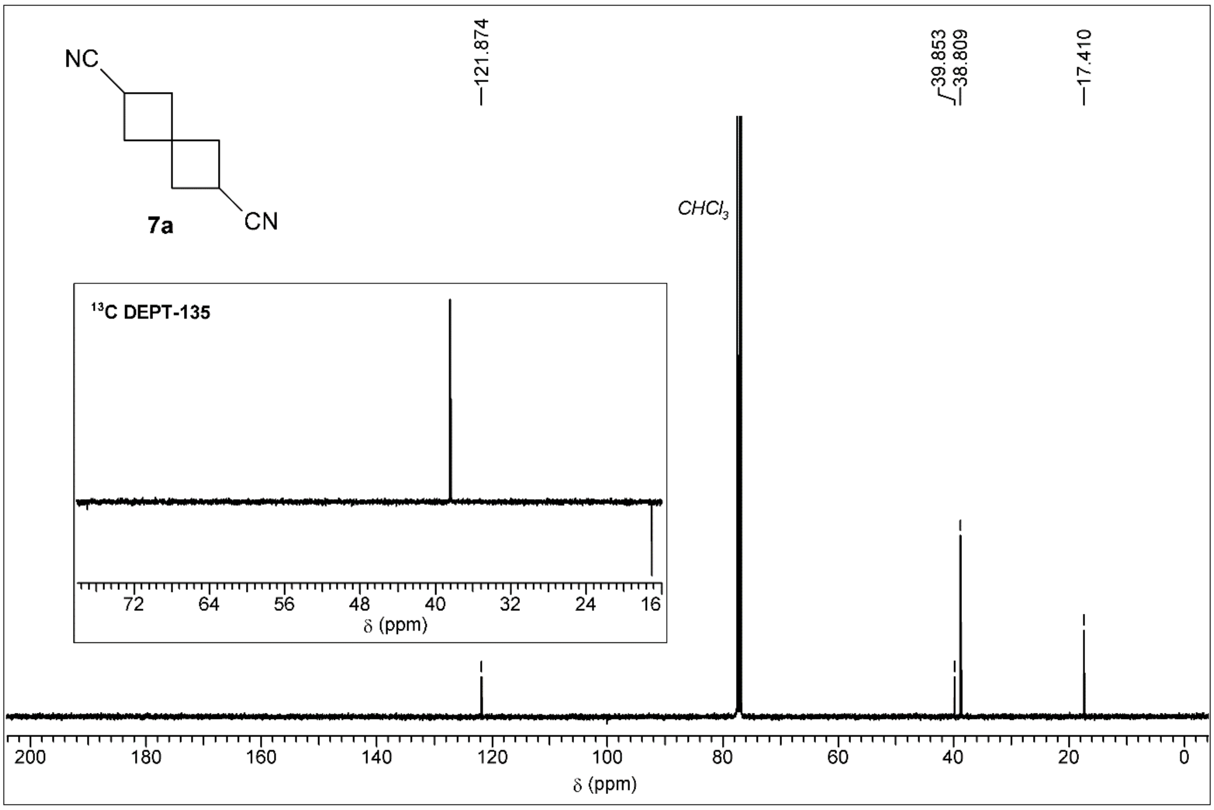

Figure S23 The $^{13}C\{^{1}H\}$ and ^{13}C DEPT-135 NMR ($CDCl_3$, 101 MHz) spectrum of compound **7a**.

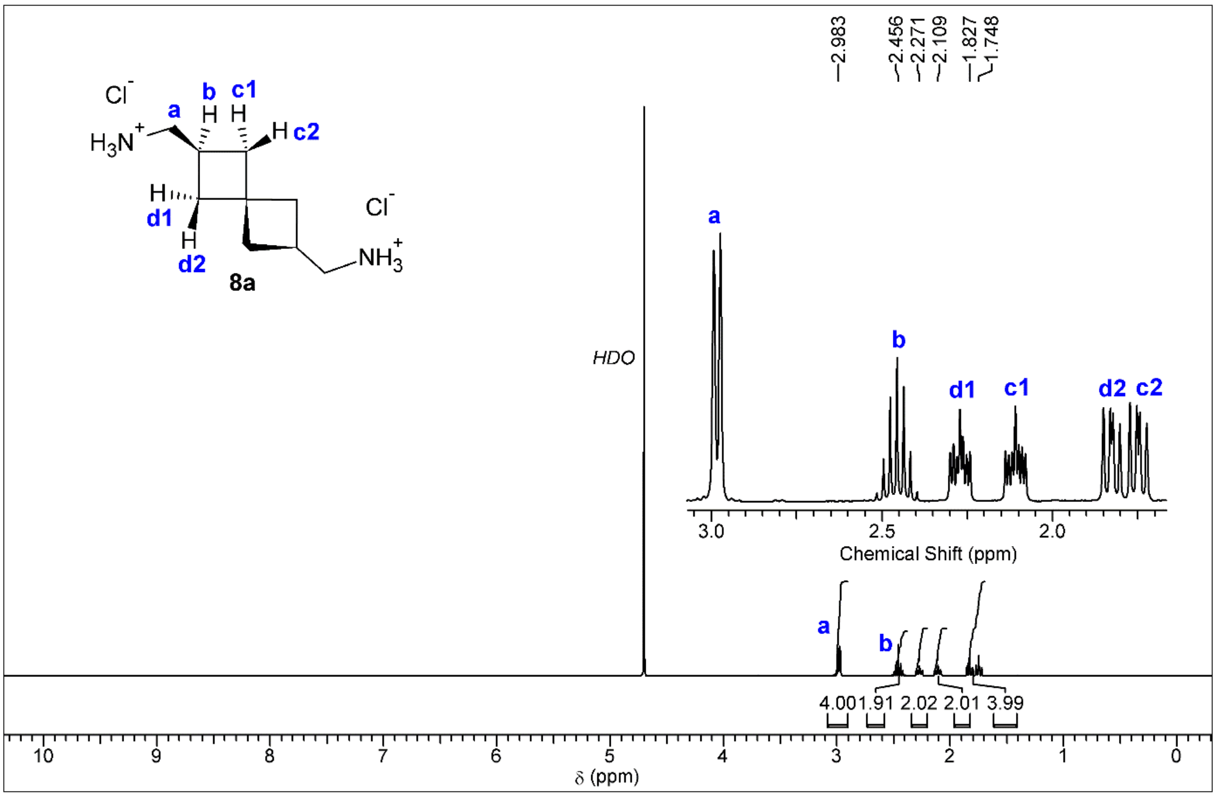

Figure S24 The ^{1}H NMR (D_2O, 400 MHz) spectrum of compound **8a**.

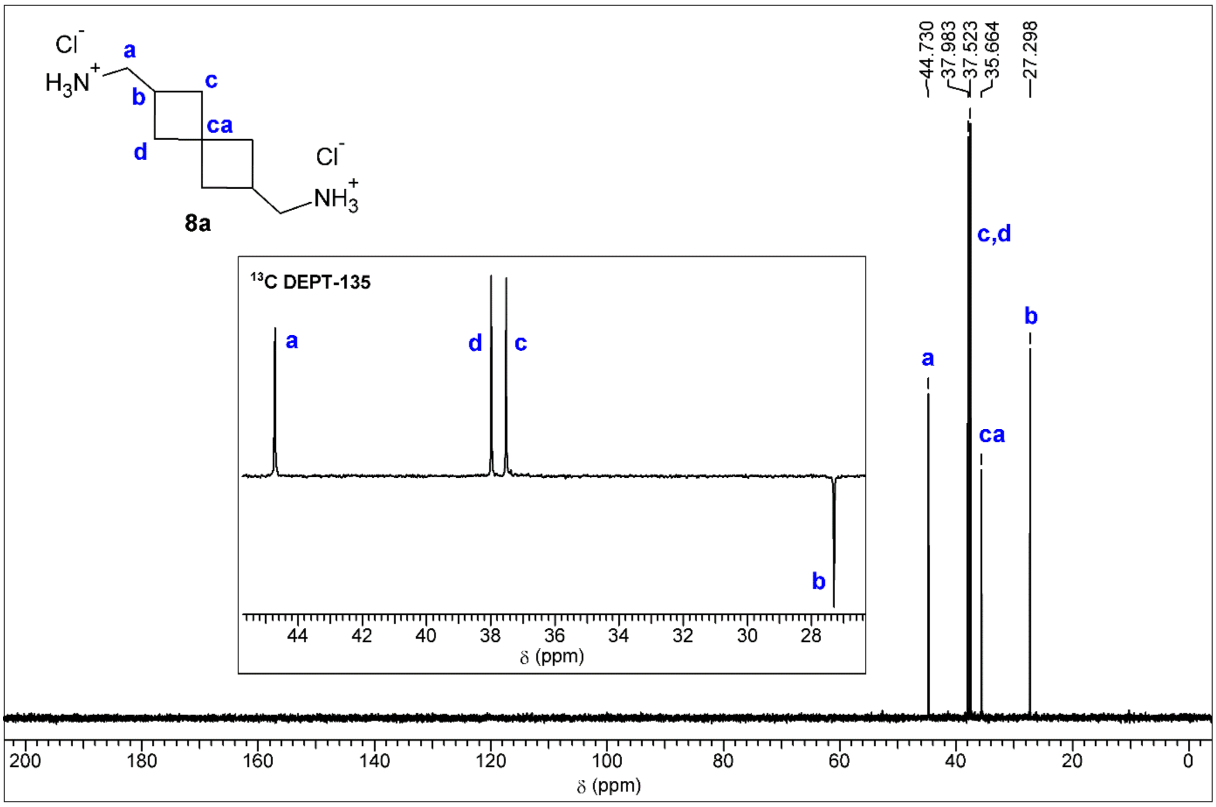

Figure S25 The $^{13}C\{^{1}H\}$ and ^{13}C DEPT-135 NMR (D_2O, 101 MHz) spectrum of compound **8a**.

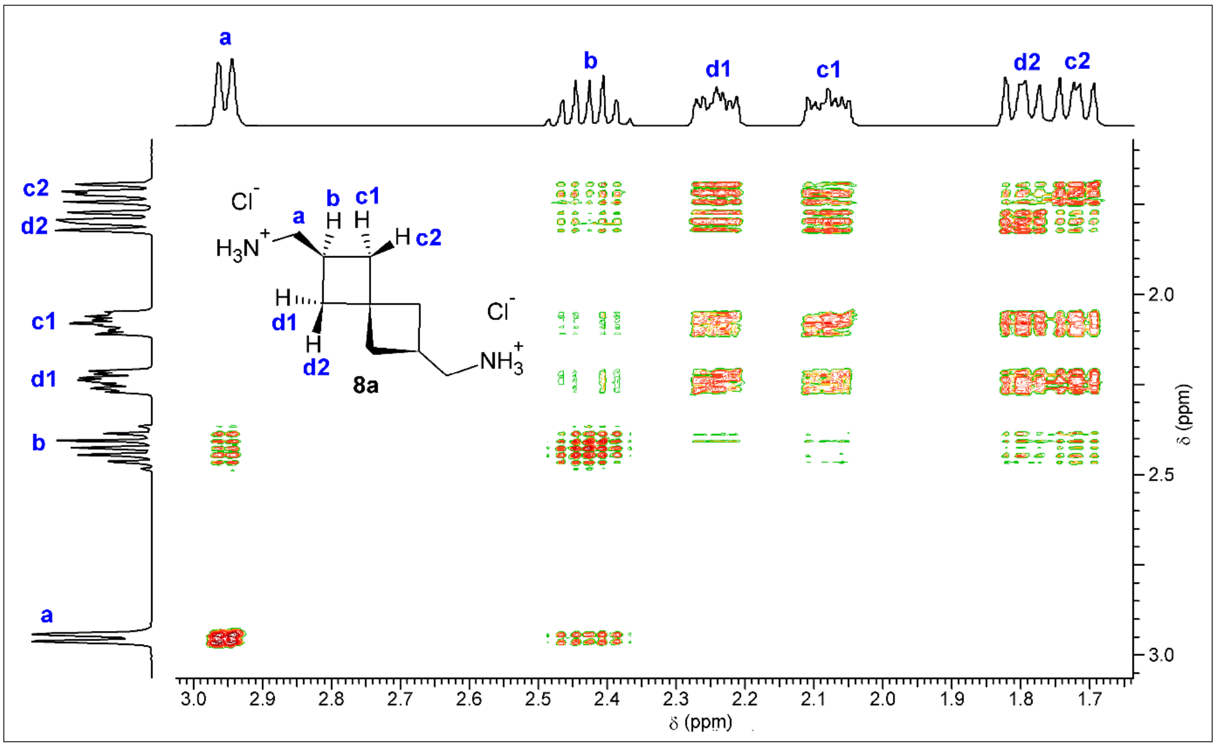

Figure S26 The ^{1}H–^{1}H COSY NMR (D_2O) spectrum of compound **8a**.

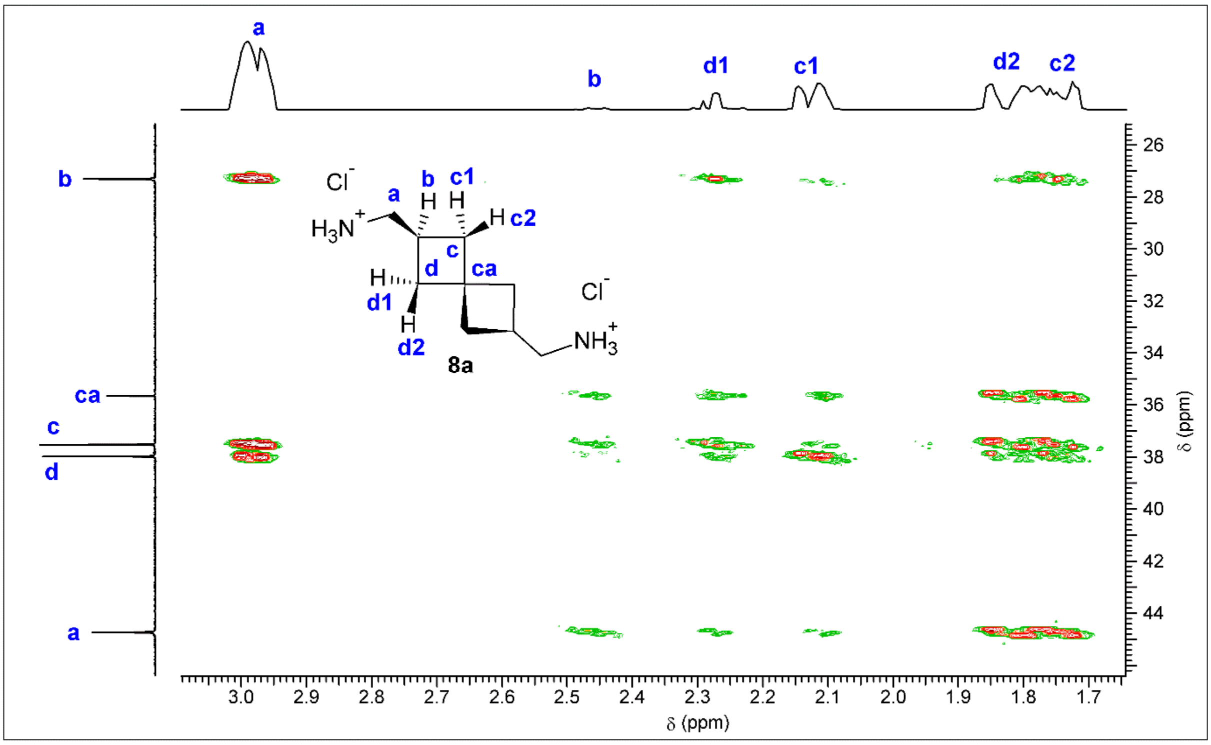

Figure S27 The ^{1}H–^{13}C HMBC NMR (D_2O) spectrum of compound **8a**.

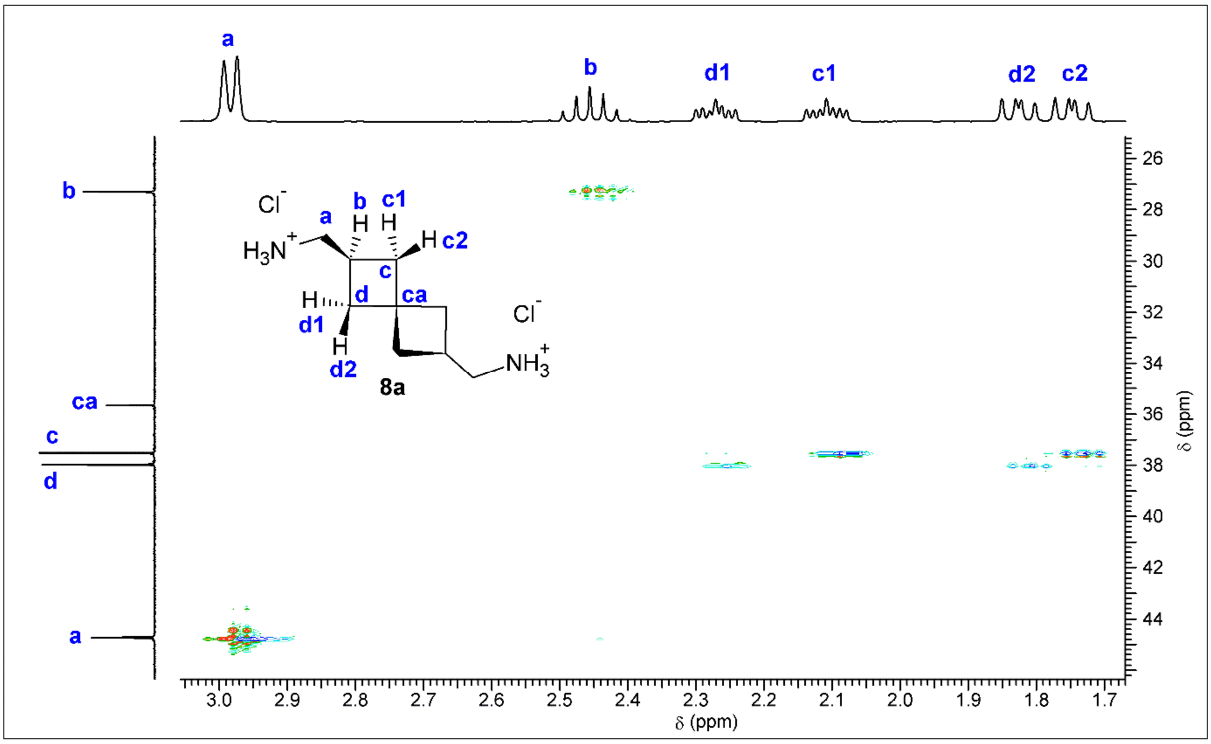

Figure S28 The edited ^{1}H–^{13}C HSQC NMR (D_2O) spectrum of compound **8a**.

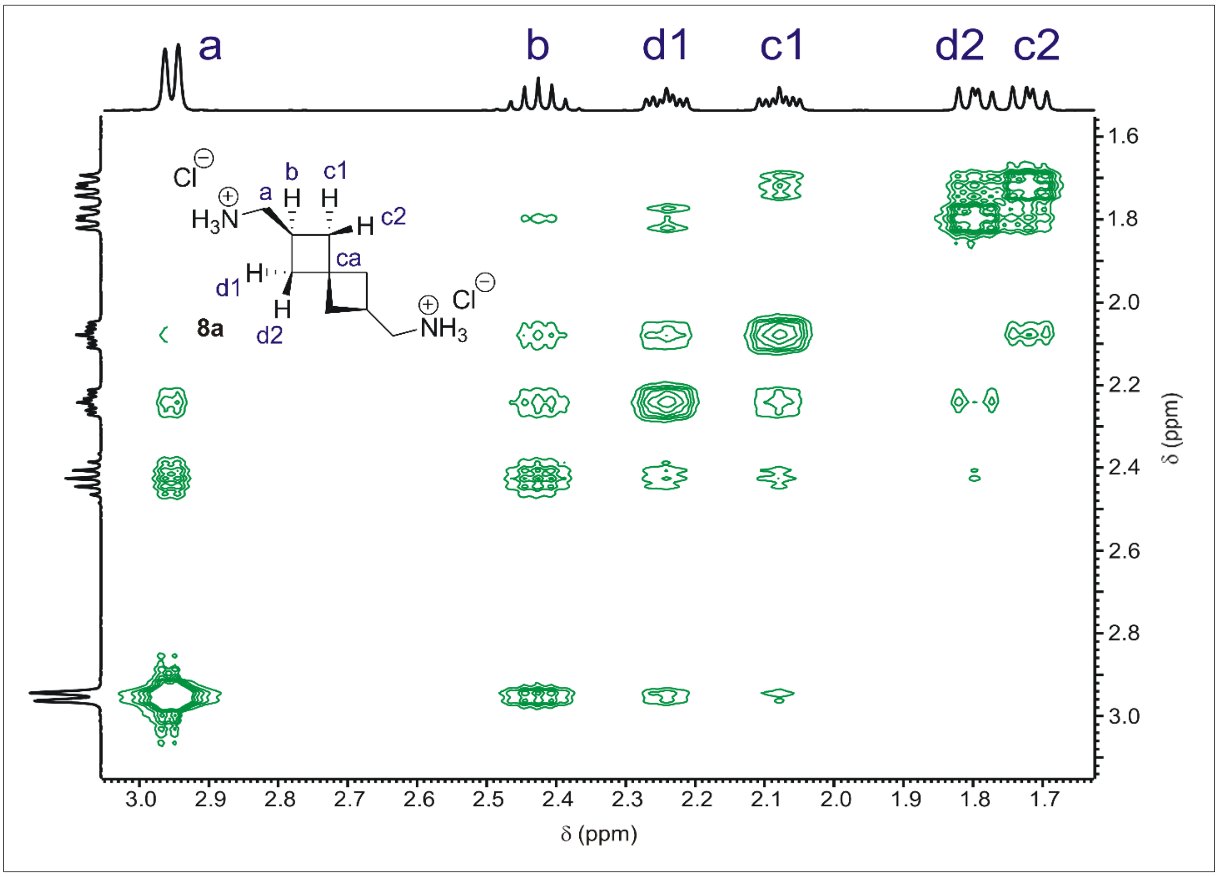

Figure S29 The ^1H–^1H ROESY NMR (D_2O) spectrum of compound **8a**.

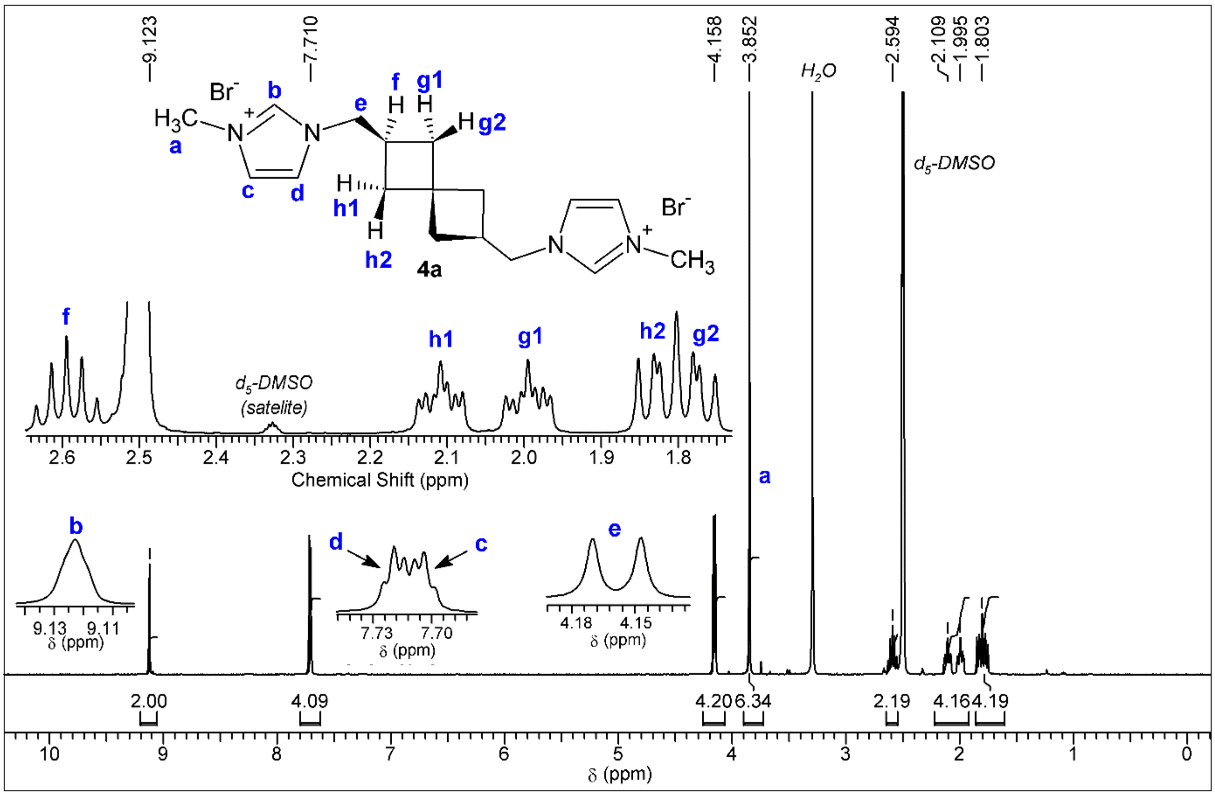

Figure S30 The ^1H NMR (d_6-DMSO, 400 MHz) spectrum of compound **4a**.

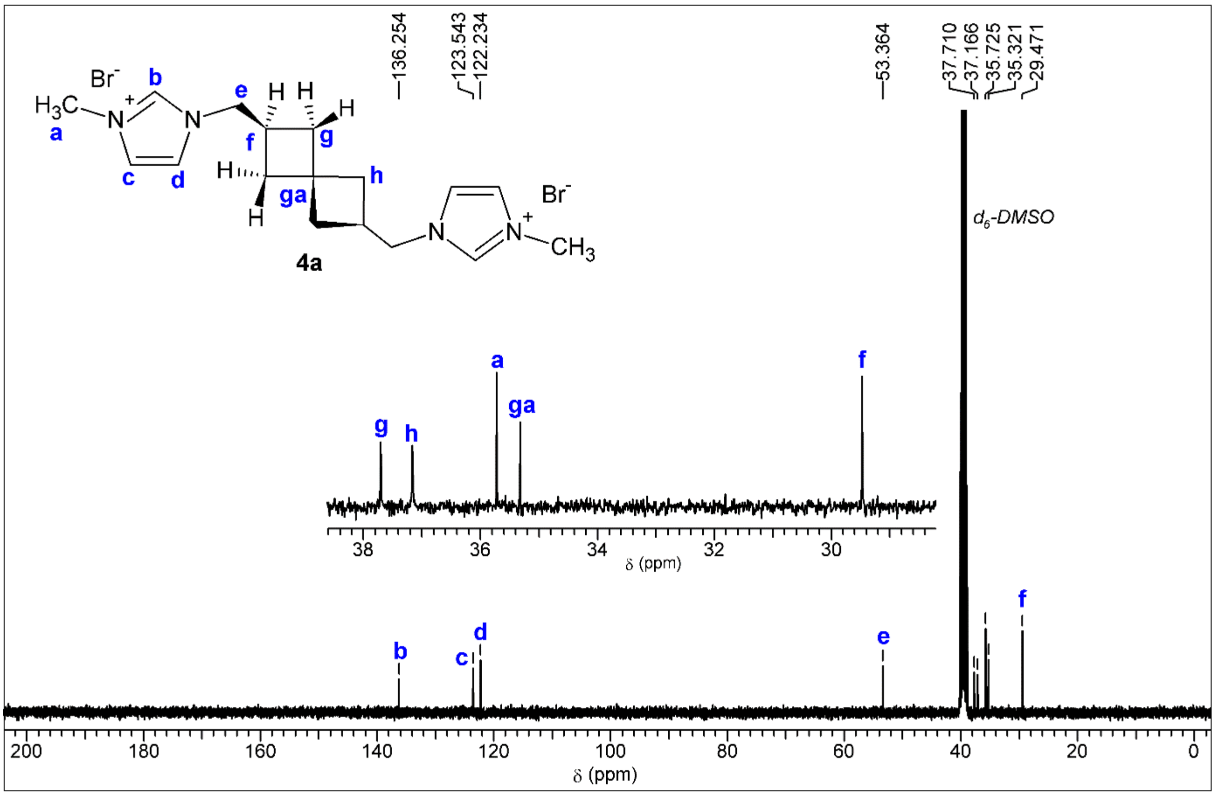

Figure S31 The $^{13}C\{^1H\}$ NMR (d_6-DMSO, 101 MHz) spectrum of compound **4a**.

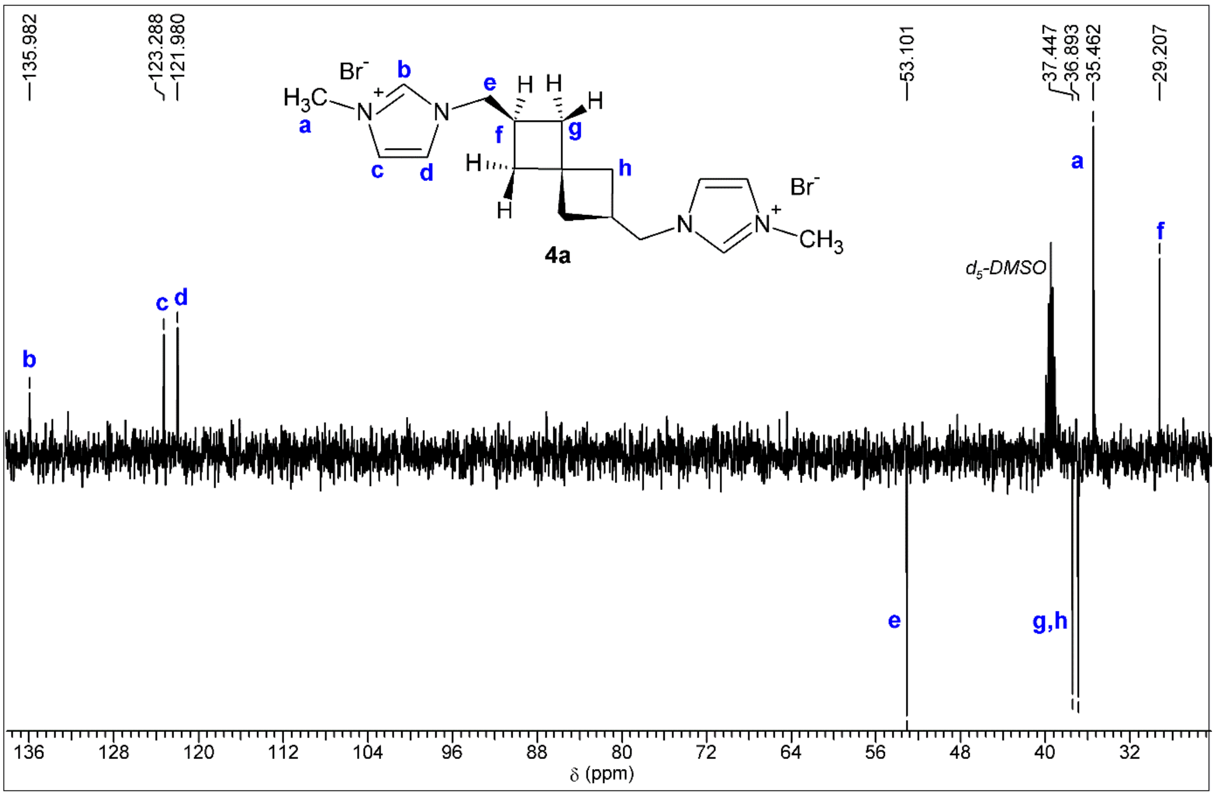

Figure S32 The ^{13}C DEPT-135 NMR (d_6-DMSO, 101 MHz) spectrum of compound **4a**.

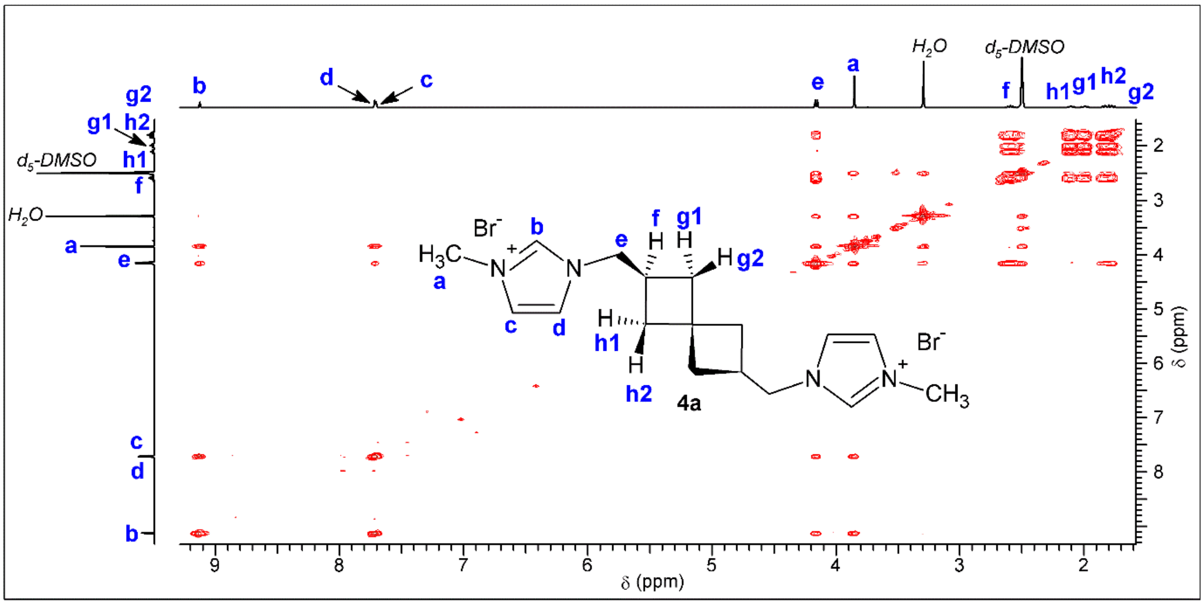

Figure S33 The ^{1}H–^{1}H COSY NMR (d_6-DMSO) spectrum of compound **4a**.

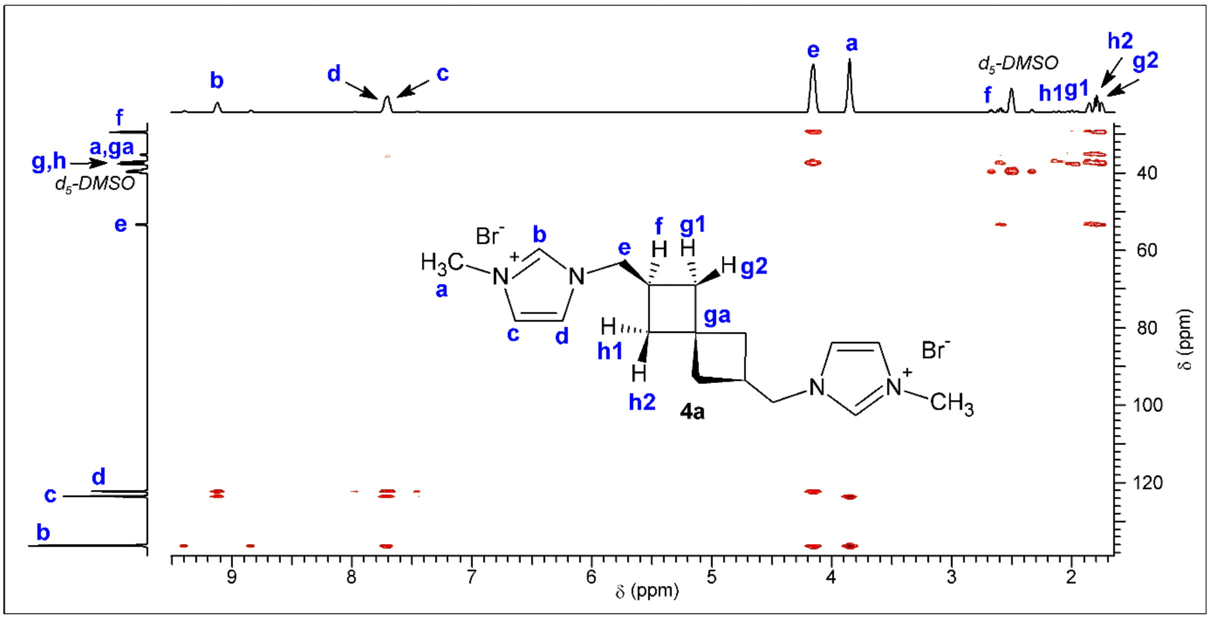

Figure S34 The ^{1}H–^{13}C HMBC NMR (d_6-DMSO) spectrum of compound **4a**.

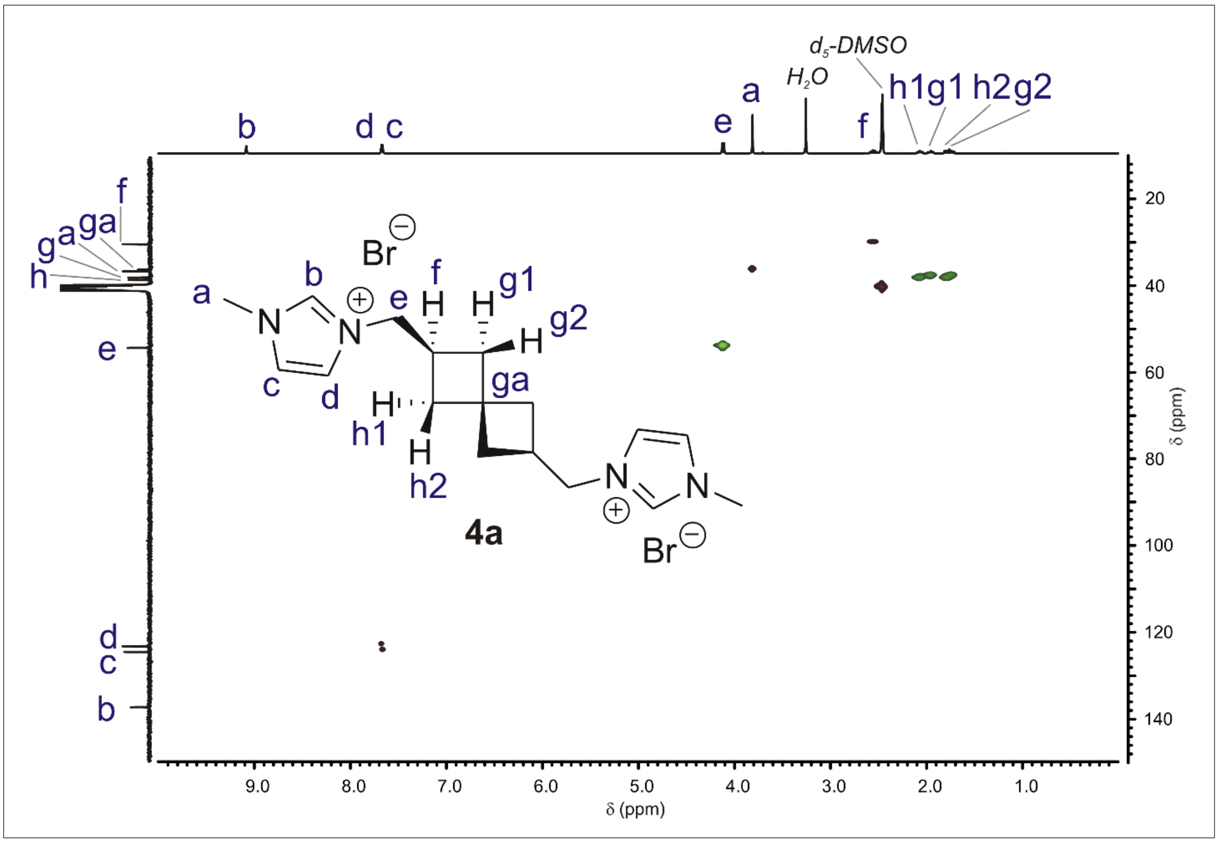

Figure S35 The edited ^{1}H–^{13}C HSQC NMR (d_6-DMSO) spectrum of compound **4a**.

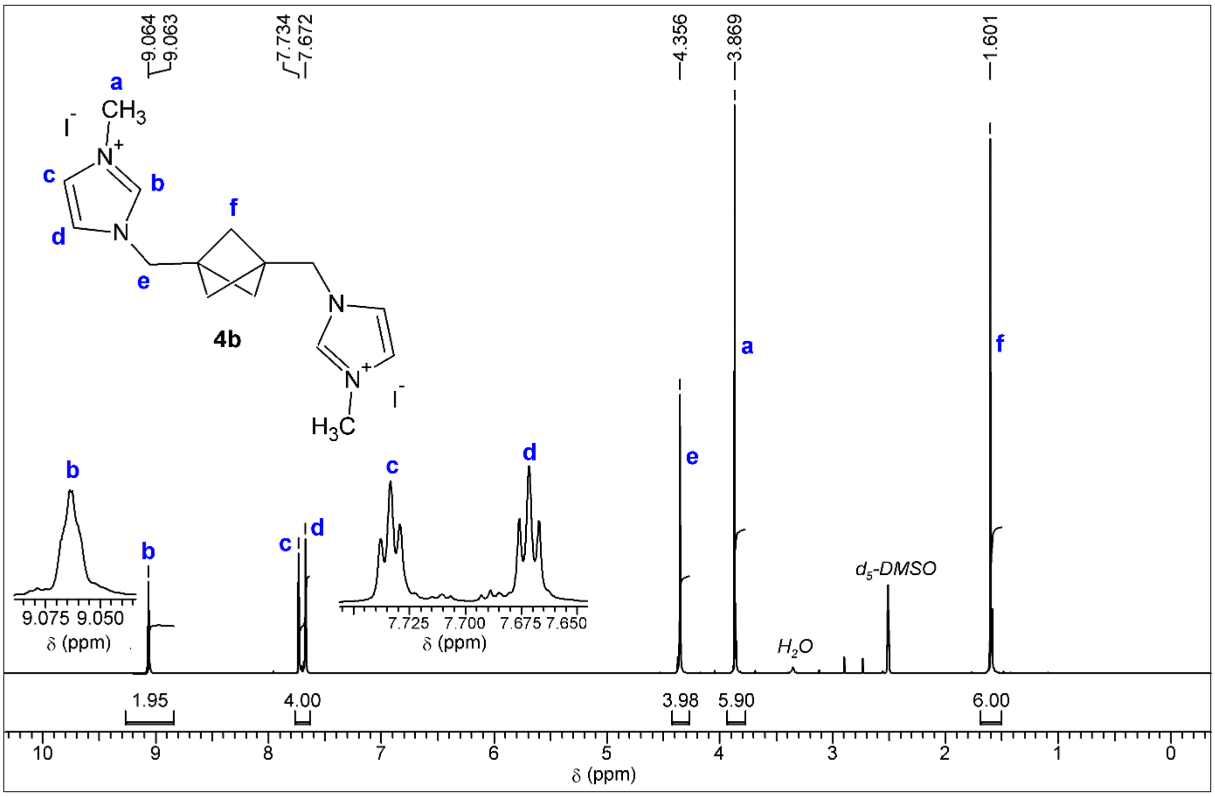

Figure S36 The ^{1}H NMR (d_6-DMSO, 401 MHz) spectrum of compound **4b**.

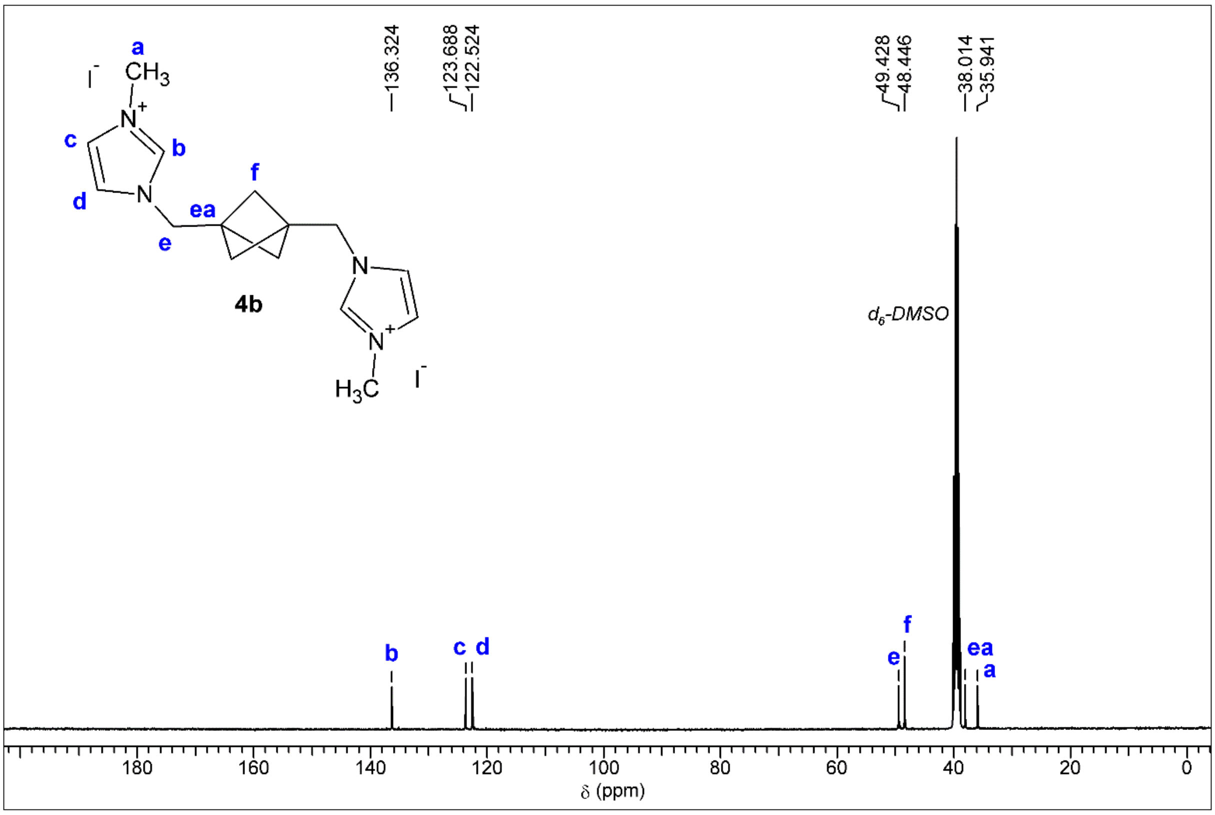

Figure S37 The $^{13}C\{^{1}H\}$ NMR (d_6-DMSO, 101 MHz) spectrum of compound **4b**.

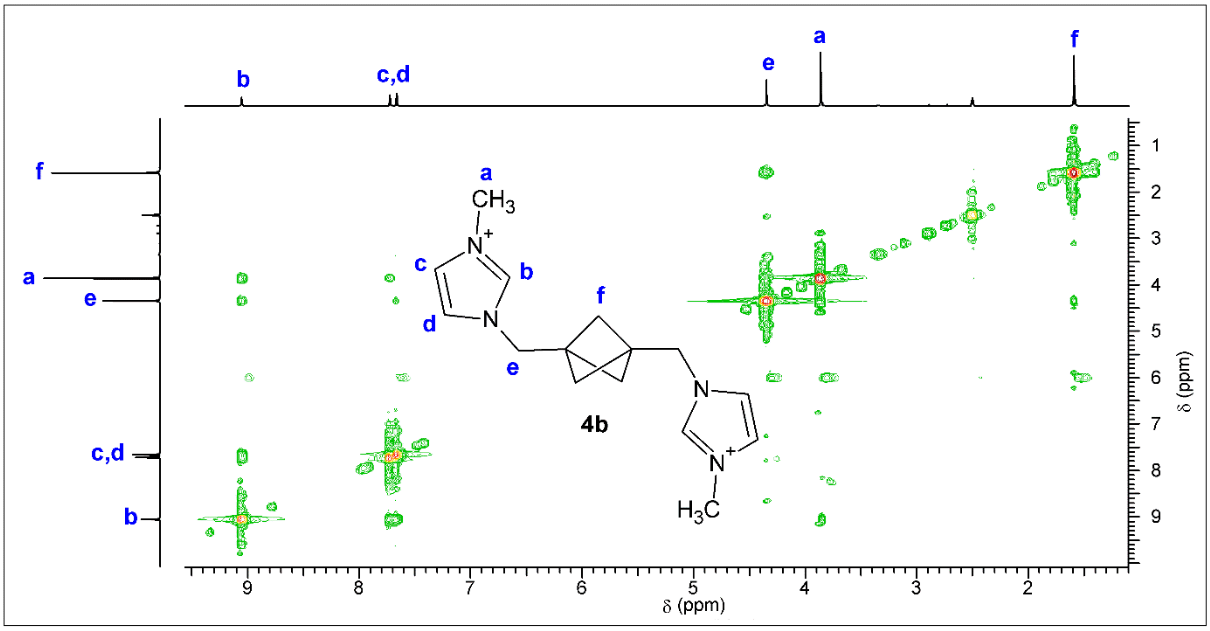

Figure S38 The ^{1}H–^{1}H COSY NMR (d_6-DMSO) spectrum of compound **4b**.

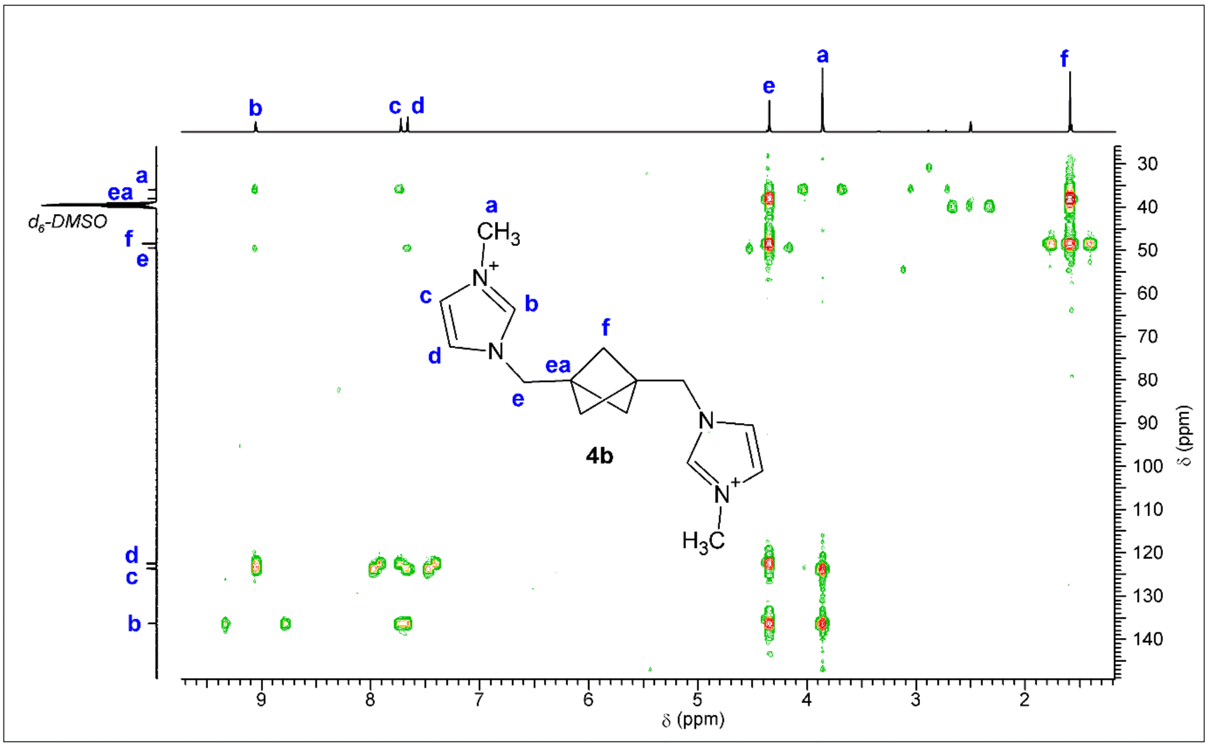

Figure S39 The ^{1}H–^{13}C HMBC NMR (d_6-DMSO) spectrum of compound **4b**.

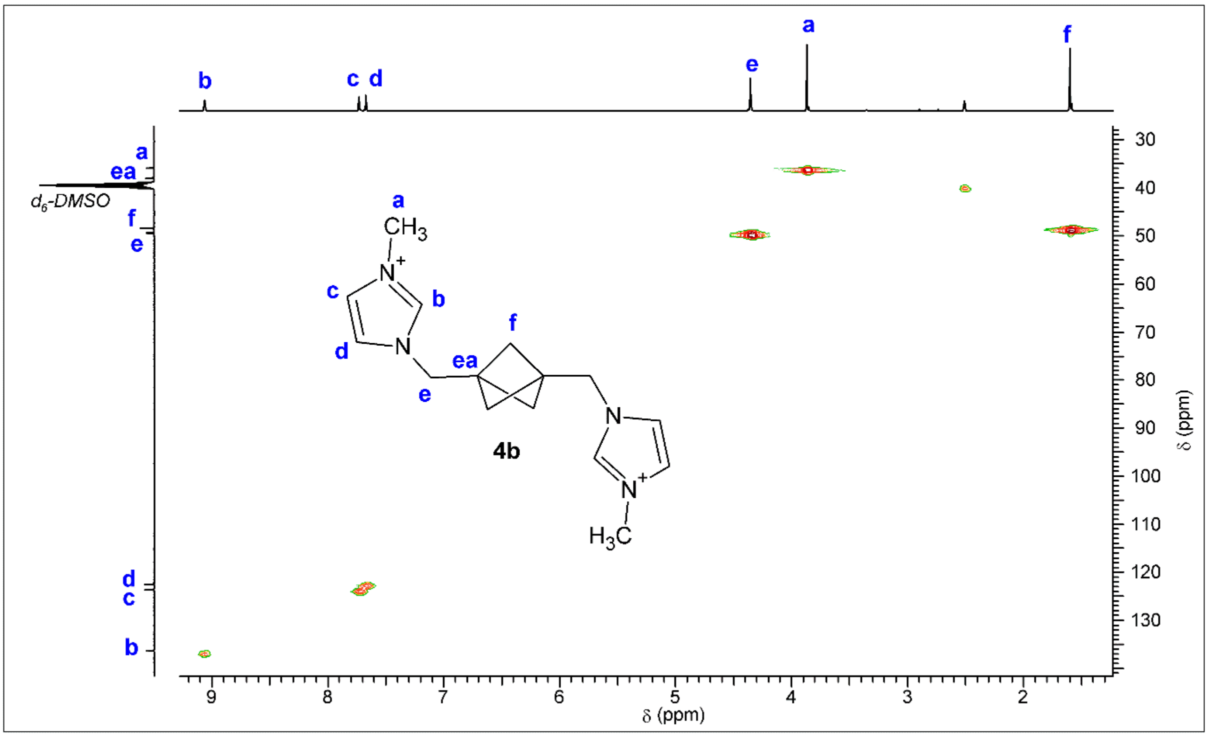

Figure S40 The ^{1}H–^{13}C HSQC NMR (d_6-DMSO) spectrum of compound **4b**.

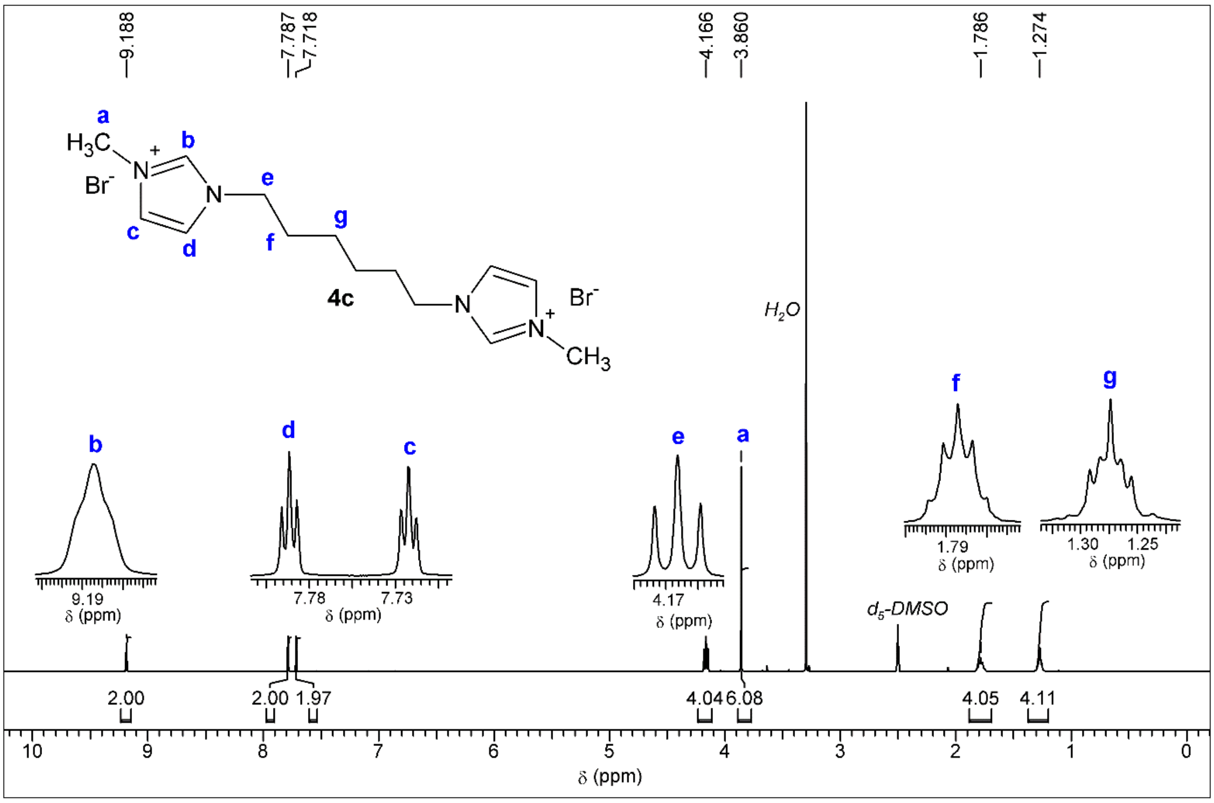

Figure S41 The ^{1}H NMR (d_6-DMSO, 400 MHz) spectrum of compound **4c**.

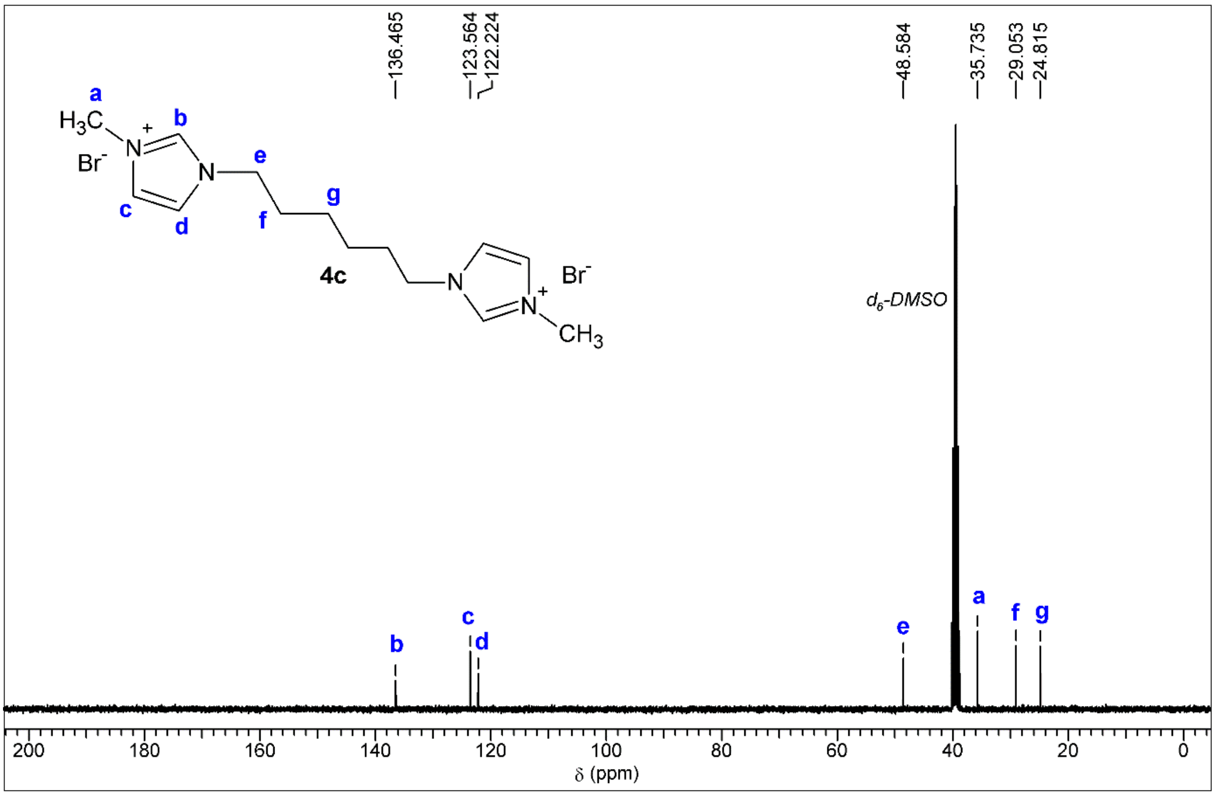

Figure S42 The ^{13}C{^{1}H} NMR (d_6-DMSO, 101 MHz) spectrum of compound **4c**.

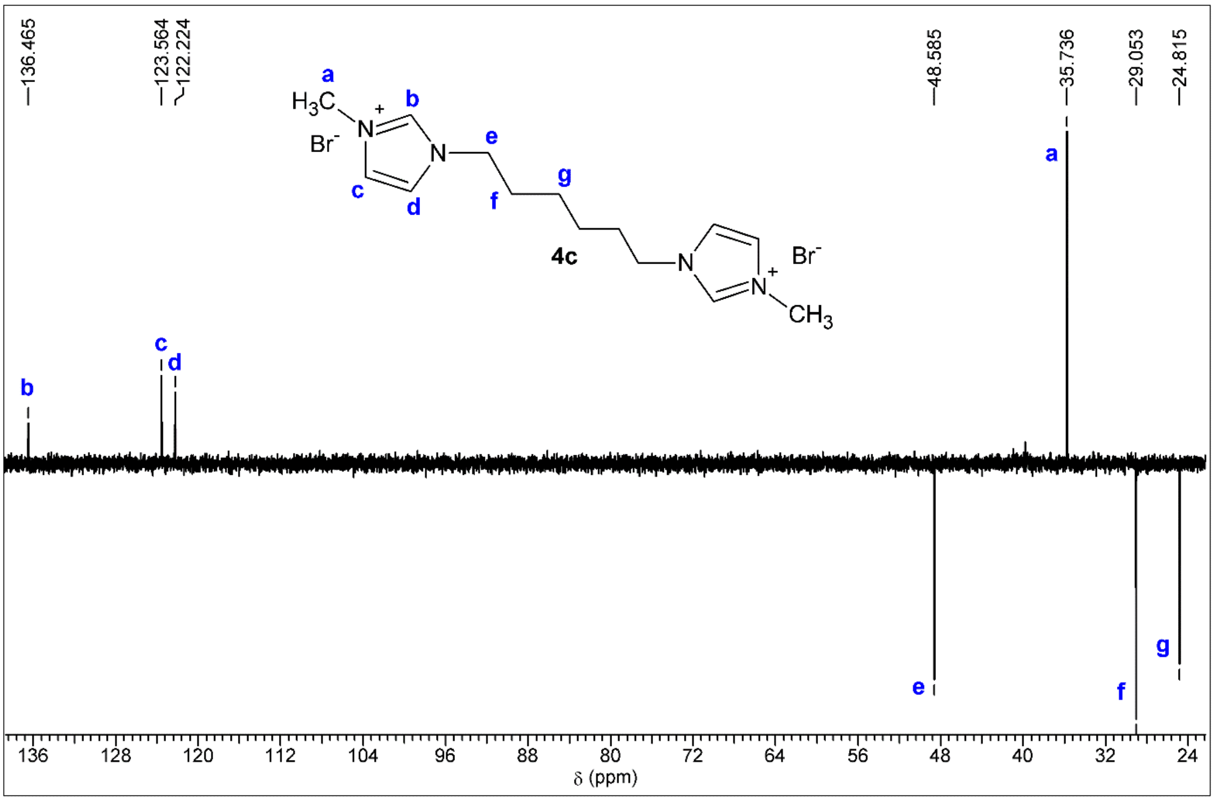

Figure S43 The ^{13}C DEPT-135 NMR (d_6-DMSO, 101 MHz) spectrum of compound **4c**.

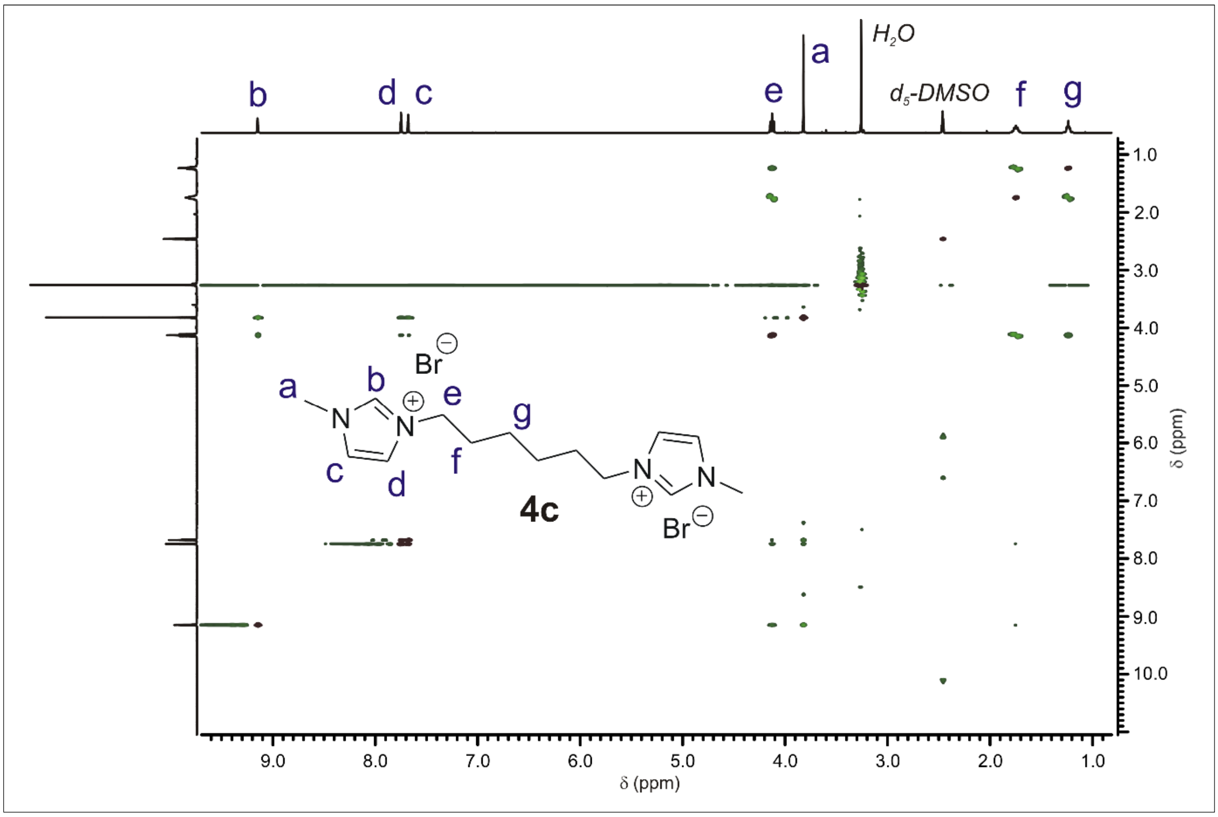

Figure S44 The ^{1}H–^{1}H ROESY NMR (d_6-DMSO) spectrum of compound **4c**.

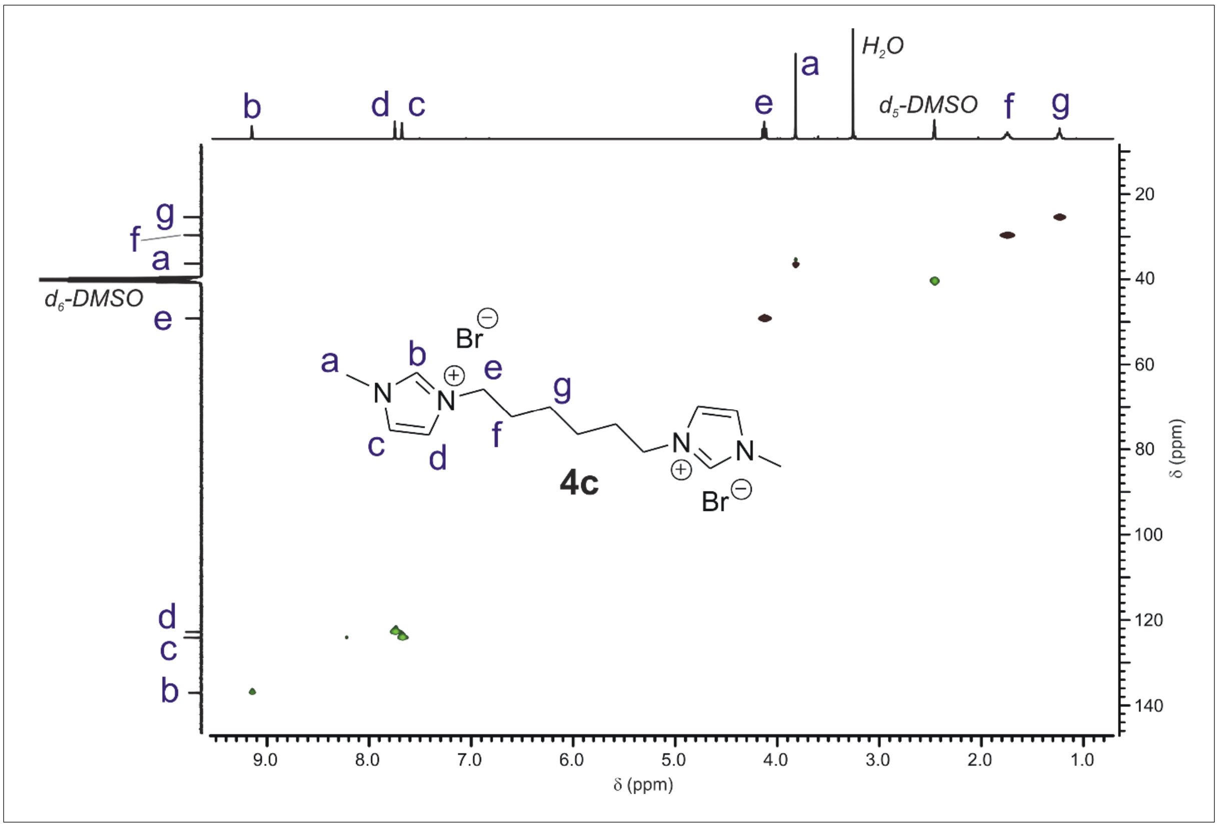

Figure S45 The edited ^{1}H–^{13}C HSQC NMR (d_6-DMSO) spectrum of compound **4c**.

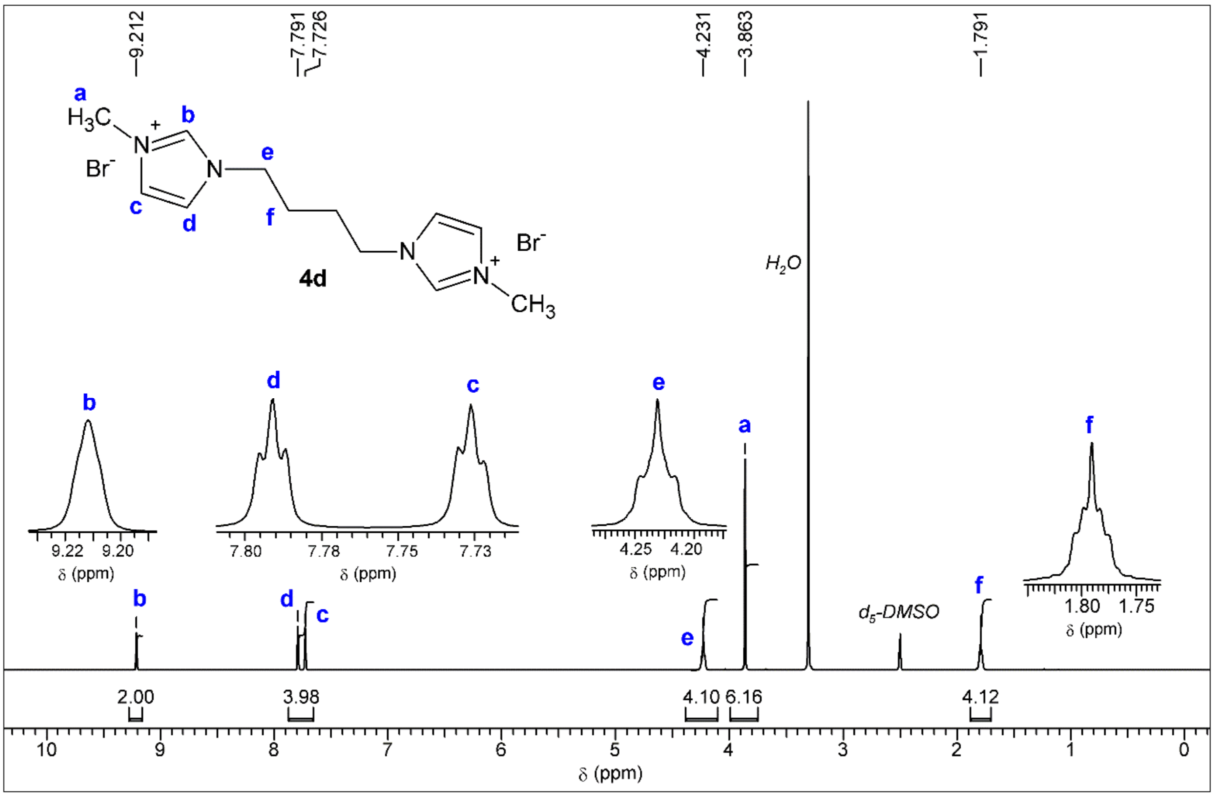

Figure S46 The ^{1}H NMR (d_6-DMSO, 400 MHz) spectrum of compound **4d**.

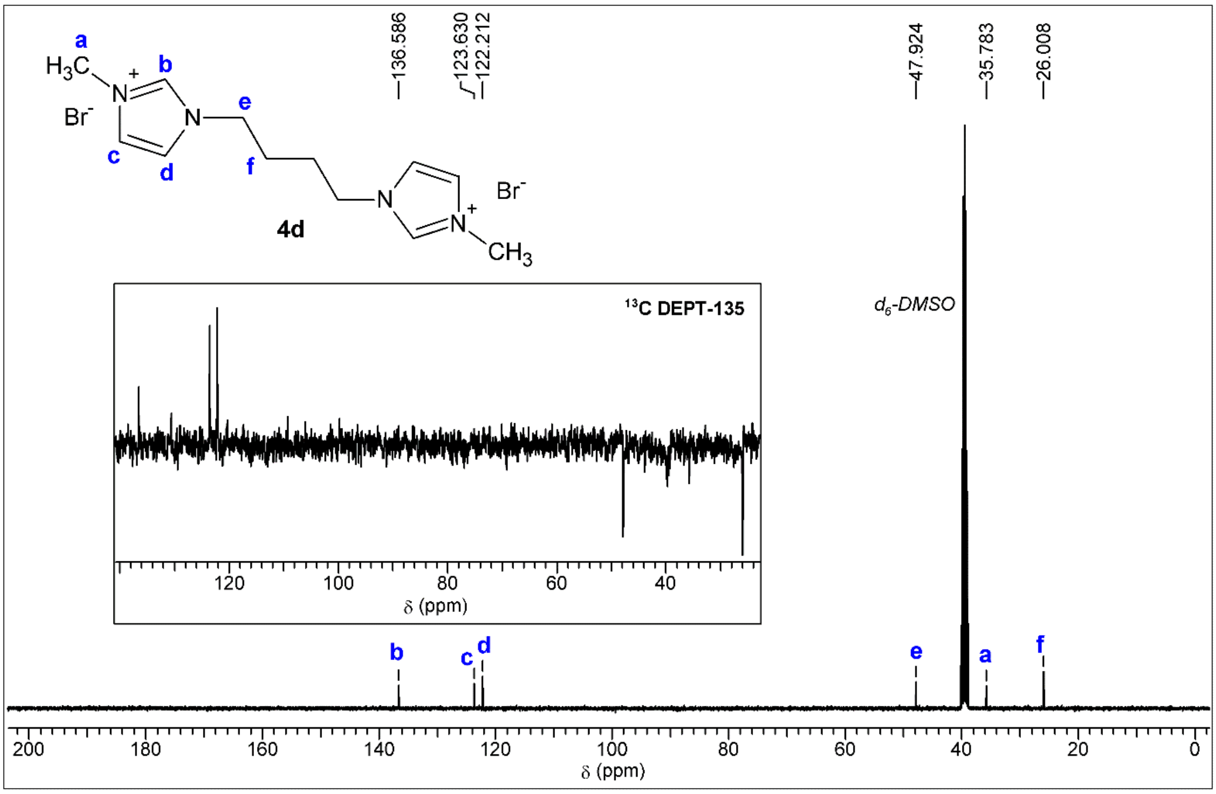

Figure S47 The $^{13}C\{^{1}H\}$ and ^{13}C DEPT-135 NMR (d_6-DMSO, 101 MHz) spectrum of compound **4d**.

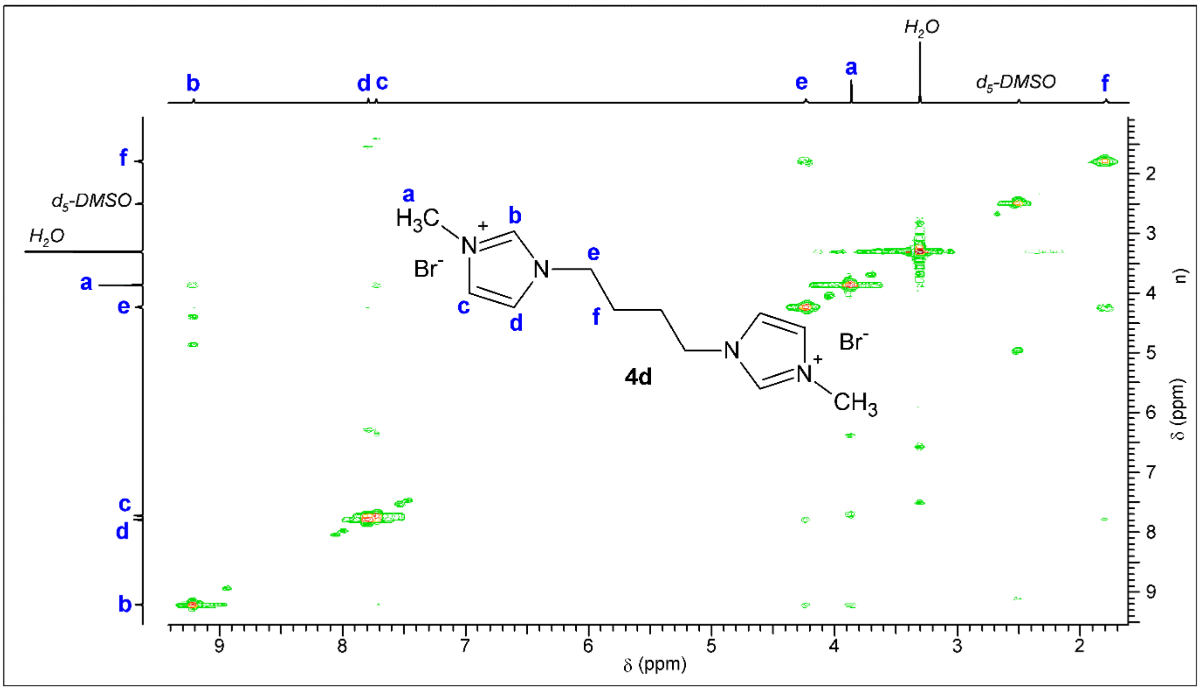

Figure S48 The ^{1}H–^{1}H ROESY NMR (d_6-DMSO) spectrum of compound **4d**.

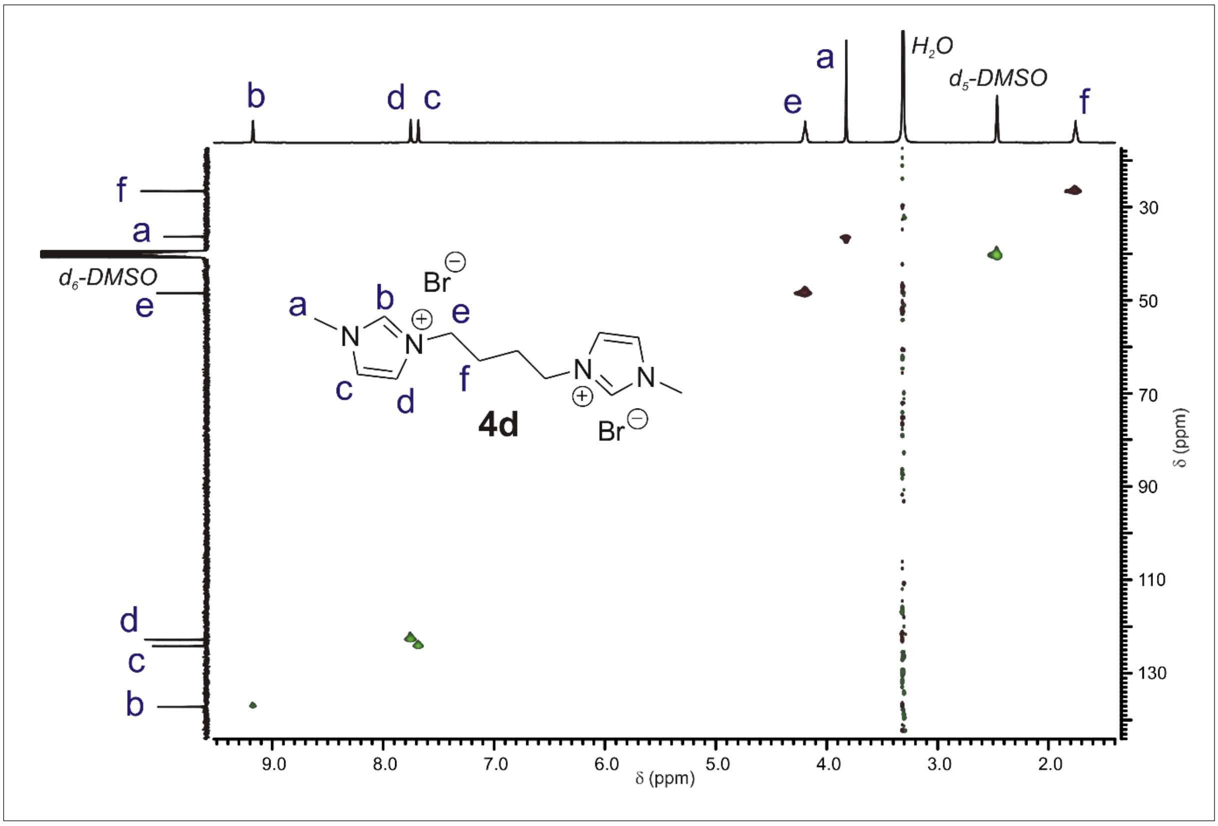

Figure S49 The edited ^{1}H–^{13}C HSQC NMR (d_6-DMSO) spectrum of compound **4d**.

NMR titrations data

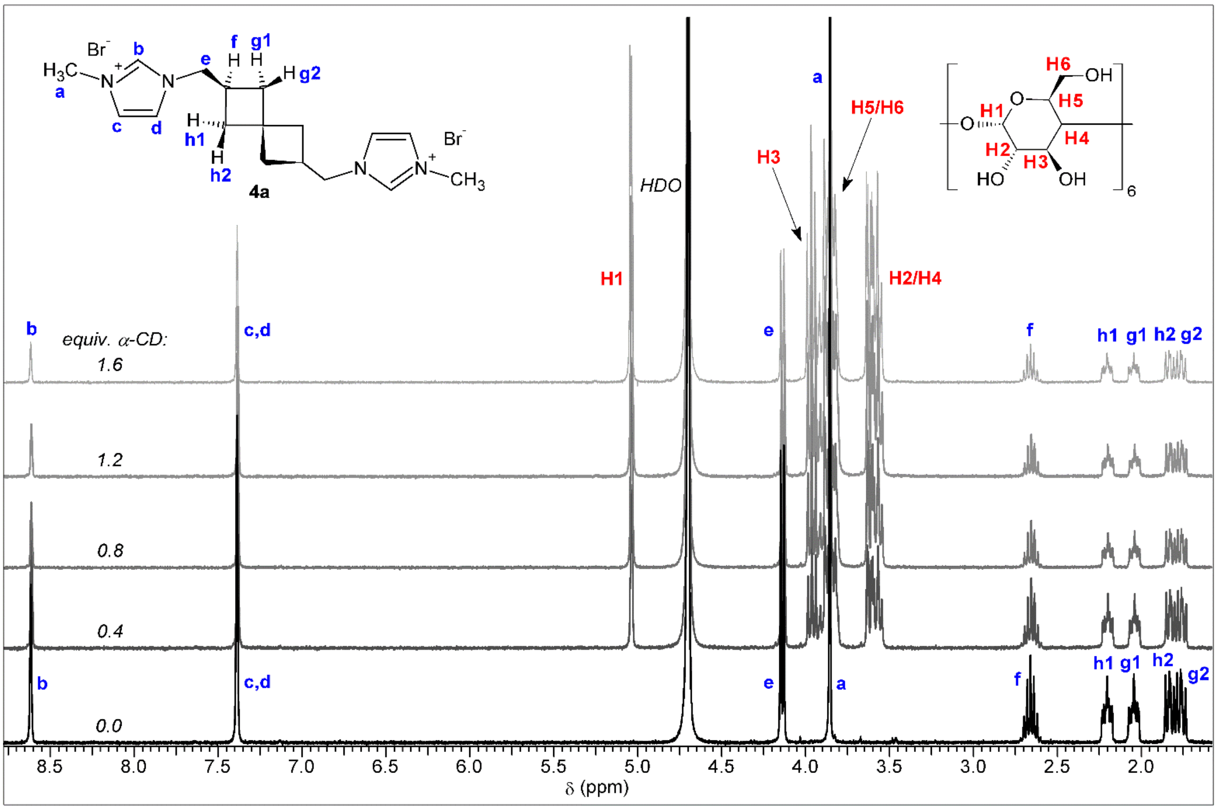

Figure S50 Stacking plot of ^{1}H NMR spectra (400 MHz) of mixtures of compound **4a** with α-CD in D_2O at 30 °C.

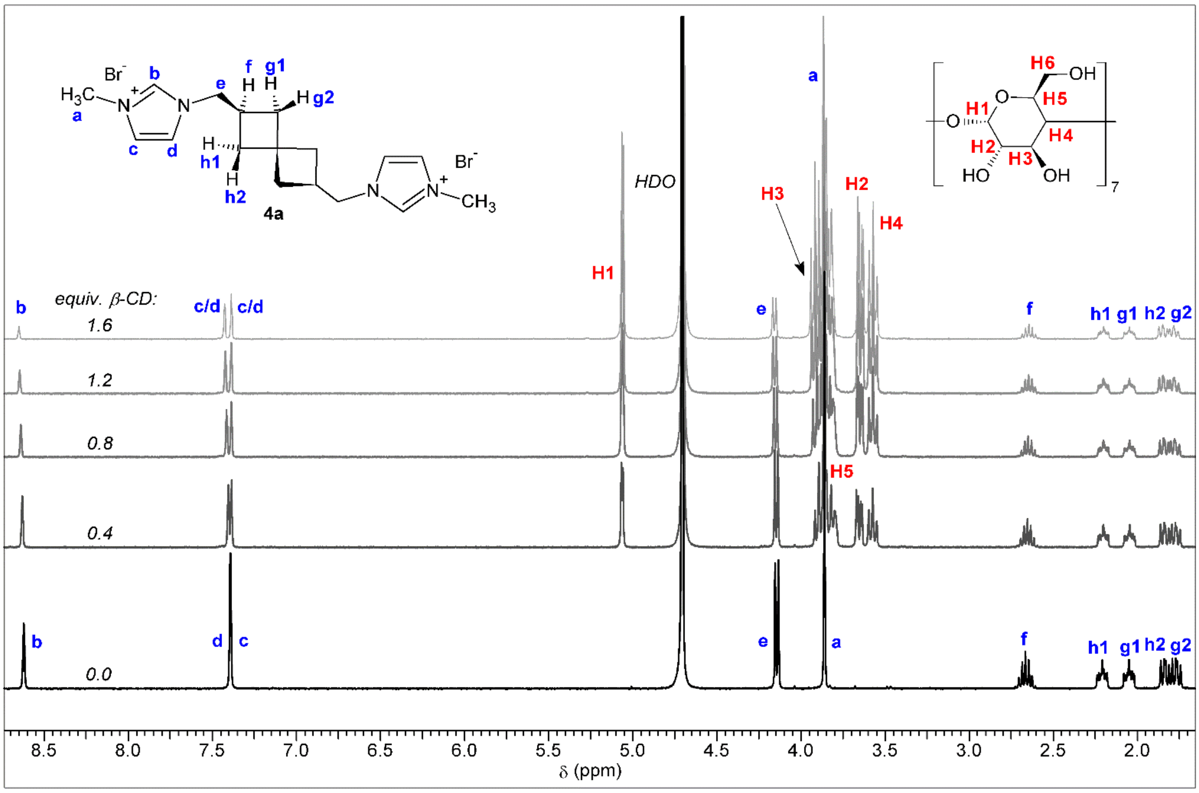

Figure S51 Stacking plot of ^{1}H NMR spectra (400 MHz) of mixtures of compound **4a** with β-CD in D_2O at 30 °C.

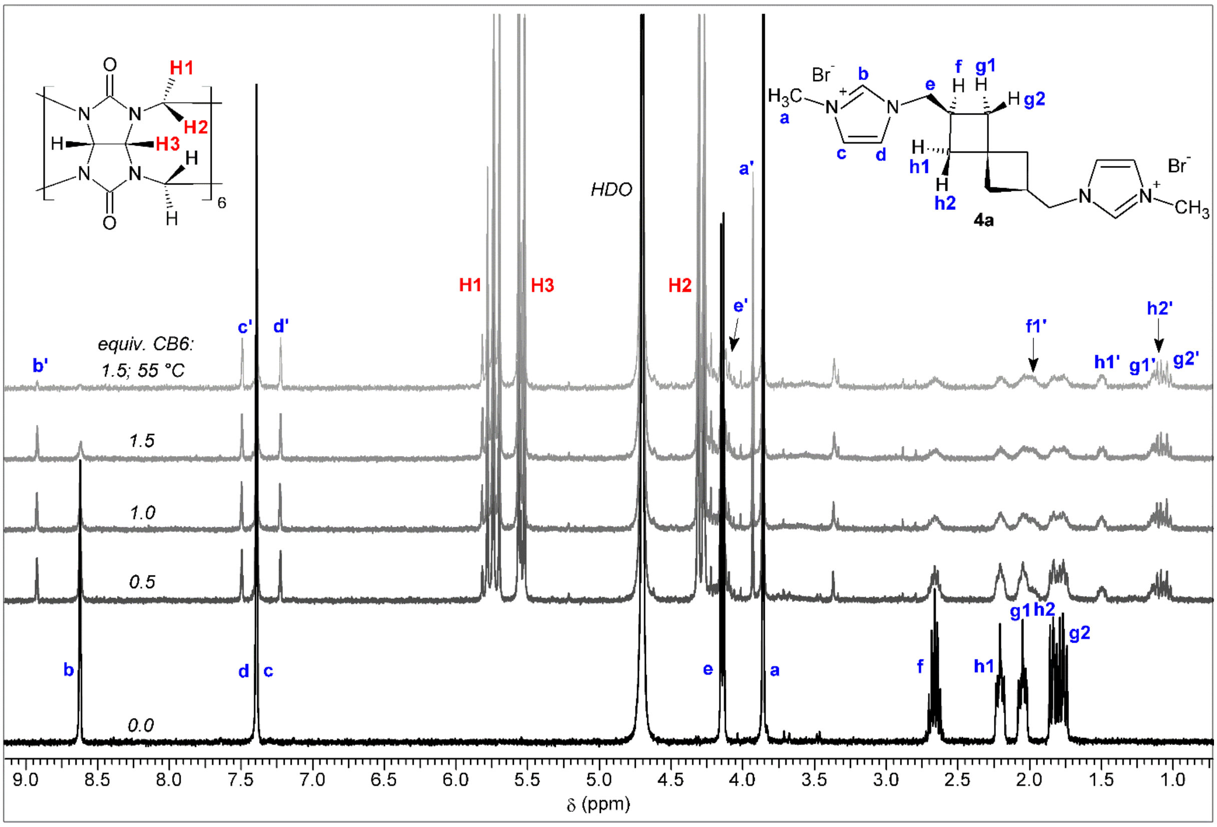

Figure S52 Stacking plot of ^{1}H NMR spectra (400 MHz) of mixtures of compound **4a** with CB6 in 50mM NaCl in D_2O at 30 °C.

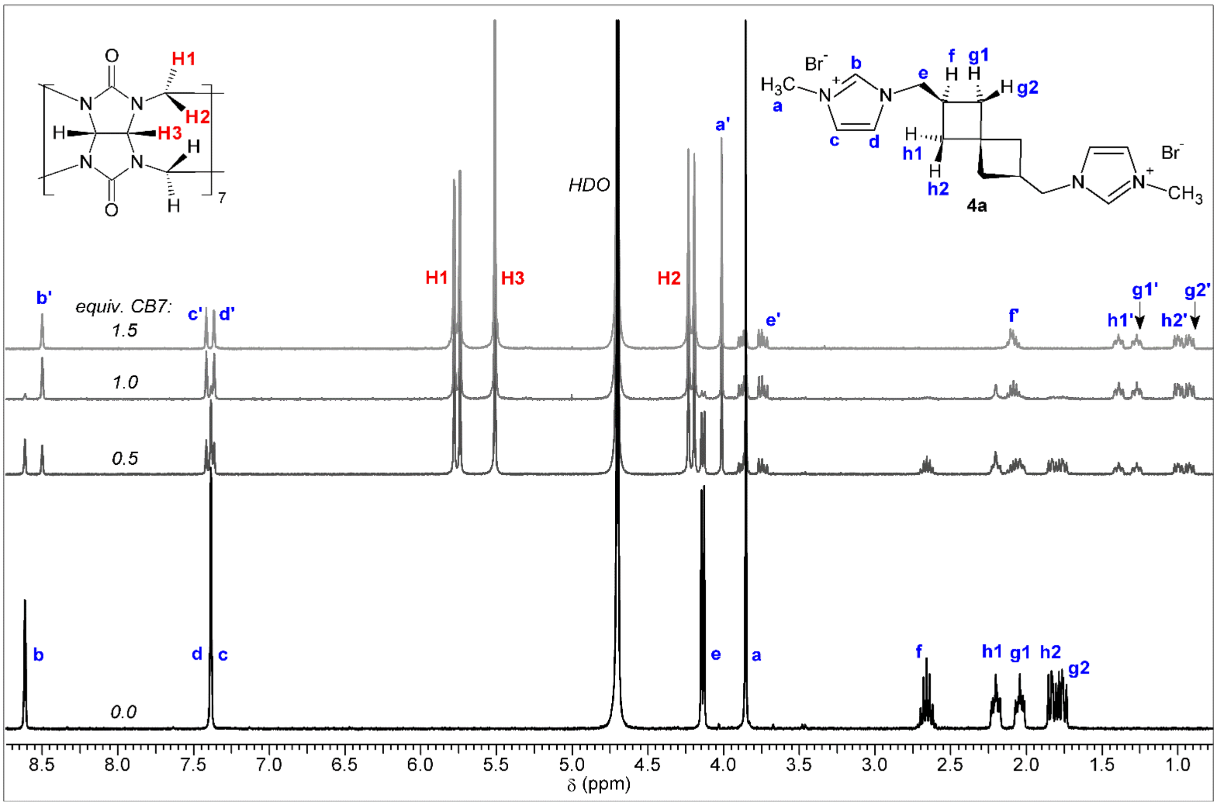

Figure S53 Stacking plot of ^{1}H NMR spectra (400 MHz) of mixtures of compound **4a** with CB7 in D_2O at 30 °C.

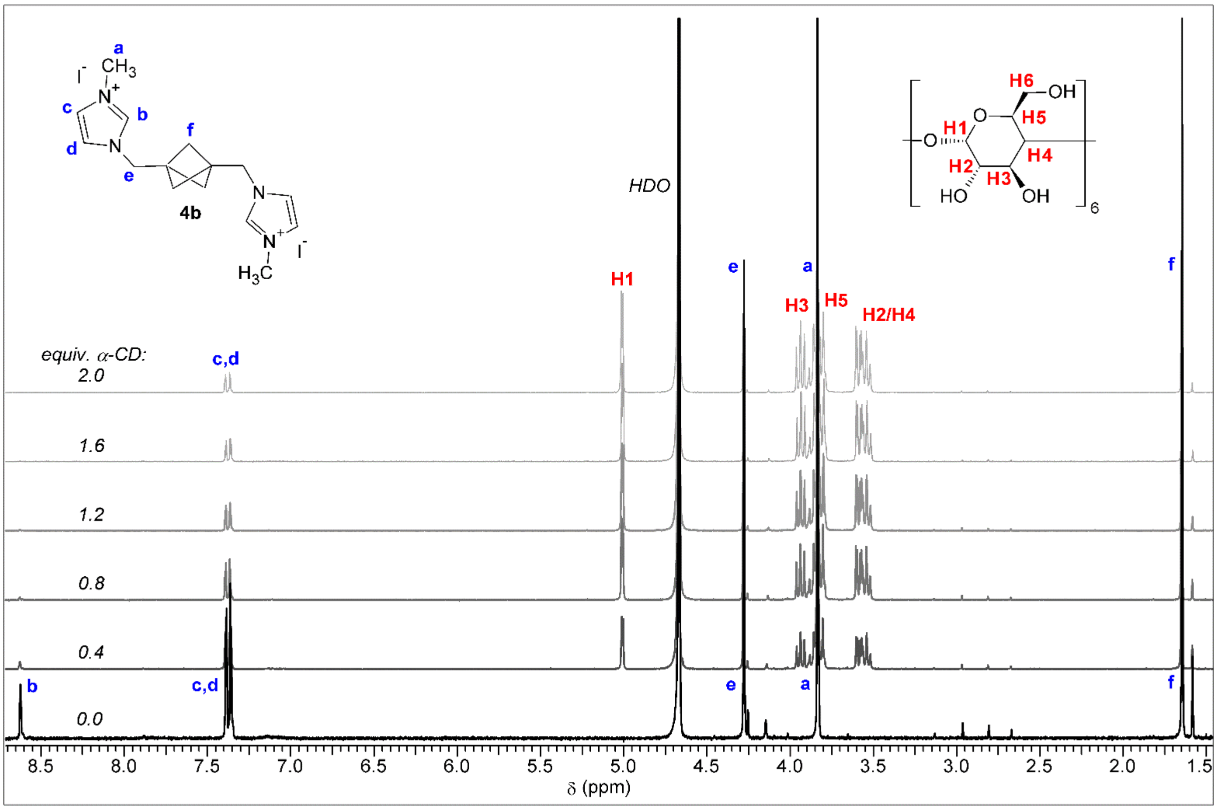

Figure S54 Stacking plot of ^{1}H NMR spectra (400 MHz) of mixtures of compound **4b** with α-CD in D_2O at 30 °C.

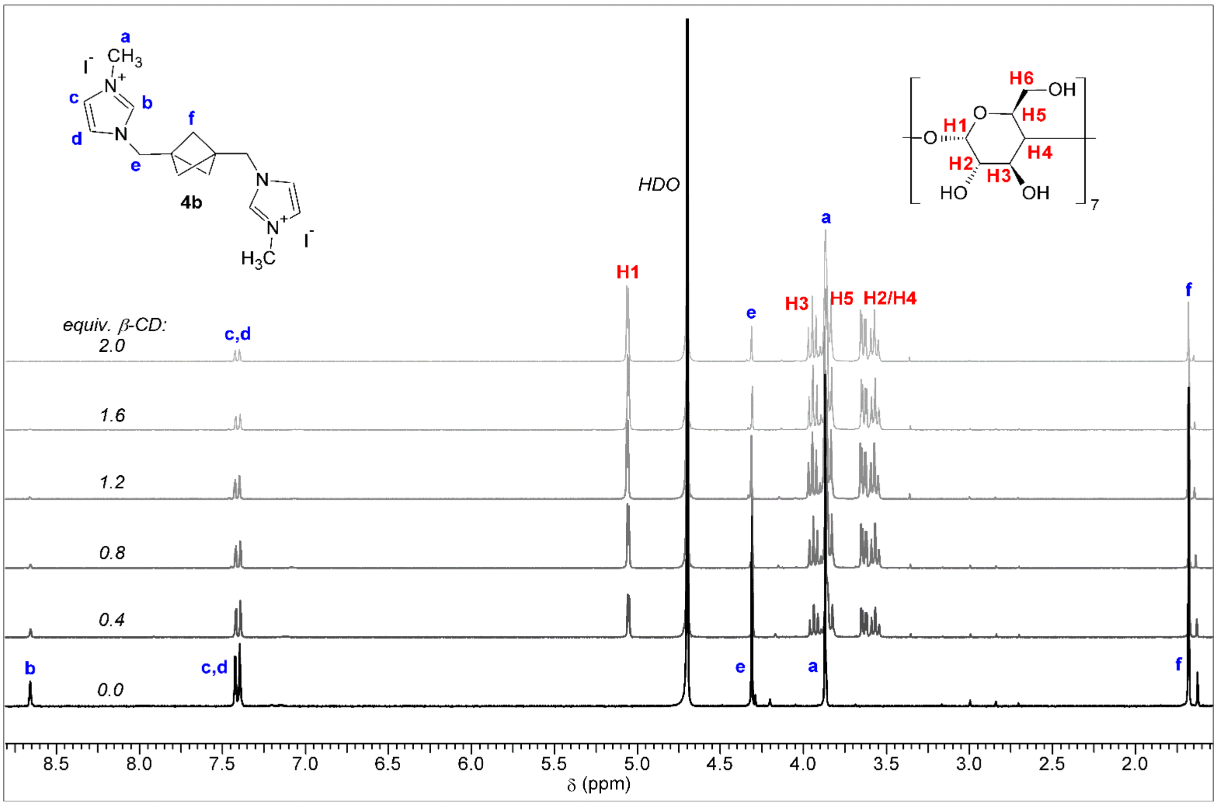

Figure S55 Stacking plot of ^{1}H NMR spectra (400 MHz) of mixtures of compound **4b** with β-CD in D_2O at 30 °C.

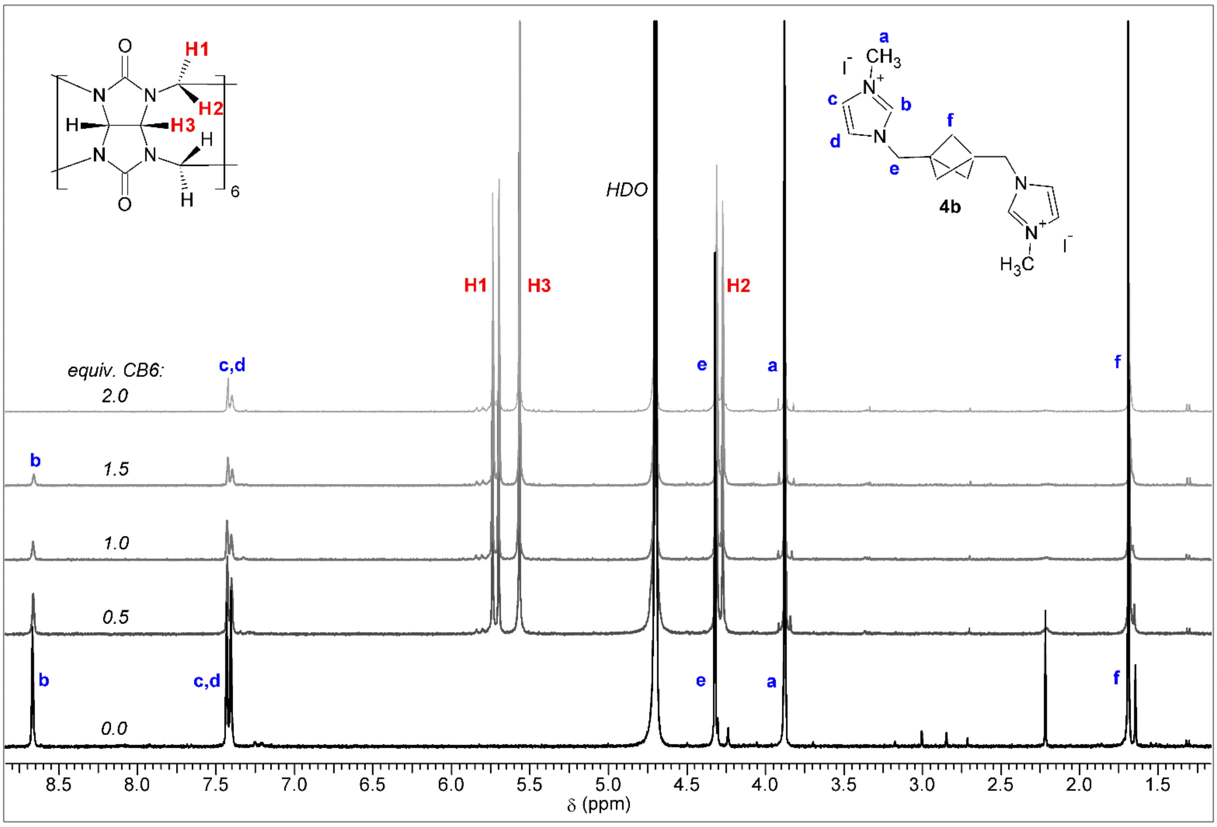

Figure S56 Stacking plot of ^{1}H NMR spectra (400 MHz) of mixtures of compound **4b** with CB6 in 50mM NaCl in D_2O
at 30 °C.

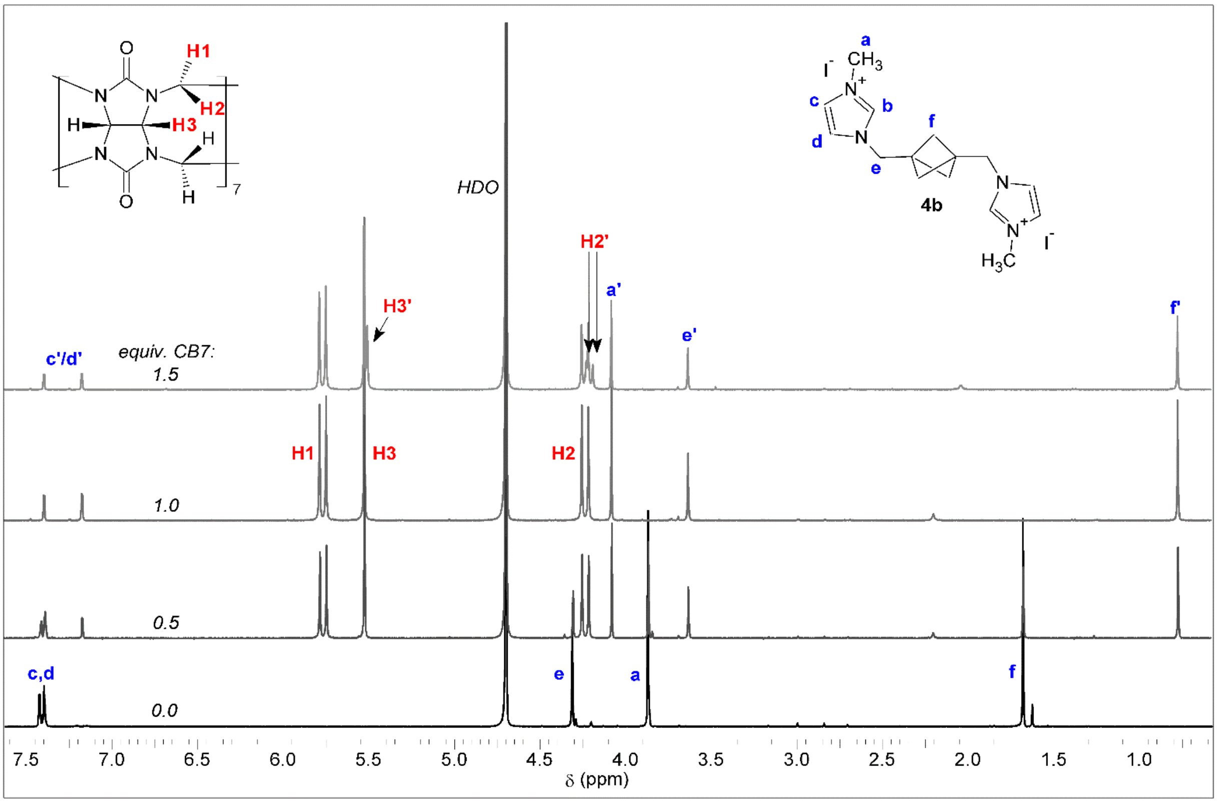

Figure S57 Stacking plot of ^{1}H NMR spectra (400 MHz) of mixtures of compound **4b** with CB7 in D_2O
at 30 °C.

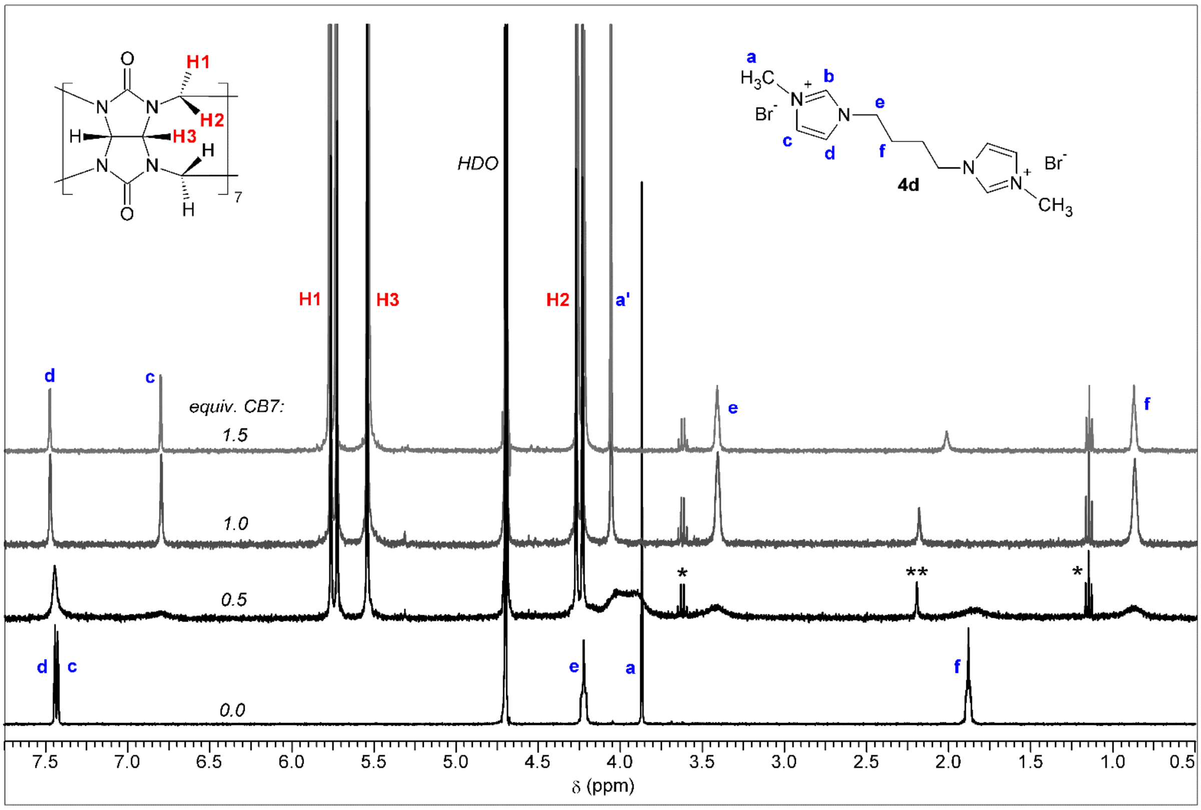

Figure S58 Stacking plot of ^{1}H NMR spectra (400 MHz) of mixtures of compound **4b** with CB7 in D_2O at 30 °C.

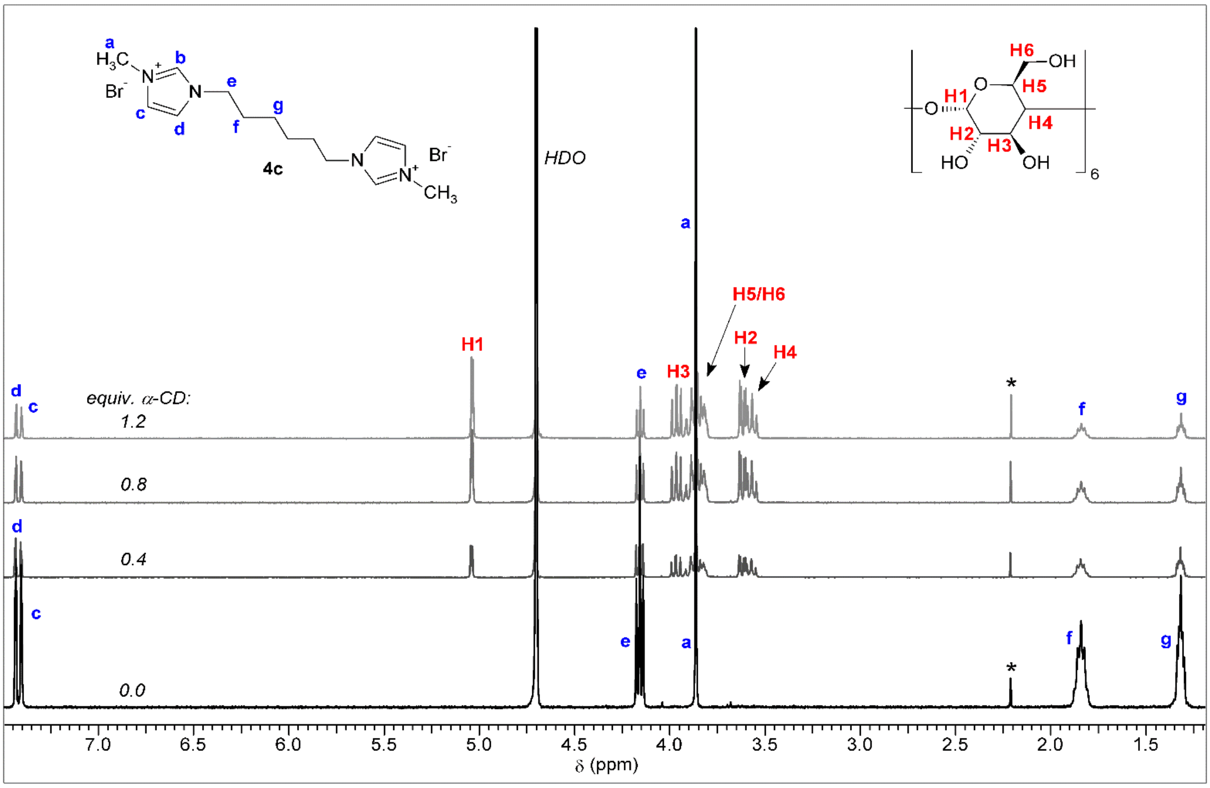

Figure S59 Stacking plot of ^{1}H NMR spectra (400 MHz) of mixtures of compound **4c** with α-CD in D_2O at 30 °C. "*" a signal of an impurity

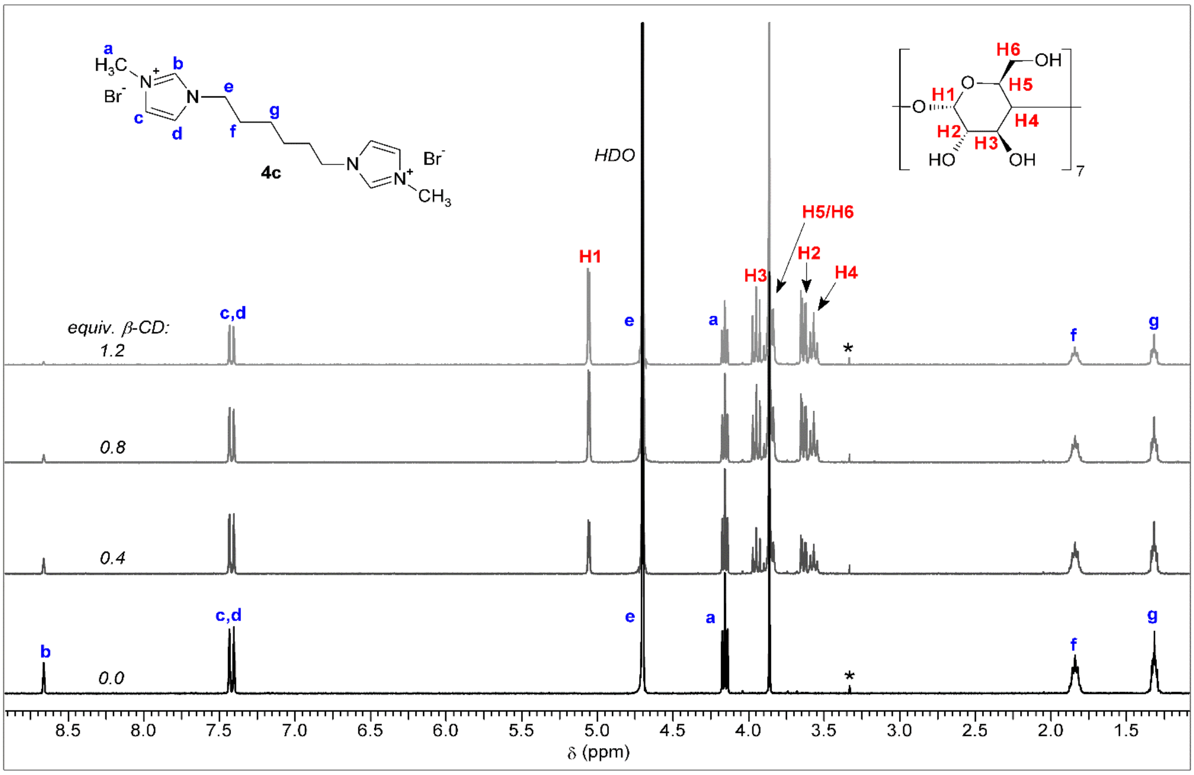

Figure S60 Stacking plot of ^{1}H NMR spectra (400 MHz) of mixtures of compound **4c** with β-CD in D_2O at 30 °C. "*" a signal of an impurity

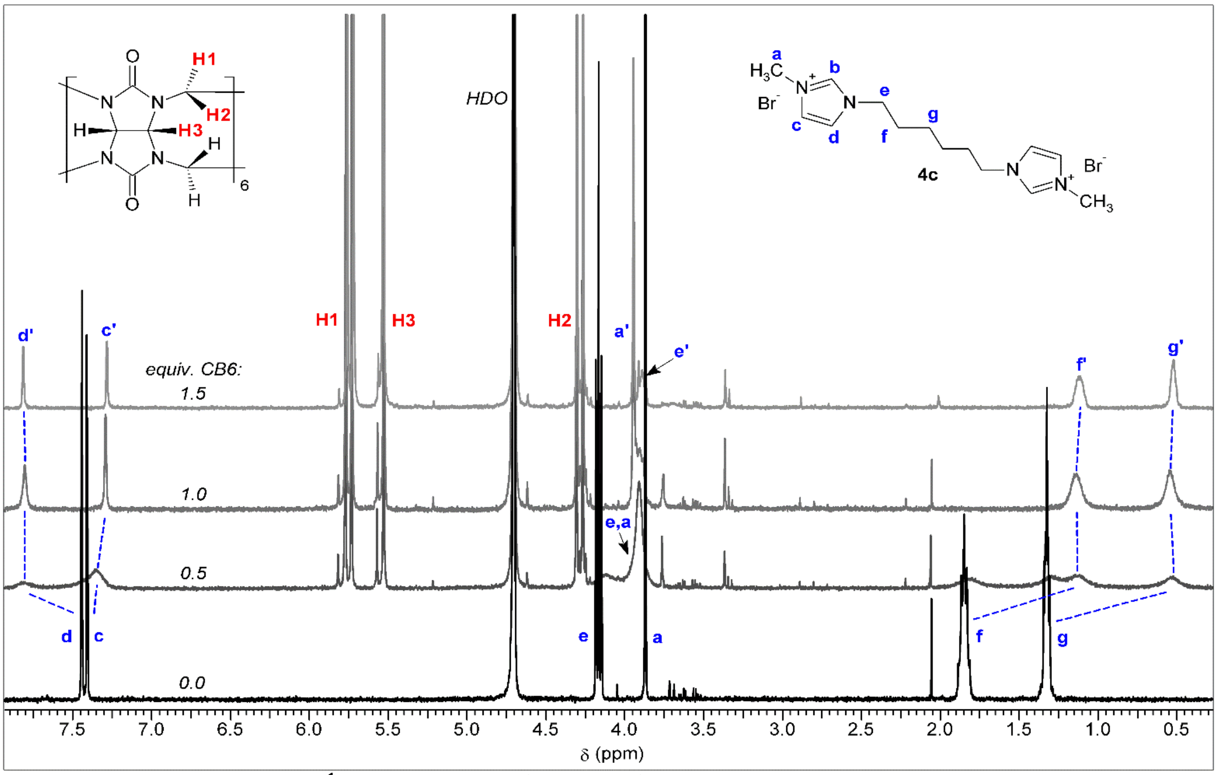

Figure S61 Stacking plot of ^{1}H NMR spectra (400 MHz) of mixtures of compound **4c** with CB6 in 50mM NaCl in D_2O at 30 °C.

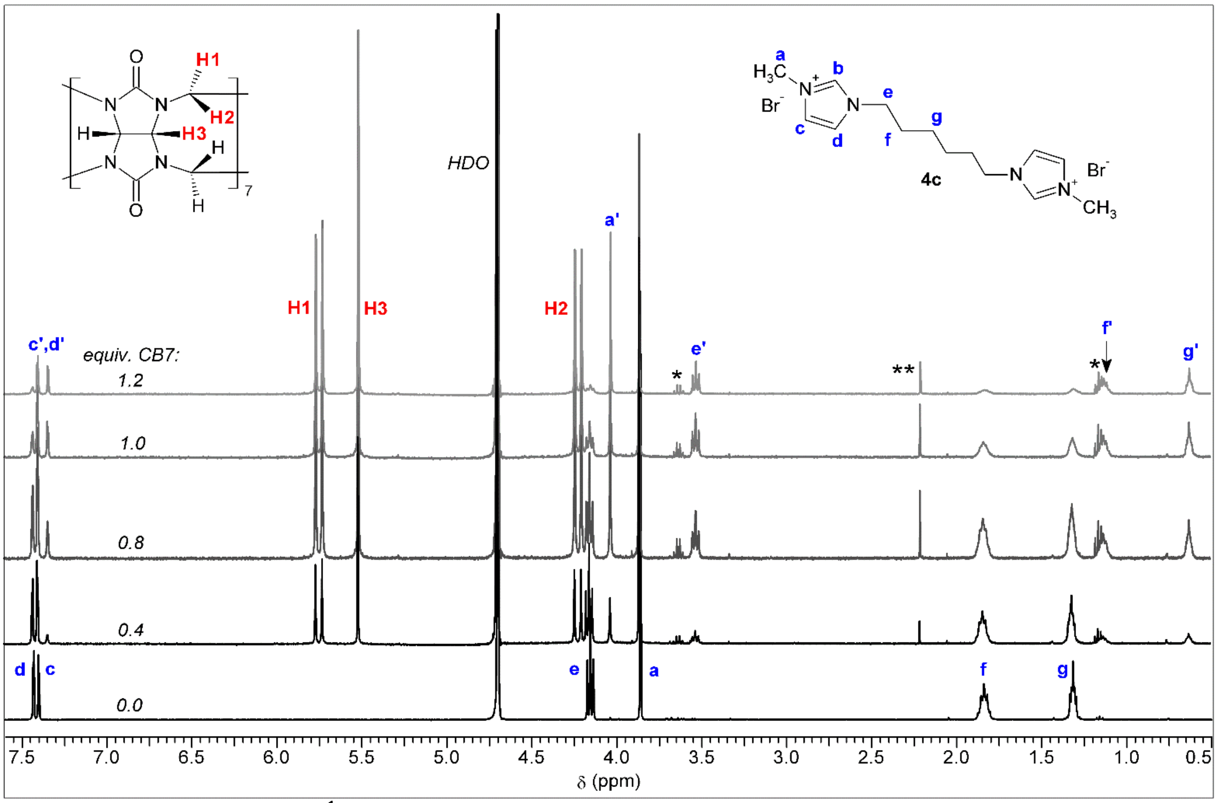

Figure S62 Stacking plot of ^{1}H NMR spectra (400 MHz) of mixtures of compound **4c** with CB7 in D_2O at 30 °C. "⋆" a residual ethanol from commercial CB7; "⋆⋆" a residual acetone from commercial CB7.

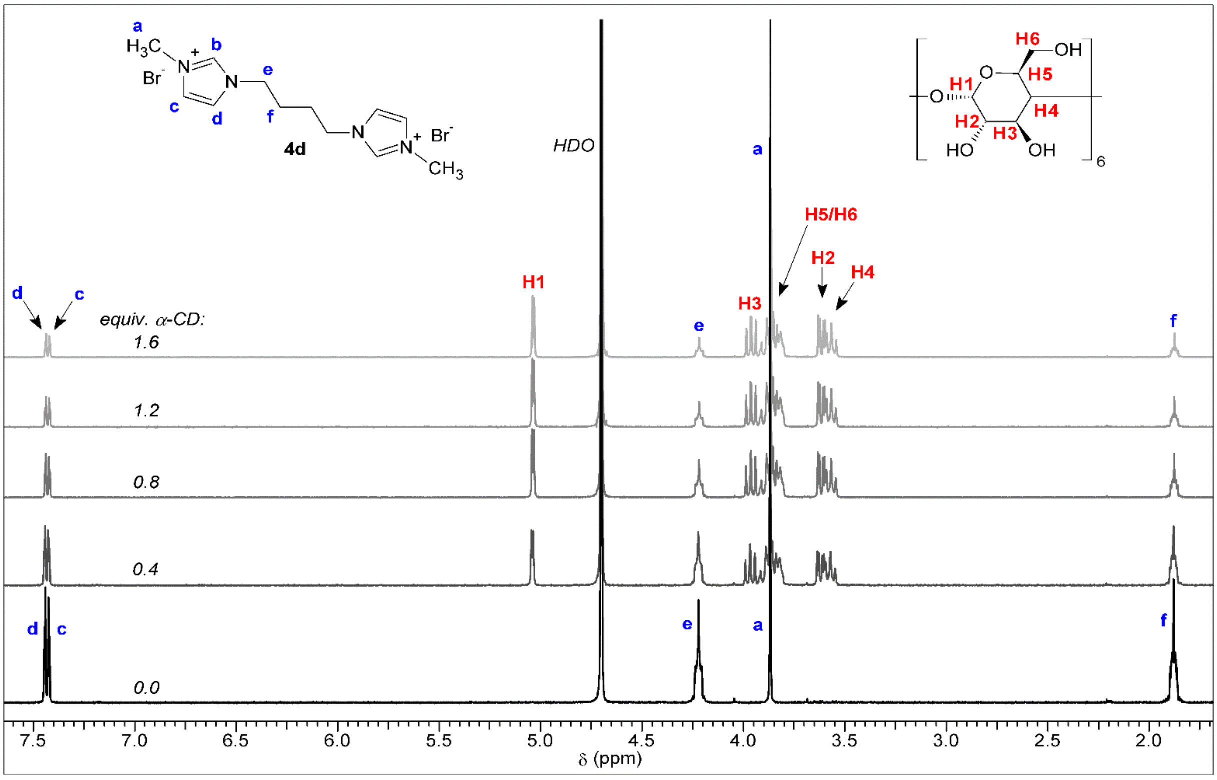

Figure S63 Stacking plot of ^{1}H NMR spectra (400 MHz) of mixtures of compound **4d** with α-CD in D_2O at 30 °C.

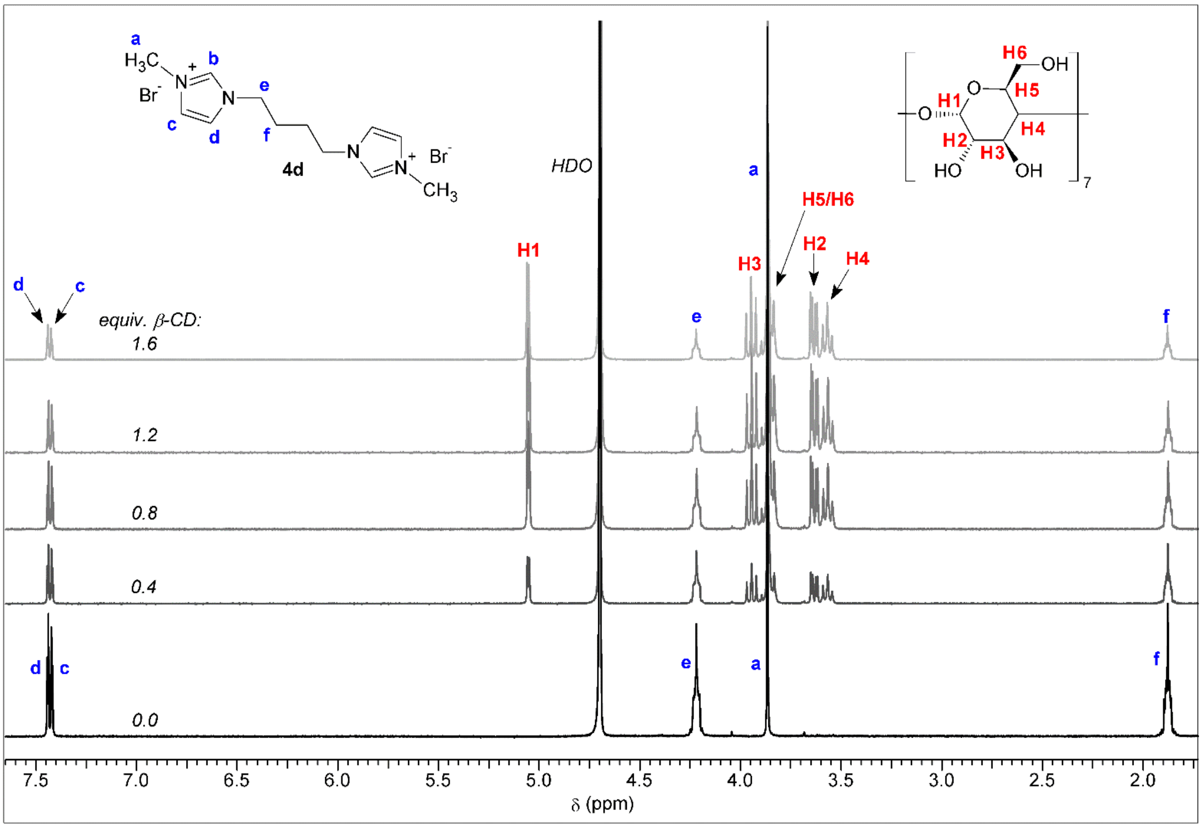

Figure S64 Stacking plot of ^{1}H NMR spectra (400 MHz) of mixtures of compound **4d** with β-CD in D_2O at 30 °C.

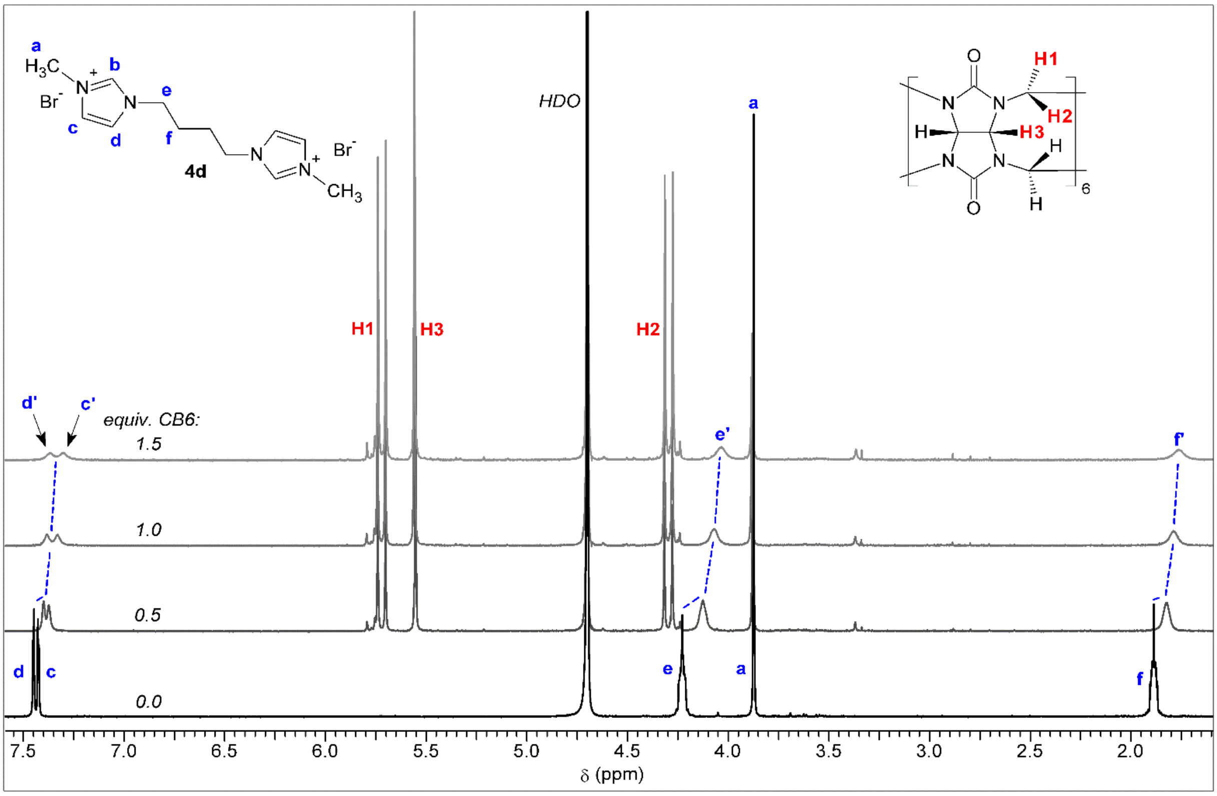

Figure S65 Stacking plot of ^{1}H NMR spectra (400 MHz) of mixtures of compound **4d** with CB6 in 50mM NaCl in D_2O at 30 °C.

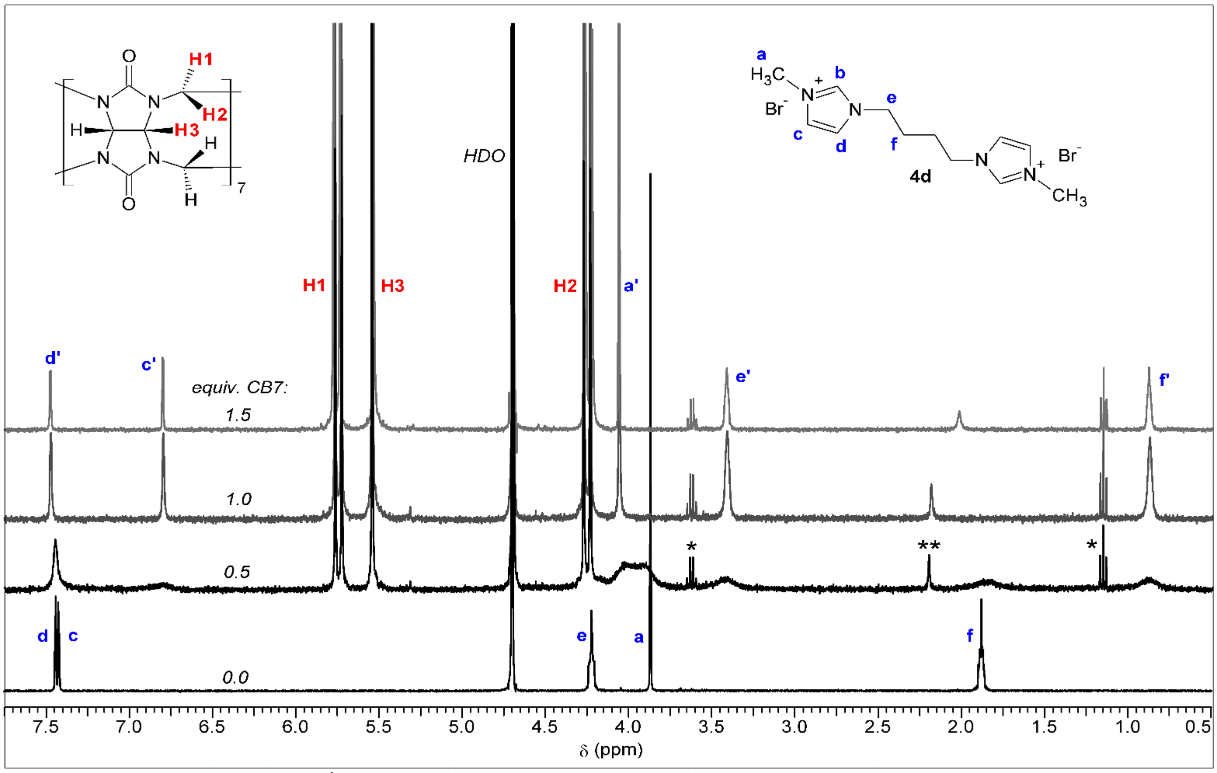

Figure S66 Stacking plot of ^{1}H NMR spectra (400 MHz) of mixtures of compound **4d** with CB7 in D_2O at 30 °C. "⋆" a residual ethanol from commercial CB7; "⋆⋆" a residual acetone from commercial CB7.

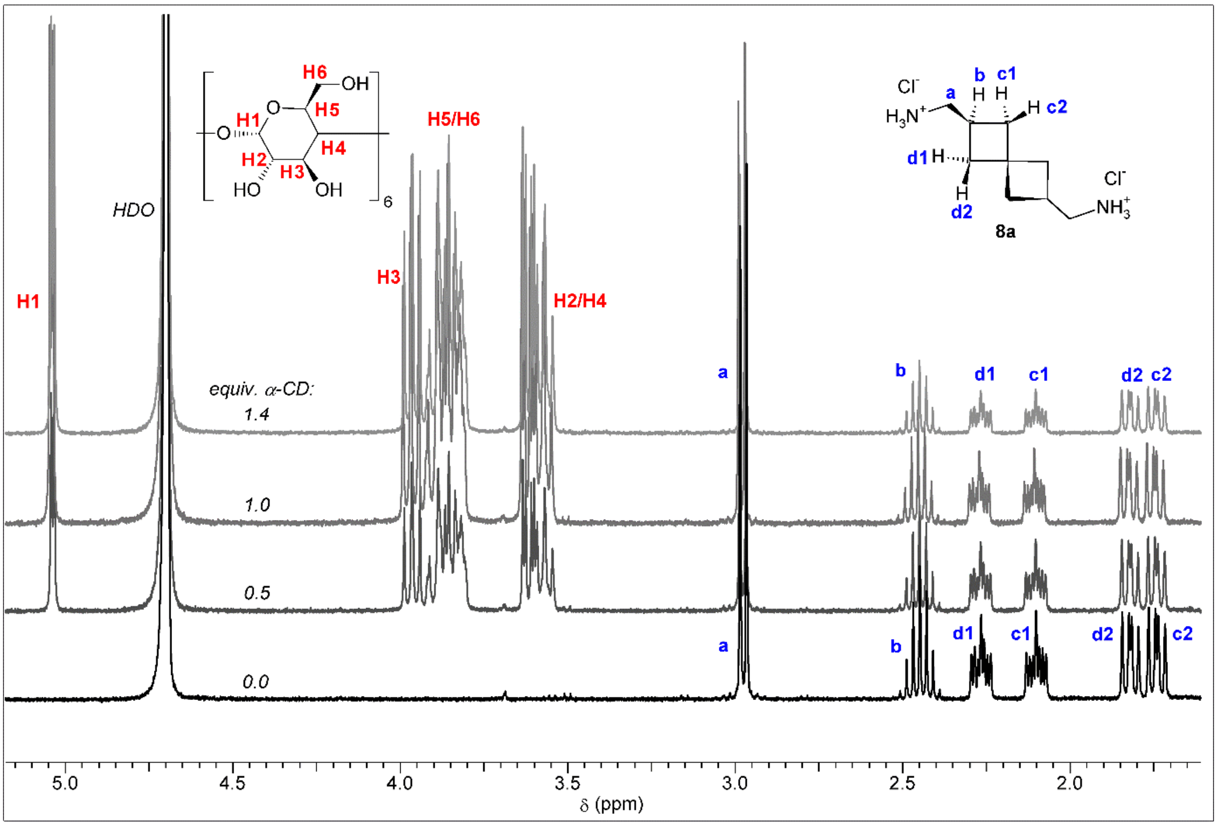

Figure S67 Stacking plot of ^{1}H NMR spectra (400 MHz) of mixtures of compound **8a** with α-CD in D_2O at 30 °C.

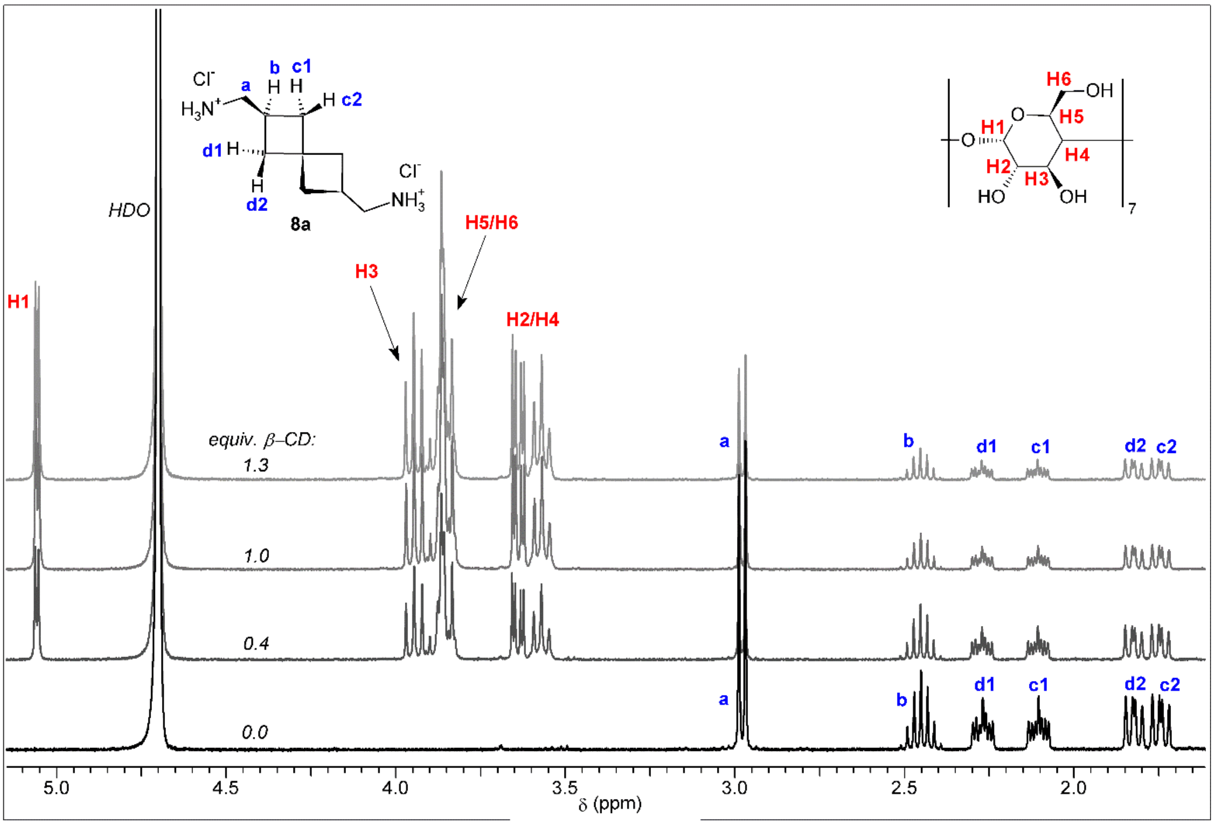

Figure S68 Stacking plot of ^{1}H NMR spectra (400 MHz) of mixtures of compound **8a** with β-CD in D_2O at 30 °C.

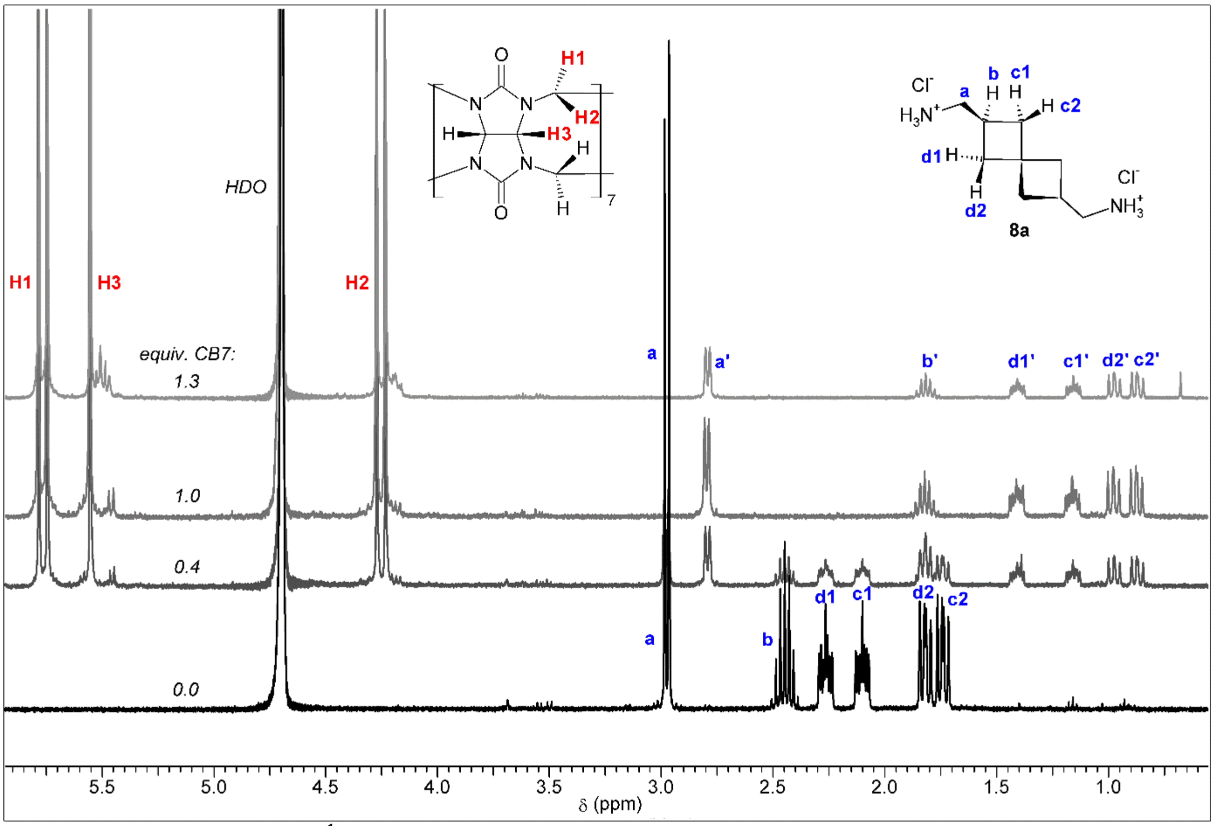

Figure S69 Stacking plot of ^{1}H NMR spectra (400 MHz) of mixtures of compound **8a** with CB7 in D_2O at 30 °C.

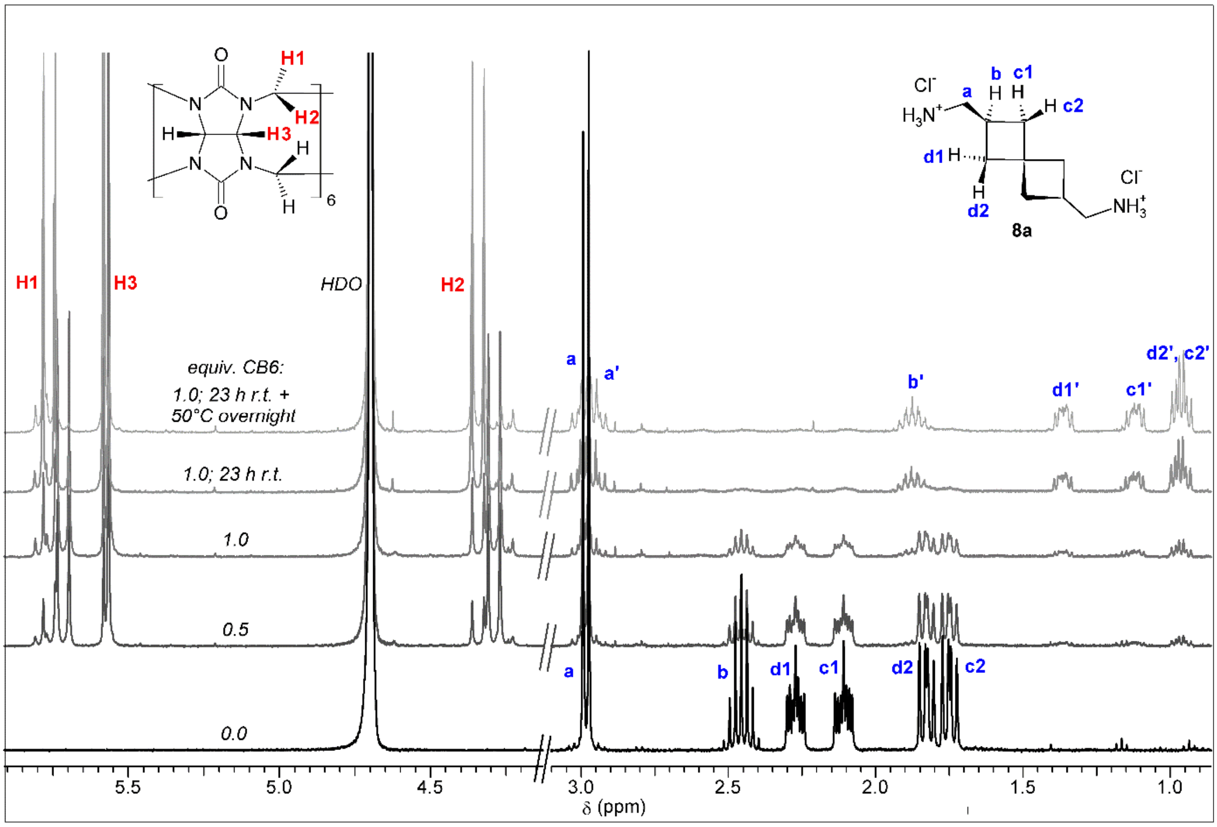

Figure S70 Stacking plot of ^{1}H NMR spectra (400 MHz) of mixtures of compound **8a** with CB6 in 50 mM NaCl in D_2O.

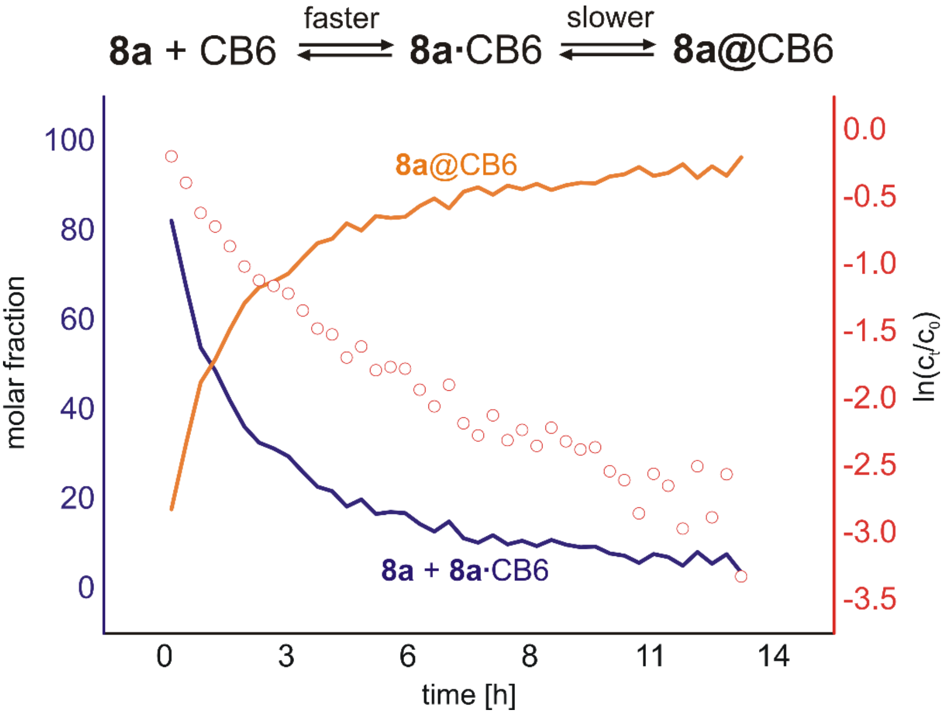

Figure S71 Molar fractions (integration of ^{1}H NMR spectra recorded in 50 mM NaCl in D_2O at 303 K) of inclusion complex **8a**@CB6 (orange line) and other forms of **8a** (blue line) and first-order linearisation (red circles) plotted against time.

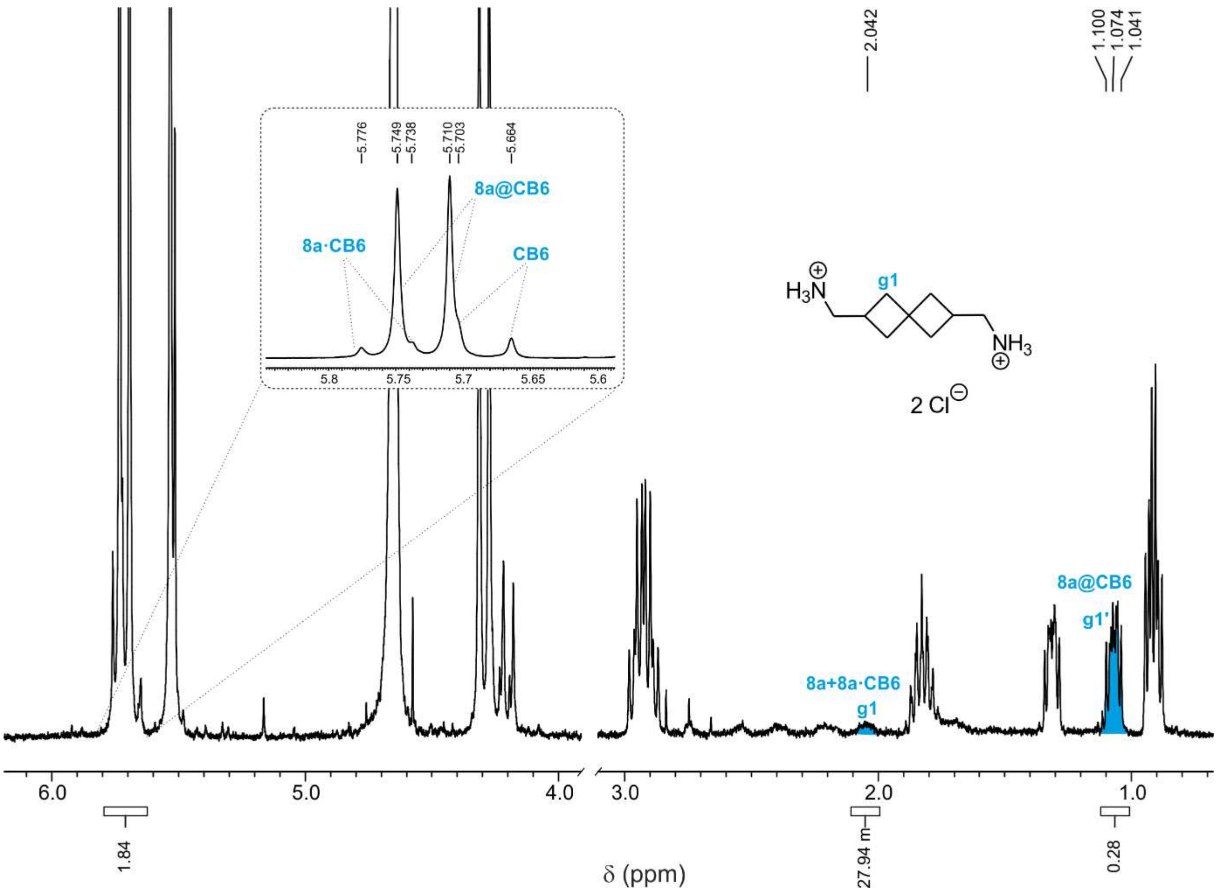

Figure S72 An example of ^{1}H NMR (50 mM NaCl in D_2O, 303 K, 400 MHz) spectrum used for **8a**@CB6 association constant determination. The CB6 signals assignment is based on changes in intensities during the titration experiment (Figure S70). The concentrations of particular forms of CB6 were determined after deconvolution (using Delta software) of the clusters of CB6 signals.

Mass spectra of the guests and complexes

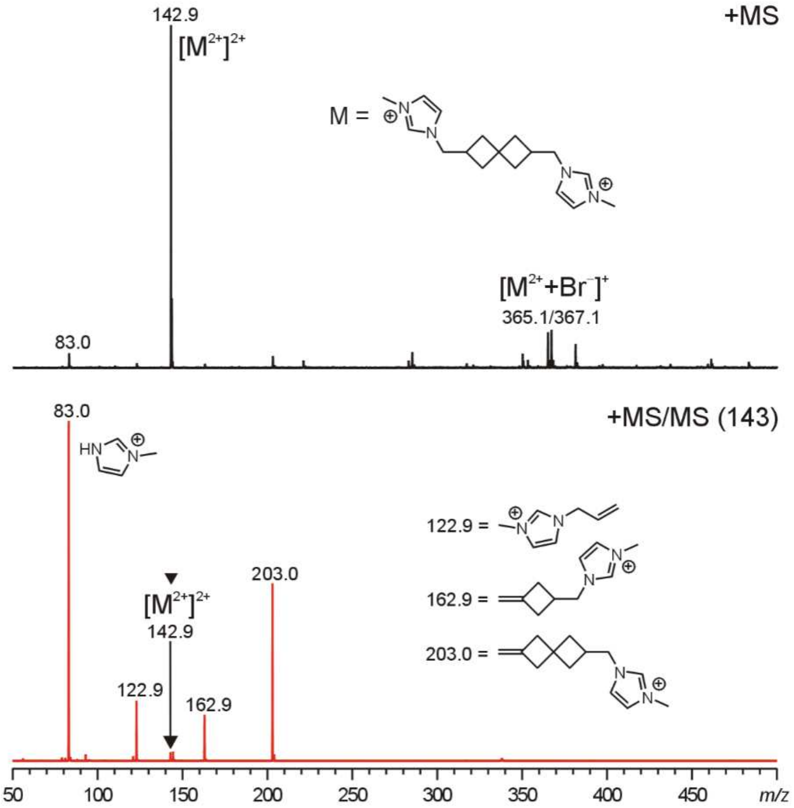

Figure S73 Mass spectrum of the guest **4a**.

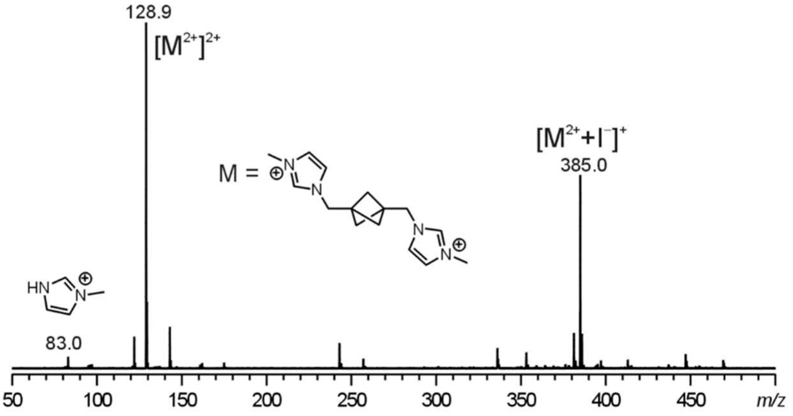

Figure S74 Mass spectrum of the guest **4b**.

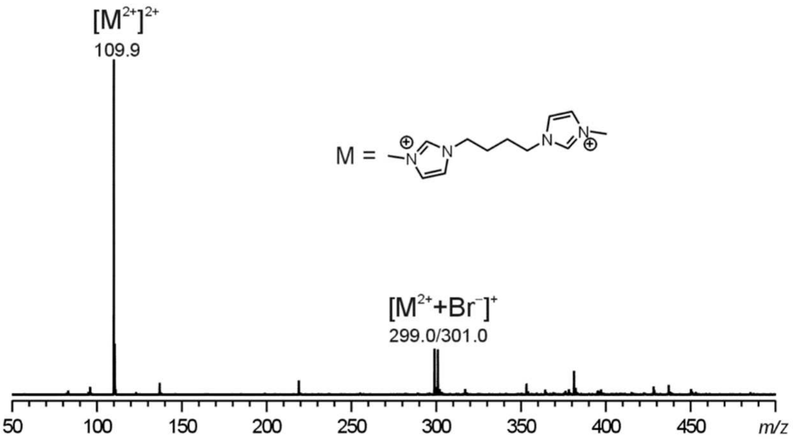

Figure S75 Mass spectrum of the guest **4c**.

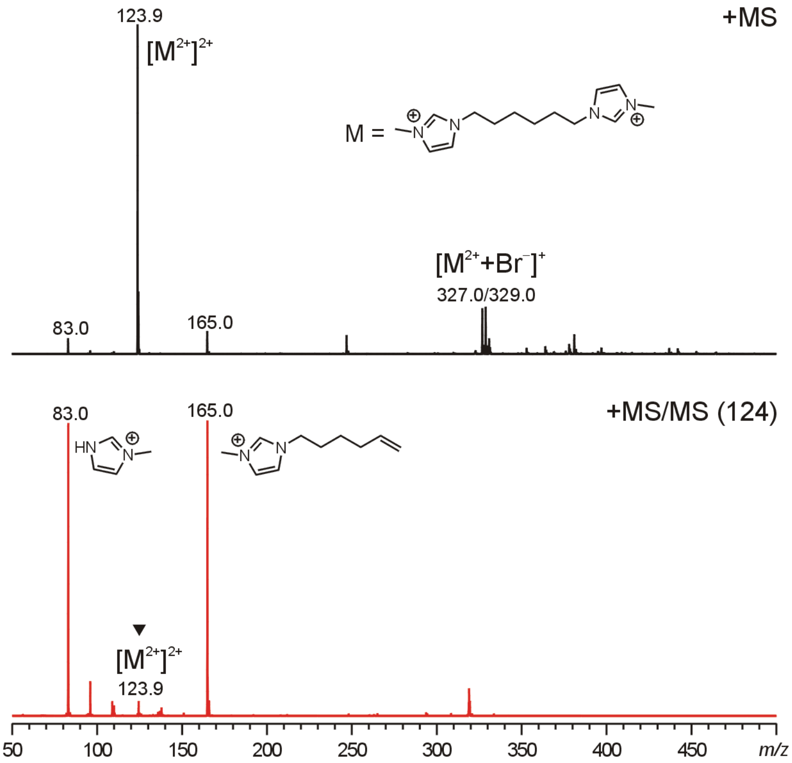

Figure S76 Mass spectrum of the guest **4d**.

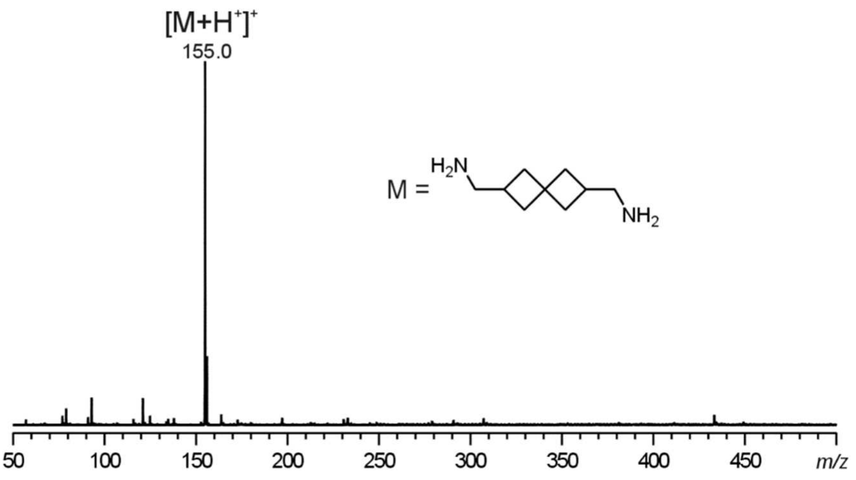

Figure S77 Mass spectrum of the guest **8a**.

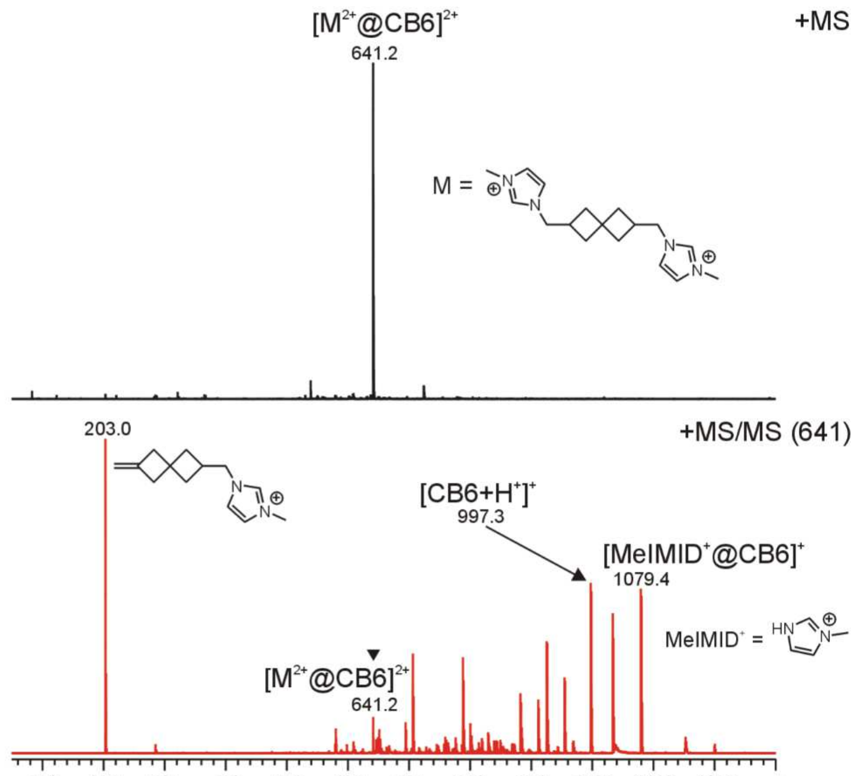

Figure S78 Mass spectrum of a mixture of the guest **4a** and CB6.

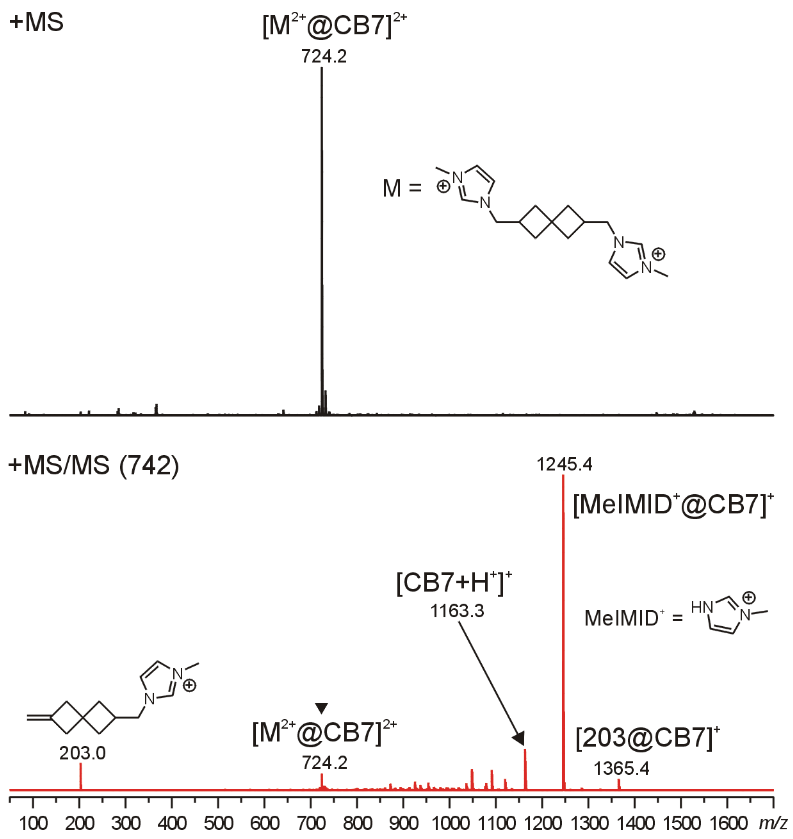

Figure S79 Mass spectrum of a mixture of the guest **4a** and CB7.

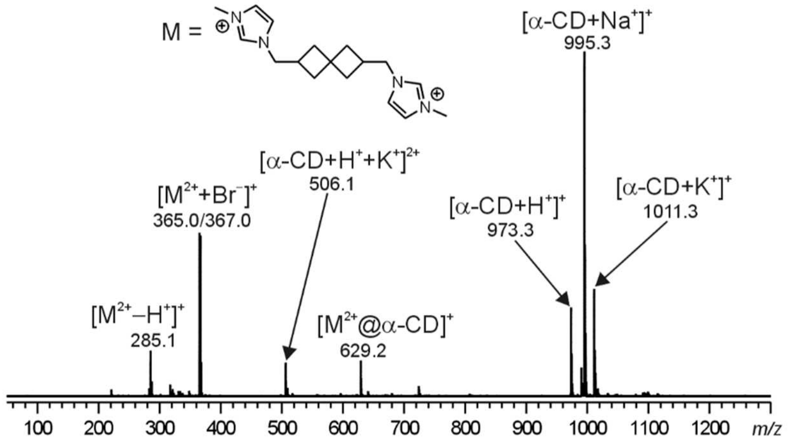

Figure S80 Mass spectrum of a mixture of the guest **4a** and α-CD.

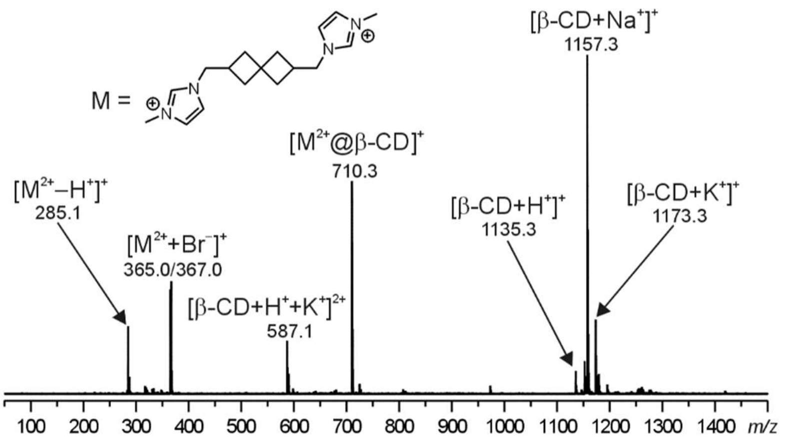

Figure S81 Mass spectrum of a mixture of the guest **4a** and β-CD.

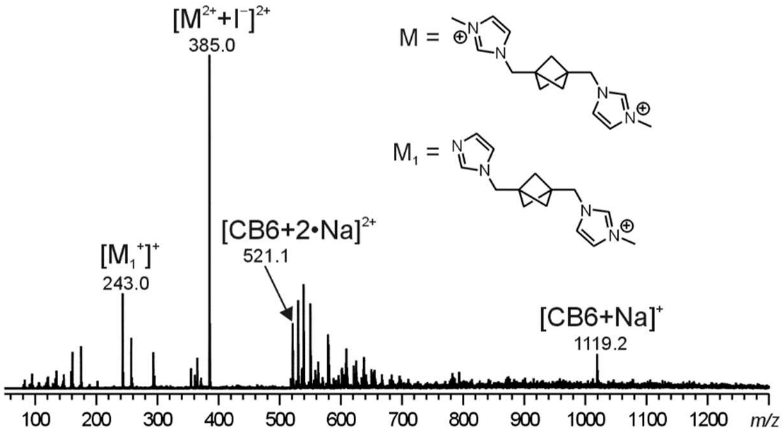

Figure S82 Mass spectrum of a mixture of the guest **4b** and CB6.

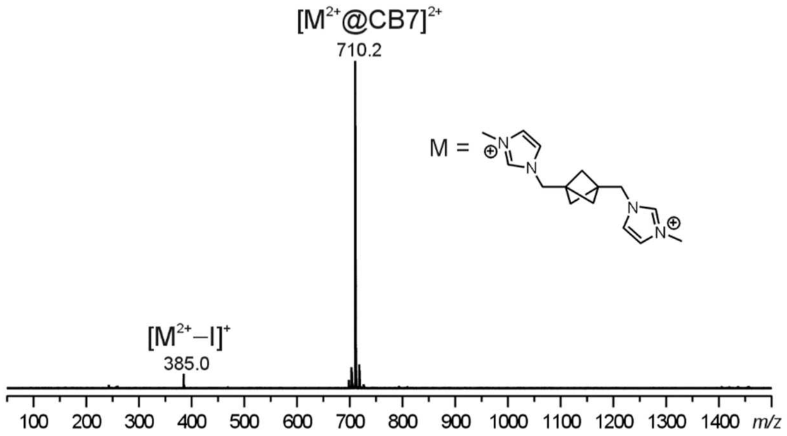

Figure S83 Mass spectrum of a mixture of the guest **4b** and CB7.

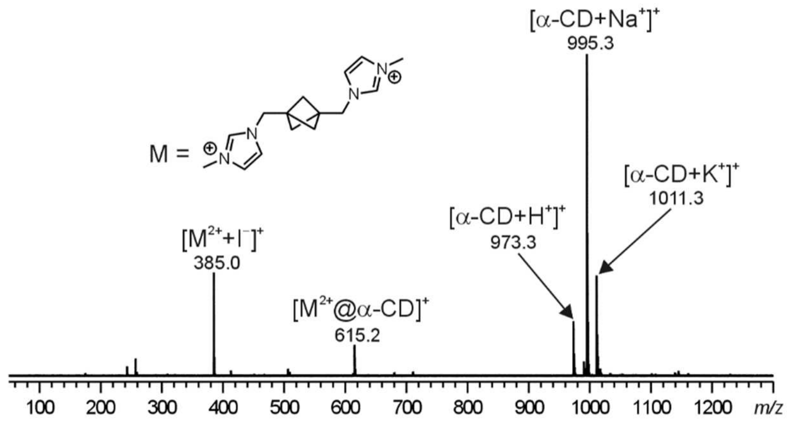

Figure S84 Mass spectrum of a mixture of the guest **4b** and α-CD.

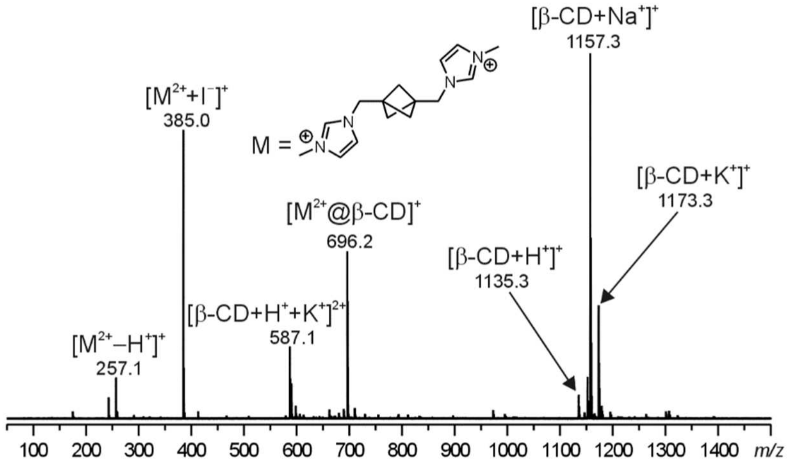

Figure S85 Mass spectrum of a mixture of the guest **4b** and β-CD.

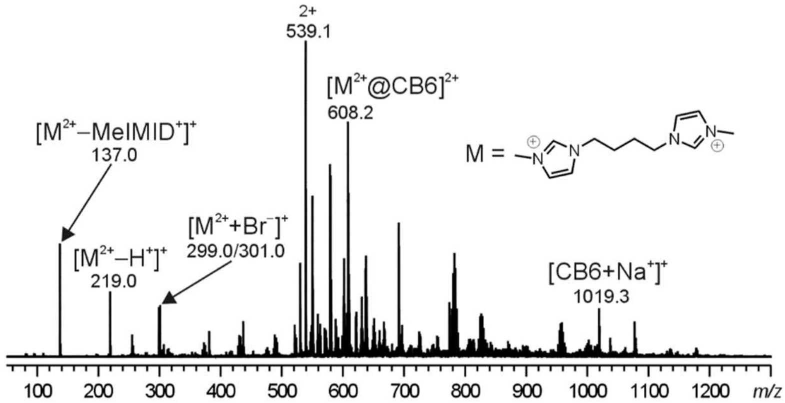

Figure S86 Mass spectrum of a mixture of the guest **4c** and CB6.

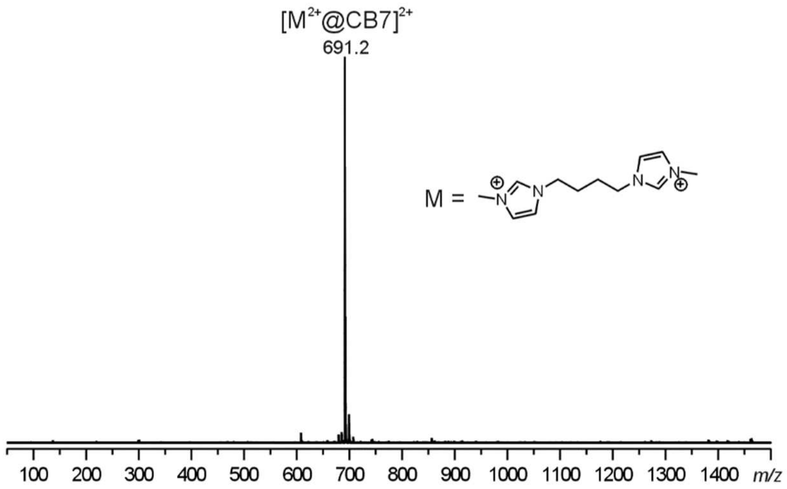

Figure S87 Mass spectrum of a mixture of the guest **4c** and CB7.

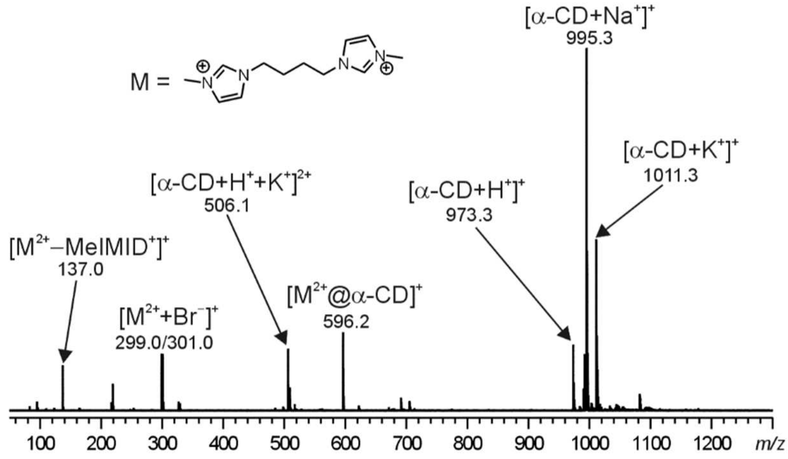

Figure S88 Mass spectrum of a mixture of the guest **4c** and α-CD.

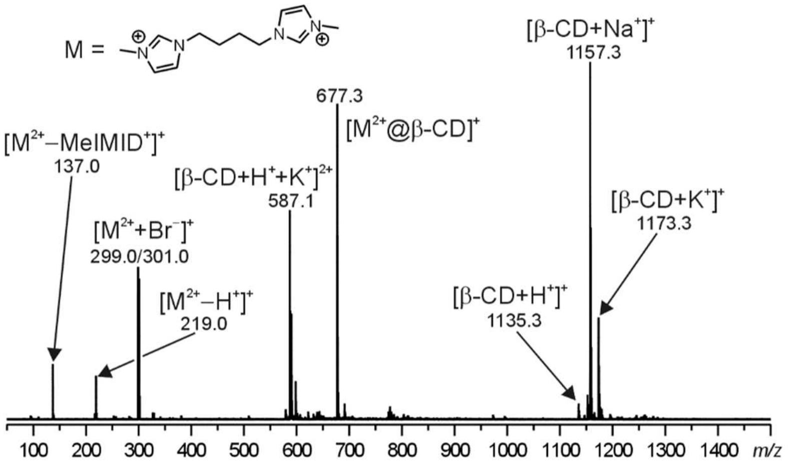

Figure S89 Mass spectrum of a mixture of the guest **4c** and β-CD.

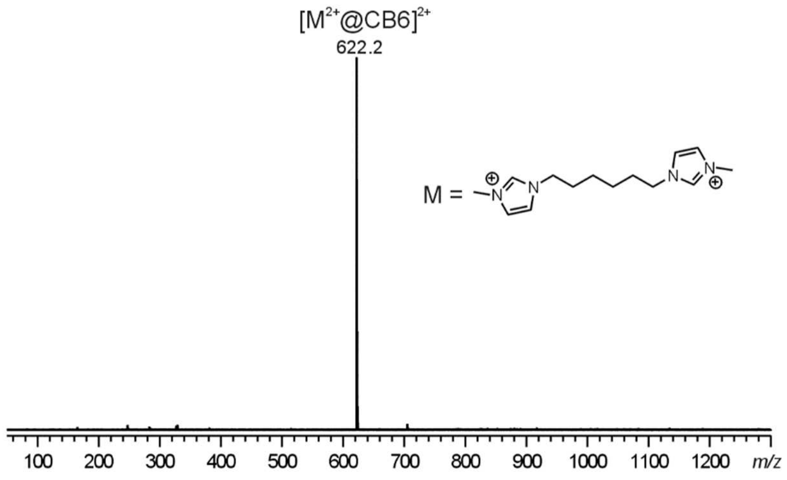

Figure S90 Mass spectrum of a mixture of the guest **4d** and CB6.

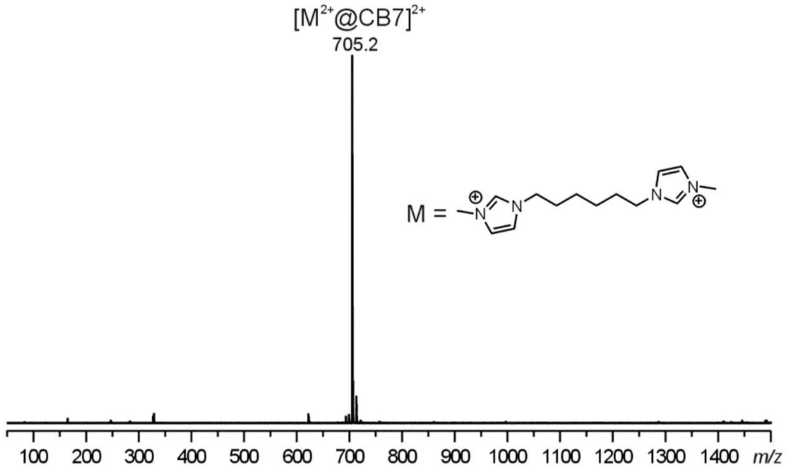

Figure S91 Mass spectrum of a mixture of the guest **4d** and CB7.

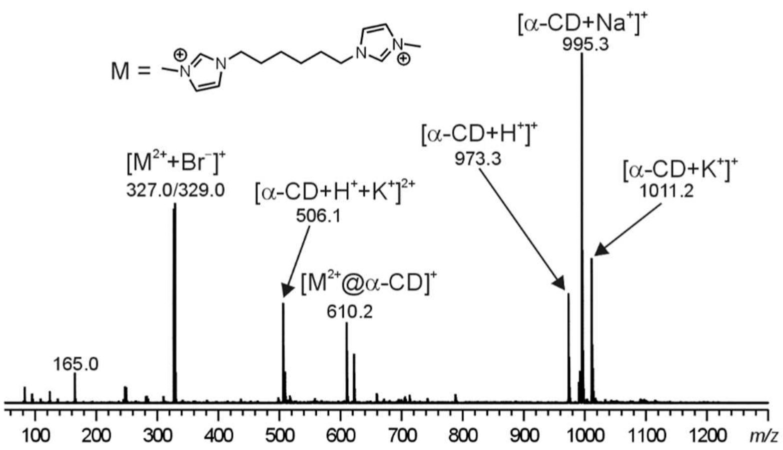

Figure S92 Mass spectrum of a mixture of the guest **4d** and α-CD.

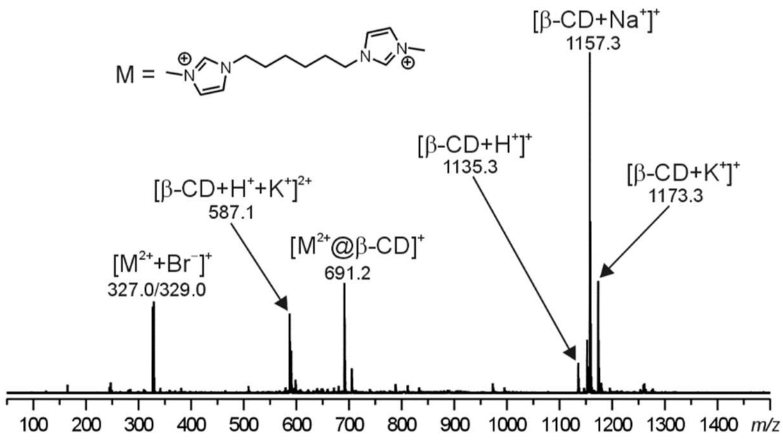

Figure S93 Mass spectrum of a mixture of the guest **4d** and β-CD.

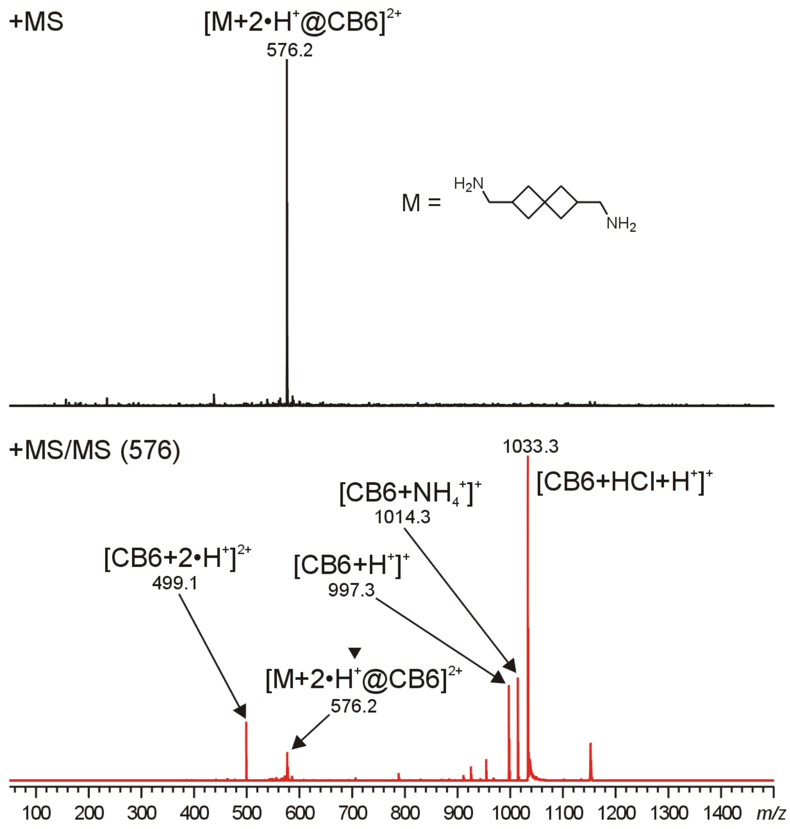

Figure S94 Mass spectrum of a mixture of the guest **8a** and CB6.

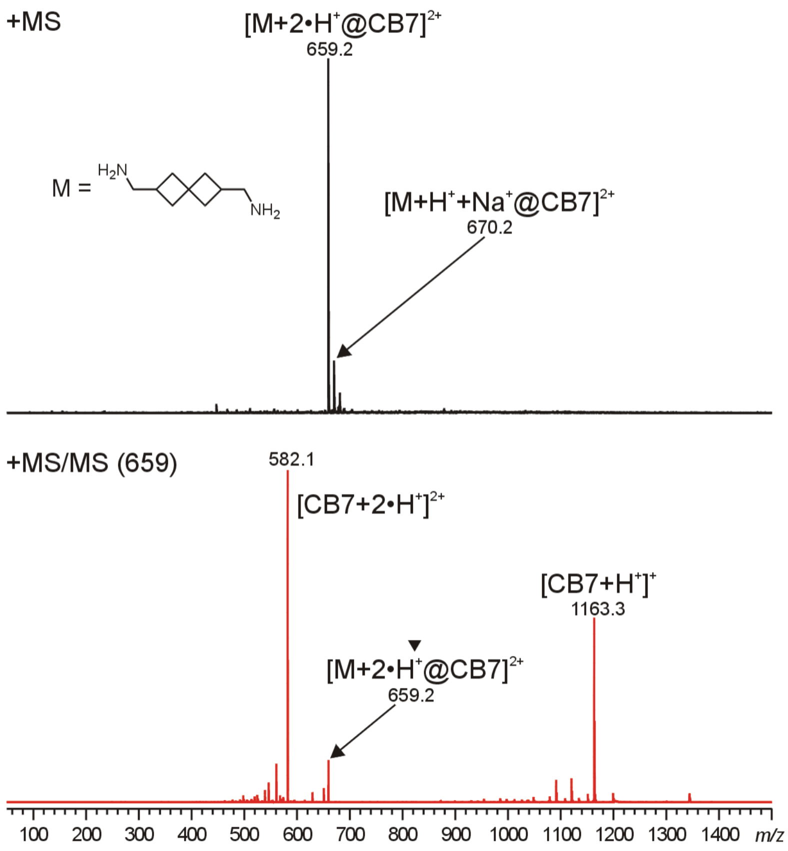

Figure S95 Mass spectrum of a mixture of the guest **8a** and CB7.

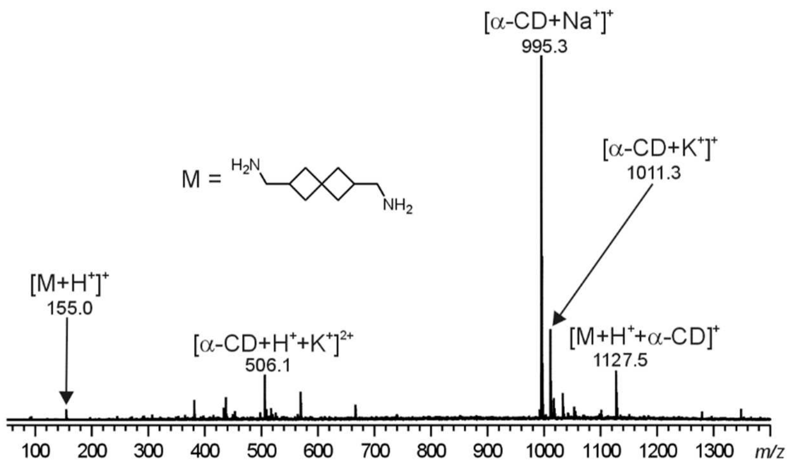

Figure S96 Mass spectrum of a mixture of the guest **8a** and α-CD.

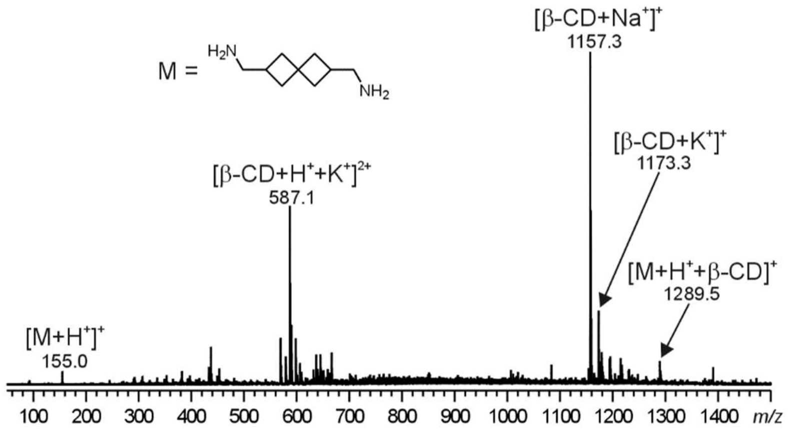

Figure S97 Mass spectrum of a mixture of the guest **8a** and β-CD.

Isothermal titration calorimetry data

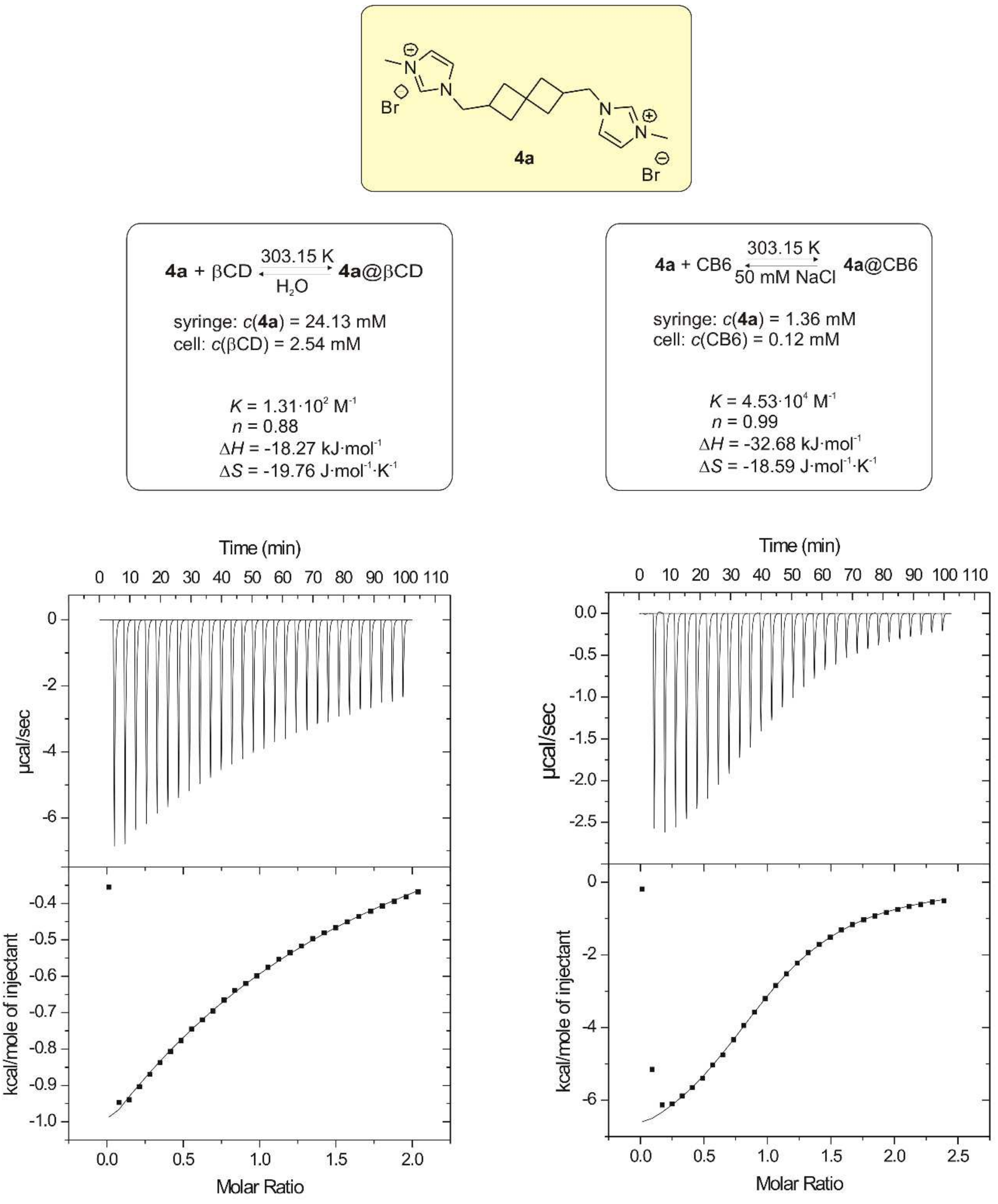

Figure S98 Typical ITC data for the guest **4a** and β-CD (left) and CB6 (right).

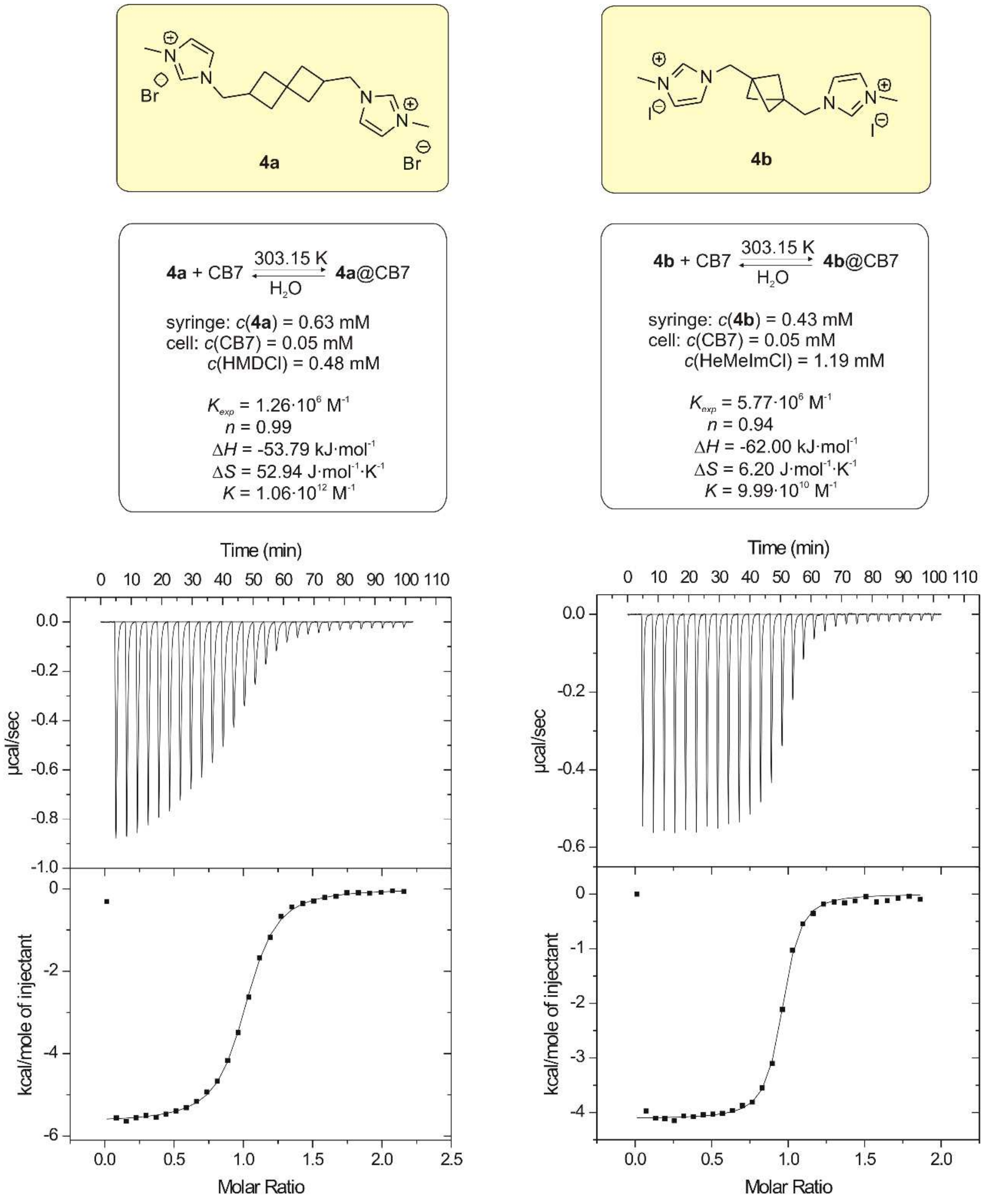

Figure S99 Typical ITC data for the guests **4a** (left) and **4b** (right) with CB7.

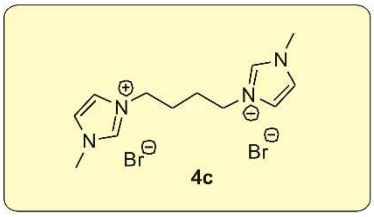

4c + CB6 $\underset{\text{50 mM NaCl}}{\overset{\text{303.15 K}}{\rightleftharpoons}}$ **4c**@CB6

syringe: c(**4c**) = 0.62 mM
cell: c(CB6) = 0.04 mM
c(4d) = 0.59 mM

K_{exp} = 1.93·10^{6} M^{-1}
n = 1.03
ΔH = -47.58 kJ·mol^{-1}
ΔS = -9.45 J·mol^{-1}·K^{-1}
K = 5.01·10^{7} M^{-1}

4c + CB7 $\underset{H_2O}{\overset{\text{303.15 K}}{\rightleftharpoons}}$ **4c**@CB7

syringe: c(**4c**) = 0.52 mM
cell: c(CB7) = 0.06 mM
c(dop) = 2.30 mM

K_{exp} = 2.85·10^{6} M^{-1}
n = 0.98
ΔH = -40.87 kJ·mol^{-1}
ΔS = 47.57 J·mol^{-1}·K^{-1}
K = 3.32·10^{9} M^{-1}

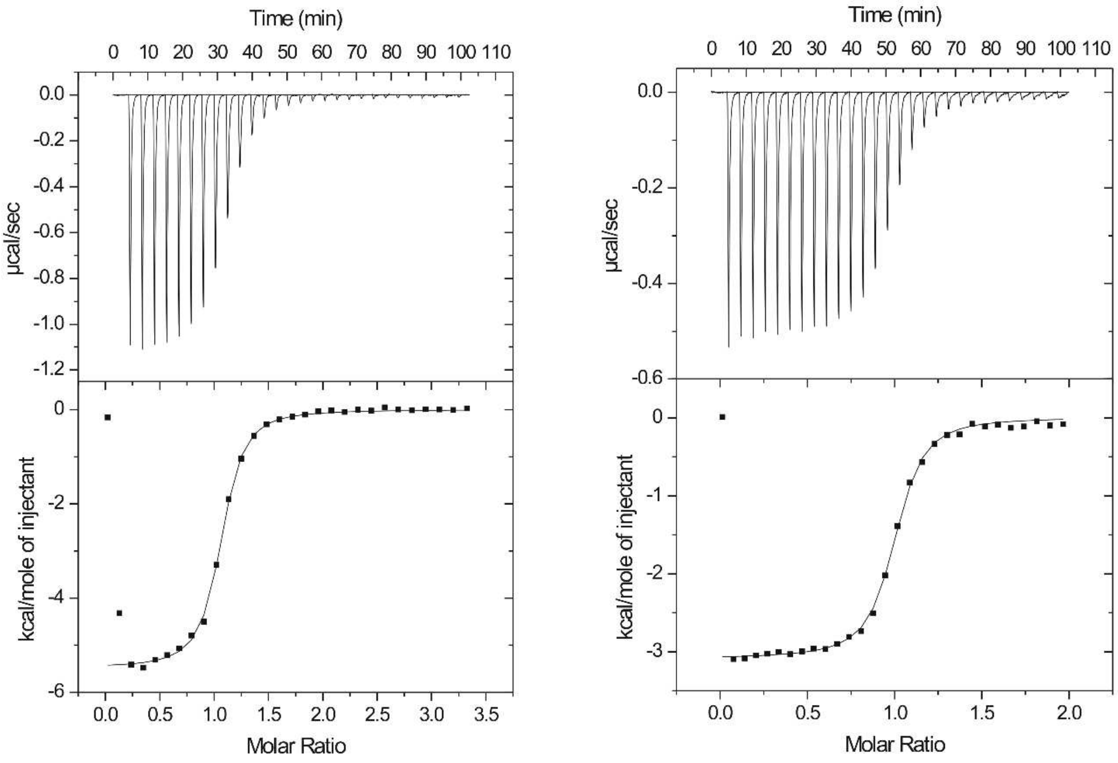

Figure S100 Typical ITC data for the guest **4c** and CB6 (left) and CB7 (right).

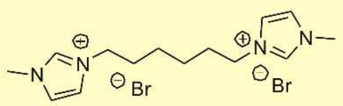

4d

$$\mathbf{4d} + \text{Cb6} \underset{\text{50 mM NaCl}}{\overset{\text{303.15 K}}{\rightleftharpoons}} \mathbf{4d}@\text{CB6}$$

syringe: c(**4d**) = 1.90 mM
cell: c(CB6) = 0.17 mM

$K = 3.92 \cdot 10^{4}$ M^{-1}
n = 0.99
ΔH = -22.92 $kJ \cdot mol^{-1}$
ΔS = 12.39 $J \cdot mol^{-1} \cdot K^{-1}$

$$\mathbf{4d} + \text{CB7} \underset{H_2O}{\overset{\text{303.15 K}}{\rightleftharpoons}} \mathbf{4d}@\text{CB7}$$

syringe: c(**4d**) = 0.57 mM
cell: c(CB7) = 0.05 mM
c(TBAI) = 4.30 mM

$K_{exp} = 8.91 \cdot 10^{5}$ M^{-1}
n = 0.99
ΔH = -35.64 $kJ \cdot mol^{-1}$
ΔS = 23.77 $J \cdot mol^{-1} \cdot K^{-1}$
$K = 2.38 \cdot 10^{7}$ M^{-1}

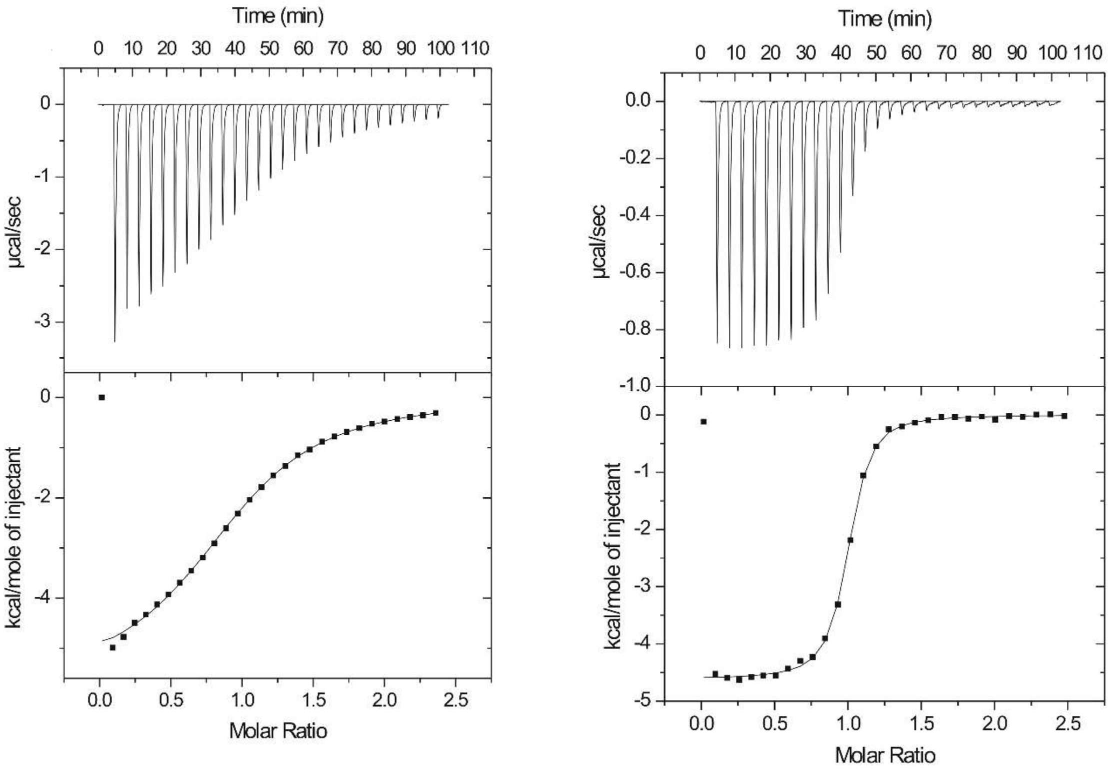

Figure S101 Typical ITC data for the guest **4d** and CB6 (left) and CB7 (right).

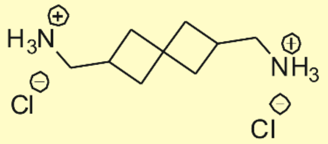

8a

$$\mathbf{8a} + \mathrm{CB7} \underset{H_2O}{\overset{303.15\ K}{\rightleftharpoons}} \mathbf{8a}@\mathrm{CB7}$$

syringe: c(**8a**) = 0.50 mM
cell: c(CB7) = 0.05 mM
c(HMDCl) = 0.75 mM

$K_{exp} = 2.71 \cdot 10^{6}\ M^{-1}$
$n = 1.00$
$\Delta H = -50.88\ kJ \cdot mol^{-1}$
$\Delta S = 73.98\ J \cdot mol^{-1} \cdot K^{-1}$
$K = 4.19 \cdot 10^{12}\ M^{-1}$

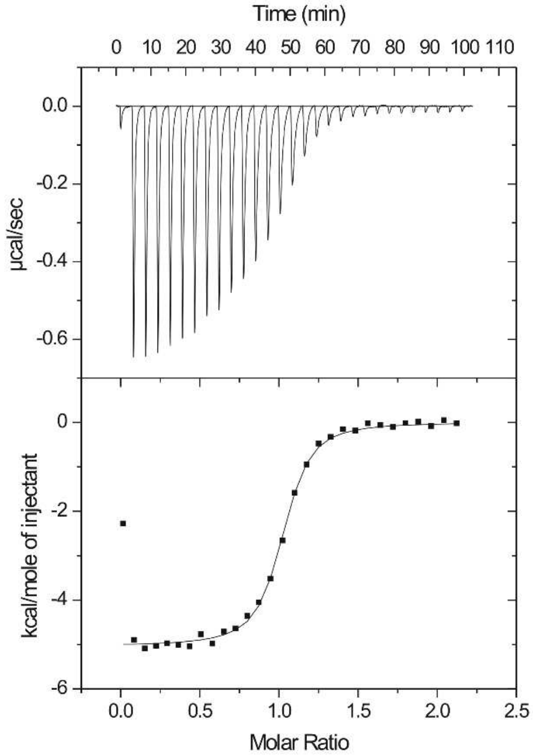

Figure S102 Typical ITC data for the guest **8a** and CB7.

Table S1 Thermodynamic data[a]

guest	host	K [dm^3·mol^{-1}]	n	$-\Delta H$ [kJ·mol^{-1}]	ΔS [J·mol^{-1}·K^{-1}]	$-\Delta G$ [kJ·mol^{-1}]	competitor (K [M^{-1}])
**4a**	α-CD				no binding		
	β-CD	$(1.35\pm0.04)\times10^{2}$	1.06±0.17	17.3±0.9	−24.4	12.36±0.07	na
	CB6[b]	$(4.0\pm0.7)\times10^{4}$	1.01±0.02	33.1±0.5	−20.2	27.01±0.01	na
	CB7	$(1.2\pm0.2)\pm10^{12}$	1.03±0.01	54.3±1.3	51.0	70±3	hexamethylenediammonium dichloride, $(2.1\pm0.2)\times10^{9}$
**8a**	α-CD				no binding		
	β-CD						
	CB6[b, c]	$(1.36\pm0.14)\times10^{6}$	nd	nd	nd	28.1±0.4	na
	CB7	$(3.8\pm0.4)\times10^{12}$	1.03±0.01	51.8±0.9	60.8	72±3	hexamethylenediammonium dichloride, $(2.1\pm0.2)\times10^{9}$
**4b**	α-CD				no binding		
	β-CD						
	CB6[b]						
	CB7	$(1.1\pm0.2)\times10^{11}$	0.95±0.01	61.6±0.9	9.5	64±3	1-hexyl-3-methylimidazolium chloride, $(1.46\pm0.06)\times10^{7}$
**4c**	α-CD				no binding		
	β-CD						
	CB6[b]	$(5.1\pm0.4)\times10^{7}$	0.97±0.06	47.8±1.9	−9.9	44±3	**4d**
	CB7	$(2.3\pm0.5)\times10^{9}$	0.99±0.04	40.9±1.6	44.3	54±5	dopamine hydrochloride, $(6.0\pm1.3)\times10^{5}$
**4d**	α-CD				no binding		
	β-CD						
	CB6[b]	$(4.2\pm0.4)\times10^{4}$	0.96±0.06	24.2±1.4	8.4	26.8±0.2	na
	CB7	$(2.35\pm0.07)\times10^{7}$	0.95±0.03	37.0±1.1	20.9	43.3±0.3	$Bu_4N^+I^-$, $(6.6\pm0.5)\times10^{3}$
**4g**	CB6[b]	1.6×10^{9}	0.98	37.2	53.6	53.4	**4d**
	CB7	2.1×10^{6}	1.04	17.5	63.6	36.7	na
**4h**	CB6[b]	7.9×10^{9}	1.03	42.6	49.0	57.4	1-[4-(1-adamantylcarbonyl)benzyl]-3-butylimidazolium bromide 1.43×10^{6}
	CB7	$(2.1\pm0.2)\times10^{9}$	0.98±0.03	29.8±0.9	80.1	54.0±1.4	cyclopentanone $(3.33\pm0.42)\times10^{5}$

[a] All measurements were carried out in the water at 303.15 K if not stated otherwise. [b] Performed in a 50 mM NaCl solution in water.
[c] Determined from ^{1}H NMR spectra. na = not applicable. nd = not determined.

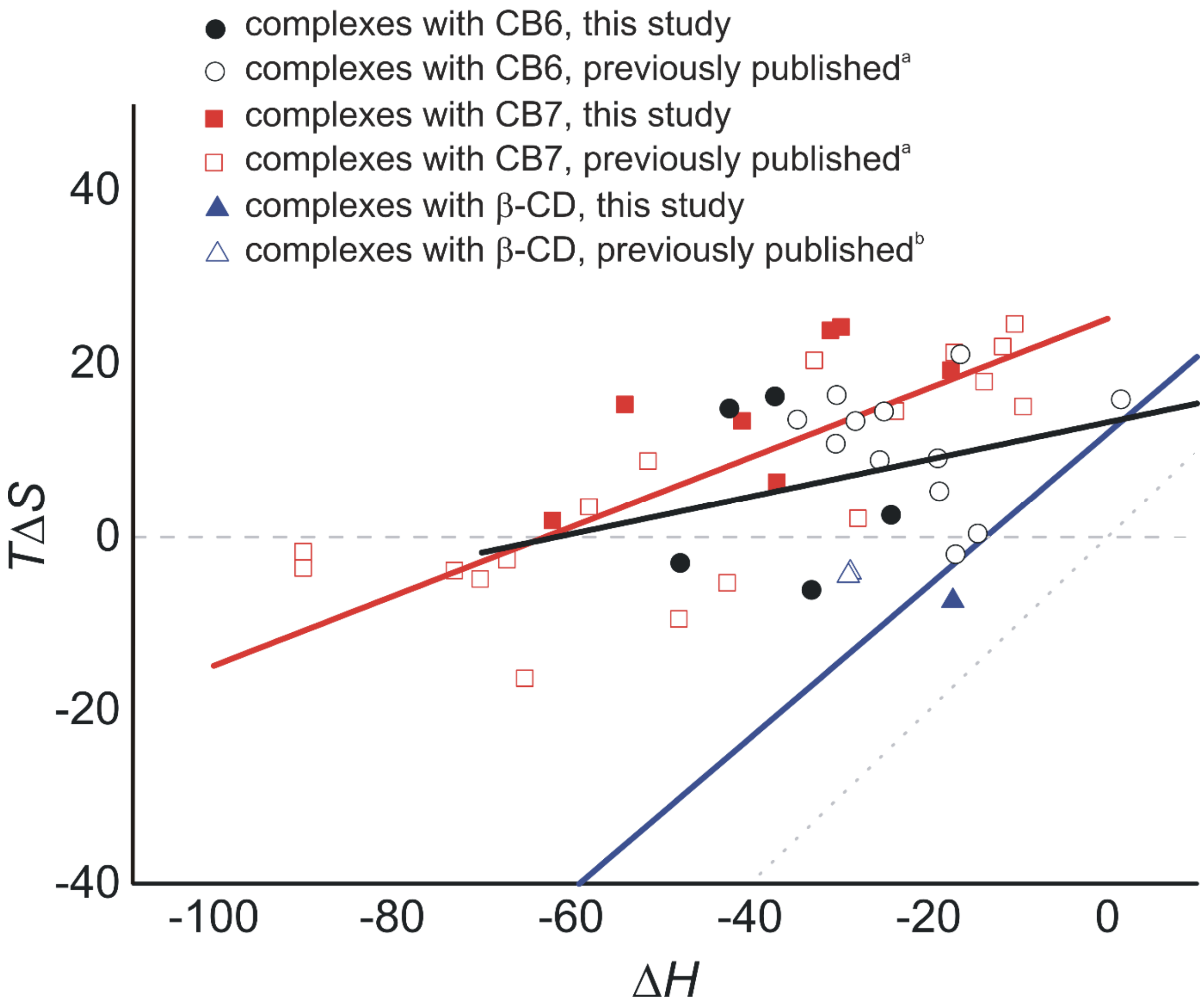

Figure S103 Enthalpy[kJ·mol^{-1}]–entropy[kJ·K^{-1}·mol^{-1}] compensation plot. Blue solid line = linear regression with data for natural cyclodextrins as published by Rekharsky ($Y(x)$=12+0.88X).[1] Red and black solid lines = linear regression with CB7 and CB6 data, respectively. [a] Data were taken from http://suprabank.org for guests with two positively charged axially arranged substituents. [b] Our previously published data in addition to Rekharsky's set.[2] Dotted grey line = $Y(x)$= X; dashed grey line = $Y(x)$=0.

[1] Rekharsky, M. V.; Inoue, Y. Complexation Thermodynamics of Cyclodextrins. *Chem. Rev.* **1998**, *98*, 1875–1918.
[2] Babjaková, E.; Branná, P.; Kuczyńska, M.; Rouchal, M.; Dastychova, L.; Vícha, J.; Vícha, R. An adamantane-based disubstituted binding motif with picomolar dissociation constants for cucurbit[*n*]urils in water and related quaternary assemblies. *RSC Adv.* **2016**, *6*, 105146–105153.

Single-crystal X-ray diffraction data

Table S2 Crystal data and structure refinement of **4b** and **4a**@CB6.

Compound	**4b**	**4a**@CB6
CCDC deposition number	2271806	2171957
Empirical formula	$C_{15}H_{22}N_4I_2$	$C_{53}H_{62}Br_2N_{28}O_{12}$
Formula weight (g·mol^{-1})	512.17	1443.12
Colour; shape	Colourless; plate	Colourless; block
Crystal size	0.29 × 0.25 × 0.07	0.050 × 0.050 × 0.020
Source; λ[Å]	Cu $K\alpha$; 1.54184	Cu $K\alpha$; 1.54184
Measured temperature (K)	120 (2)	120 (2)
Crystal system	Orthorhombic	Triclinic
Space group	$P2_12_12$	$P\overline{1}$
Unit cell dimensions (Å, °)	a = 11.6757(3)	a = 13.0933(2)
	b = 28.7227(5)	b = 22.0550(2)
	c = 5.5444(2)	c = 24.8379(2)
		α = 112.9370(10)
	$\alpha = \beta = \gamma$ = 90	β = 103.1210(10)
		γ = 96.5930(10)
Volume (Å^3)	1859.36(9)	6265.54(13)
Z	4	4
D_x (g·cm^{-3})	1.830	1.530
μ (mm^{-1})	26.559	2.356
Absorption correction	0.382 to 1.000	0.86022 to 1.00000
F(000)	984	2968
θ Range (°)	3.077 to 69.994	3.533 to 68.250
Completeness to θ (%)	0.998	0.997
h	$-14 \leq h \leq 10$	$-15 \leq h \leq 15$
k	$-35 \leq k \leq 32$	$-26 \leq k \leq 26$
l	$-6 \leq l \leq 6$	$-29 \leq l \leq 29$
Reflections collected	7047	157955
Reflections unique	3536 [R(int) = 0.0576]	22882 [R(int) = 0.0680]
Unique reflections with $I \geq 2\sigma(I)$	2978	18504
Number of parameters	193	1918
Goodness-of-fit on F^2	1.041	1.050
Final R indices [$I \geq 2\sigma(I)$]	R_1 = 0.0653, wR_2 = 0.1709	R_1 = 0.0620, wR_2 = 0.1600
R indices (all data)	R_1 = 0.0710, wR_2 = 0.1774	R_1 = 0.0744, wR_2 = 0.1673
Residual highest peak and deepest hole (e·Å^{-3})	3.322 and −1.189	1.152 and −1.526

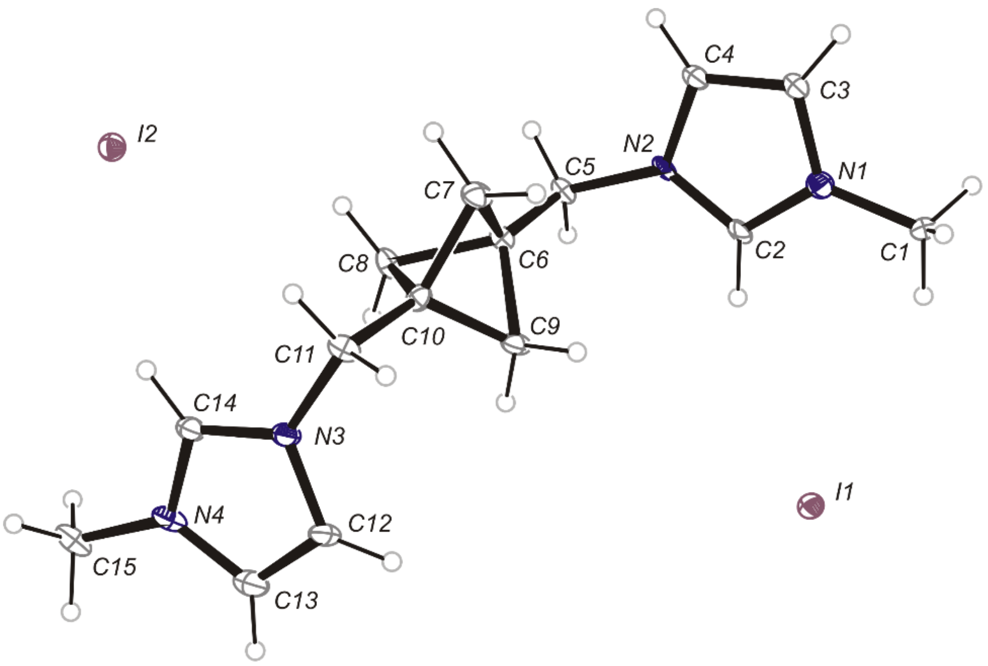

Figure S104 *ORTEP* (30% probability) for the guest **4b**

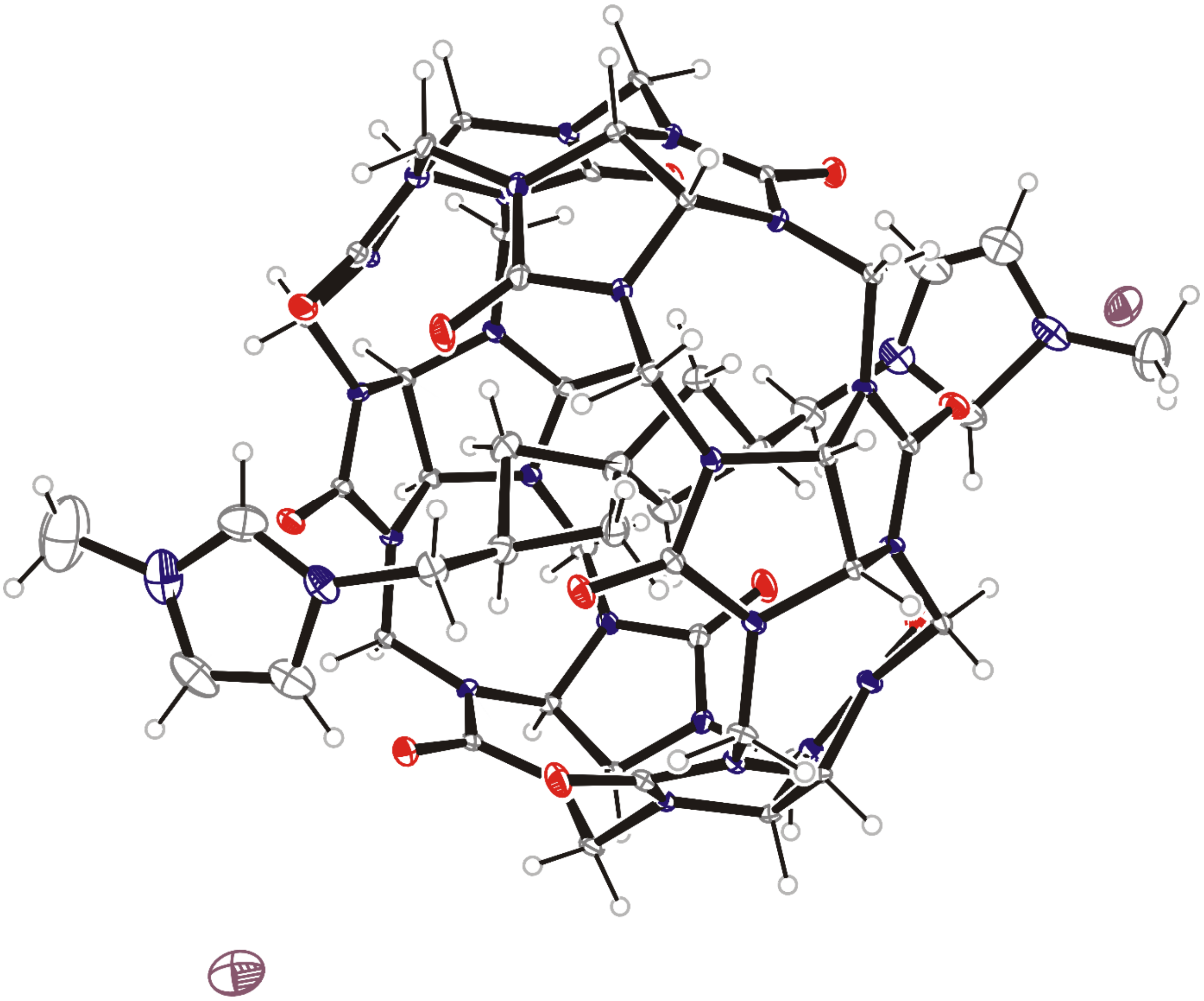

Figure S105 *ORTEP* (30% probability) for the complex **4a**@CB6. The major component of disordered atoms is displayed for clarity.

Experimental details for compound **4b**

A single crystal of **4b** was prepared by slow evaporation of H_2O from the solution at room temperature. X-ray diffraction data were collected on a dual-source Rigaku SuperNova diffractometer equipped with an Atlas detector and an Oxford Cryostream cooling system using mirror-monochromated Cu *K*α radiation (λ = 1.54184 Å). Data collection and reduction were performed using the program CrysAlisPro[1] and Gaussian face-index absorption correction method was applied.[1] The structures were solved with Direct Methods (SHELXS)[2–4] and refined by full-matrix least-squares based on F^2 using SHELXL2015.[2–4] Non-hydrogen atoms were assigned anisotropic displacement parameters unless stated otherwise. Other hydrogen atoms were placed in idealized positions and included as riding. Isotropic displacement parameters for all H atoms were constrained to multiples of the equivalent displacement parameters of their parent atoms with U_{iso}(H) = 1.2 U_{eq}(parent atom).

Experimental details for complex **4a**@CB6

A single crystal of **4a**@CB6 was prepared by slow evaporation of H_2O from the solution at room temperature. X-ray diffraction data were collected on a Rigaku Synergy diffractometer with a hybrid pixel array detector and kappa goniometer, using Cu *K*α radiation from a rotating anode X-ray source. The temperature during data collection was 120 K. The structures were solved by intrinsic phasing and refined by full-matrix least-squares methods on F^2 using SHELXT[4] and SHELXL[5]. All non-hydrogen atoms were refined anisotropically, and the disordered moieties were refined using restraints on geometric and displacement parameters. All hydrogen atoms were placed in geometrically calculated positions and refined as riding on their carrier atoms. Several residual density maxima in the difference density maps suggested the presence of disordered water molecules, and the final refinement cycles were done after the SQUEEZE[6] routine implemented in PLATON was used to reduce the disordered solvent contribution from the structure factor calculations.

---

[1] Rigaku Oxford Diffraction, 2017, CrysAlis^{Pro} software system, version 38.46, Rigaku Corporation, Oxford, UK.
[2] G. M. Sheldrick, A short history of ShelX. *Acta Cryst.* **2008**, *A64*, 112-122.
[3] G. M. Sheldrick, SHELXL13. Program package for crystal structure determination from single crystal diffraction data, University of Göttingen, Germany, 2013.
[4] G. M. Sheldrick: SHELXT - Integrated space-group and crystal-structure determination. *Acta Cryst.* **2015**, *A71*, 3–8, DOI: 10.1107/S2053273314026370.
[5] G. M. Sheldrick: Crystal structure refinement with SHELXL. *Acta Cryst.* **2015**, *C71*, 3–8, DOI: 10.1107/S2053229614024218.
[6] A. L. Spek: PLATON SQUEEZE: a tool for the calculation of the disordered solvent contribution to the calculated structure factors. *Acta Cryst.* **2015**, *C71*, 9–18, DOI: 10.1107/S2053229614024929.
